# Supplementary material for: Polymer microbubbles as universal platform to accelerate polymer mechanochemistry
Source: Nat Commun. 2025 Jun 25;16:5380. doi: 10.1038/s41467-025-60667-8 (PMC12198389; doi:10.1038/s41467-025-60667-8)
Supplement: Supplementary file 1 — Supplementary Information [file 41467_2025_60667_MOESM1_ESM.pdf]

Supplementary Information

for

Polymer microbubbles as universal platform to accelerate polymer mechanochemistry

Jilin Fan[1,2], Regina Lennarz[3], Kuan Zhang[1,2,4], Ahmed Mourran[2], Jan Meisner[3], Mingjun Xuan[1,2,4,*], Robert Göstl[1,2,5,*], Andreas Herrmann[1,2,*]

[1]Institute of Technical and Macromolecular Chemistry, RWTH Aachen University, 52074 Aachen, Germany
[2]DWI – Leibniz Institute for Interactive Materials, 52056 Aachen, Germany
[3]Institute for Physical Chemistry, Heinrich Heine University Düsseldorf, 40225 Düsseldorf, Germany
[4]Wenzhou Institute, University of Chinese Academy of Sciences, 325001 Wenzhou, China
[5]Department of Chemistry and Biology, University of Wuppertal, 42119 Wuppertal, Germany

Content

1. Materials and methods 3
  1.1. Materials 3
  1.2. Methods 3
    1.2.1. Analytical instrumentation 3
    1.2.2. Fluorescence spectroscopy 4
    1.2.3. MTS proliferation assays 5
    1.2.4. Microfluidic production 5
    1.2.5. Computational simulations 5
2. Synthetic procedures and characterization data 7
  2.1. Synthetic routes overview 7
  2.2. Detailed synthetic procedures 8
  2.3. Preparation of linear polymers 18
3. Preparation of polymeric microbubbles and microgels 19
  3.1. Preparation of PMBs with masked dansyl-fluorophore (PMB-MDF) 19
  3.2. Preparation of PMB-UMB or PMB-CPT 19
  3.3. Preparation of PMB-PBL 20
  3.4. Preparation of PMB-Flex 20
  3.5. Preparation of microgels 21
    3.5.1. Preparation of microgels with masked dansyl-fluorophore 21
    3.5.2. Preparation of microgels with flex-mechanophores (microgels-Flex) 21
4. Supplementary figures 22
5. NMR and ESI-MS spectra 42
6. Supplementary tables 68
7. Supplementary references 71

1. Materials and methods

1.1. Materials

All chemical reagents and solvents were used without further purification unless otherwise stated. Dansyl chloride (98%), furfurylamine (99%), 2-isocyanatoethyl methacrylate (≤ 0.1% BHT, 98%), ethyl propiolate (99%), lithium bis(trimethylsilyl)amide solution (LHDMS, 1.0 M in THF), toluene (≥99.5%), 2-hydroxyethyl disulfide (technical grade), methacryloyl chloride (97%), triethylamine (TEA, ≥99%), dichloromethane (CH_2Cl_2, anhydrous, ≥99.8%), MeCN (99.8%), *N*,*N*'-disuccinimidyl carbonate (DSC, ≥95%), methylmagnesium bromide (3.0 M in Et_2O), Et_2O (99.7%), 1-pyrenebutanol (PBL, 99%) 1-pyrenebutyric acid (97%), DMSO (99.9%), $CuBr_2$ (99%), Me_6TREN (97%), acetylenedicarboxylic acid (95%), 1,6-hexanediol (97%), benzene (99.8%), α-bromoisobutyryl bromide (98%), 5-(hydroxymethyl)furfural (99%), triphosgene (TPS, 99%), 1,6-hexanediol dimethacrylate (≥90%), poly(propylene glycol) diacrylate (PPGDA, average M_n ~800 Da), poly(propylene glycol) (PPG, average M_n ~2,000 Da), poly(vinyl alcohol) (PVA, M_w ~31,000 to 50,000 Da), glycerol (99.5%), 2-mercaptoethanol (MCE, 99%), were obtained from Sigma-Aldrich.

Ethyl(2,4,6-trimethylbenzoyl)phenylphosphinate (TPO-L, 95%, Fluorochem), umbelliferon (UMB, 98%, Acros), camptothecin (CPT, 95%, abcr), 4-(dimethylamino)pyridine (DMAP, >99.0%, TCI Deutschland GmbH), *p*-toluenesulfonic acid monohydrate (PTSA, 98%, TCI Deutschland GmbH), *N*-(2-hydroxyethyl)maleimide (98%, TCI Deutschland GmbH), were used as received. Poly(ethylene glycol) methyl ether methacrylate (PEGMEMA, M_n ~ 300 Da, Sigma-Aldrich) was purified by a column of activated basic Al_2O_3 to remove the inhibitor. Dialysis membranes (3.5 kDa MWCO) were obtained from Spectrum Labs. Centrifugal filter (3000 kDa MWCO) was obtained from Sartorius. Ultrapure water (18.2 MΩ·cm) was used for all experiments.

1.2. Methods

1.2.1. Analytical instrumentation

NMR

^{1}H and ^{13}C NMR spectra were recorded at room temperature in $CDCl_3$ on a 400 MHz Bruker Avance 400 spectrometer (^{13}C: 101 MHz). The chemical shifts are reported in *δ* units using residual protonated solvent signals as internal standard (^{1}H: $CDCl_3$ ($^{\delta}$H = 7.26 ppm), ^{13}C: $CDCl_3$ ($^{\delta}$C = 77.16 ppm); ^{1}H: $(CD_3)_2SO$ ($^{\delta}$H = 2.50 ppm), ^{13}C: $(CD_3)_2SO$ ($^{\delta}$C = 39.53 ppm)). The following abbreviations were used: s = singlet, d = doublet, t = triplet, q = quartet, sept. = septet, dd = doublet of doublets etc., m = multiplet. Coupling constants (*J*) were given in Hz and refer to the respective H, H-couplings.

ESI-MS

Electrospray ionization (ESI) MS: micrOTOF-Q II™ ESI-Qq-TOF mass spectrometer system (BRUKER).

TLC and column chromatography

Thin layer chromatography (TLC) were performed on Merck TLC Silica gel 60 F_{254} TLC plates with a fluorescence indicator employing a 254 nm or 365 nm UV lamp for visualization. Silica gel for chromatography (40-63 µm) was used for flash column chromatography.

GPC

Gel permeation chromatography (GPC) with THF (HPLC grade) was performed using a HPLC pump (PU-2080plus, Jasco) equipped with a refractive index detector (RI-2031plus, Jasco). The sample solvent contained 250 mg·mL^{-1} 3,5-di-*t*-4-butylhydroxytoluene (BHT, ≥99%, Fluka) as internal standard. One pre-column (8×50 mm) and four SDplus gel columns (8×300 mm, SDplus, MZ Analysentechnik) were applied at a flow rate of 1.0 mL·min^{-1} at 20 °C. The

diameter of the gel particles was 5 µm, the nominal pore widths were 50, 102, 103, and 104 Å. Calibration was achieved using narrowly distributed poly(methyl methacrylate) (PMA) standards (Polymer Standards Service). Molar masses (M_n and M_w) and molar mass dispersities ($Đ_M$ = M_w/M_n) were calculated by using the PSS WinGPC UniChrom software (version 8.1.1).

SEM

Scanning electron microscopy (SEM) images were captured by Ultra High Resolution SEM (HITACHI, SU9000). Preparation of SEM samples: one drop (~10 µL) of sample was deposited onto the clean silicon wafers and are dried under room temperature. These silicon wafers with samples are coated with a thin conductive carbon layer (3 nm) and then characterized by SEM.

UHPLC

Ultra-high-performance liquid chromatography-mass spectrometry (UHPLC-MS) system: ACQUITY UPLC I-Class System (Waters) with the compatible ACQUITY UPLC PDA eλ Detector and ACQUITY QDa detector (Waters). Solvents: A= water (contained 0.1% TFA), B= acetonitrile (contained 0.1% TFA); Flow= 0.4 mL·min^{-1}; Gradient (B): 0-1 min (10%), 1-5 min (10% - 90%), 5-7 min (90%), 7-10 min (90% - 10%).

AFM

Atomic force microscopy (AFM) Measurements: The prepared PMBs was dropwise onto a Silica wafer and dried at room temperature. The stiffness of the PMBs was measured with an atomic force microscope (Bruker, Dimension Icon FastScan System) performed in the air in force-volume mode. While scanning, force-distance curves were recorded at different indentation loads. The stiffness of PMBs were assessed by measuring the slope of the force-distance curve using Nanoscope Analysis Software.

CLSM

Optical microscopy: images of PMBs were captured by a confocal laser scanning microscope (STP8, Leica) with a 10x objective (HC PL FLUOTAR, Leica).

1.2.2. Fluorescence spectroscopy

Fluorescence spectra were measured by SpectraMax iD3 Multi-Mode Microplate Reader (Molecular Devices) at room temperature. The fluorescence spectra measurements from PMBs-MDF, samples were excited at 340 nm. The spectral bandwidths were set to 5 nm (400 nm to 750 nm) for emission. To obtain the standard curve of dansyl-fluorophore (**N1-A3**), the fluorescence intensity at the emission wavelength of 545 nm was collected. The fluorescence spectra measurements of PMB-UMB were excited at 325 nm. The spectral bandwidths were set to 5 nm (370 to 650 nm) for emission. To obtain the standard curve of UMB, the fluorescence intensity at the emission wavelength of 465 nm was collected. The fluorescence spectra measurements of PMB-CPT, samples were excited at 335 nm. The spectral bandwidths were set to 5 nm (375 to 600 nm) for emission. To obtain the standard curve of CPT, the fluorescence intensity at the emission wavelength of 450 nm was collected. The fluorescence spectra measurements of PMB-PBL, samples were excited at 320 nm. The spectral bandwidths were set to 5 nm (360 to 500 nm) for emission. To obtain the standard curve of PBL, the fluorescence intensity at the emission wavelength of 390 nm was collected. The fluorescence spectra measurements of PMB-Flex, samples were excited at 340 nm. The spectral bandwidths were set to 5 nm (400 nm to 750 nm) for emission. All sonicated PMBs samples were stirred for 72 h to complete the downstream release reactions before the fluorescence measurements. The integration time was 0.1 s and all spectroscopic measurements were carried out with the pureGrade™ 96-wells plate purchased from BRAND GmbH. For the sonicated solution of PMBs, filtration steps were needed to obtain supernatant before the fluorescence measurements. Filtration was carried out as follows: Centrifugation at

RCF 8000 ×g for 15 min, then the suspensions were filtered through a centrifugal filter (3,000 Da MWCO) by centrifugation at 5,000 rpm.

1.2.3. MTS proliferation assays

Cell viability was studied by culturing HeLa cells with different samples in a basal medium containing DMEM (supplemented with 10% fetal bovine serum and 1% antibiotics/antimycotics) at 37 °C. The measurement of cell viability was implemented by using a commercial kit, which contain tetrazolium compound 3-(4,5-dimethylthiazol-2-yl)-5-(3-carboxymethoxyphenyl)-2-(4-sulfophenyl)-2H-tetrazolium (inner salt, MTS reagent) and a chemical electron acceptor dye (phenazine ethosulfate; PES) (Promega, Germany). Briefly, approximately 5,000 cells in 100 µL of medium were seeded into 96-well plates. After overnight incubation, the culture medium in 96-well plates was removed and exchanged with fresh medium (100 µL) containing different concentrated testing samples. Control cultures were treated with DMSO alone. The final concentration of DMSO in the medium was controlled by less than 0.5%. After 48 h incubation, cell culture media were removed, the cells were rinsed with 100 µL PBS buffer, and then 20 µL MTS reagent with 100 µL fresh medium was added to the cells. The resulting solution was mixed thoroughly, and the absorbance was measured using a microplate spectrophotometer at 490 nm (Synergy™ HT microplate reader, BioTek Instruments). MTS signals represented the survival and proliferation determination. The mixture of MTS reagent with cell culture medium was served as negative control. All the sample cultures were performed at least in triplicates.

1.2.4. Microfluidic production

Preparation of microfluidic device

The geometry of microfluidic channel was printed on glass slide using the high-resolution 3D printer (Photonic Professional GT2, Nanoscribe GmbH & Co. KG) with a 25× objective. Then, the glass slide was transferred into a PGMEA bath to remove uncured photoresist and develop the printed geometry patterns. Isopropanol was used to wash the glass slide. SYLGARD™ 184 silicone elastomer kit (Dow Corning) was poured on the glass slide and cured for 4 h at 60 °C. After cooling, PDMS replica was peeled off from the slide. Then, PDMS replica was punched to produce inlets and outlet. Subsequently, the PDMS replica was assembled with a glass slide treated by oxygen plasma. Lastly, the assembly was heated in an oven for 3 d at 60 °C to obtain the microfluidic device.

Dopamine was used for the surface hydrophilisation process of microfluidic channel. 2.0 $mg \cdot mL^{-1}$ dopamine solution (pH 8.0 Tris buffer) is filled to the microfluidic channel and kept for 30 min. Then, DI water is injected to flush the microchannel twice. Finally, a hydrophilic-treated chip was obtained (Supplementary Figure).

Microfluidic station

A self-made microfluidic station was used to prepare the microbubbles and microgels. The microfluidics station consisted of a computer, microscope, light set, syringe pump, flow controller, and microfluidic device (Supplementary Figure). Syringe pump was used to control the flow rate of oil phase injected into the microfluidic device. Flow controller equipped with a digital pressure valve was used to control the flow rate of gas or aqueous phase solution pumped into the microfluidic device.

1.2.5. Computational simulations

Model for simulating uniaxial pressure

The simulation of uniaxial pressure onto the mechanophore incorporated into the PMBs was conducted using the model for uniaxial compression based on the *Generalized Force-modified potential energy surface* (G-FMPES), in which an additional term V_{ext} is added to the *ab initio* potential $V_{ab\ initio}$. Every atom is pushed individually towards the *xy*-plane by a harmonic

potential. The external potential V_{ext} (Eq. S1) is therefore the sum of the *z*-components of each individual atom, multiplied by a force constant, which is why it depends on the distance of all *N* atoms to the *xy*-plane.

$$V_{ext} = \frac{k}{2} \cdot \sum_{i=1}^{N} z_i^2 \qquad \text{(S1)}$$

In the equation above, z_i is defined as the distance of the *i*-th atom to the *xy*-plane ($z = 0$) while *k* is a force constant defining the strength of the uniaxial force. The external force $\mathbf{F}_{ext}$ can be derived from the external potential V_{ext} by the first order derivative of the potential with respect to the spatial coordinates (Eq. S2).

$$\mathbf{F}_{ext} = -\vec{\nabla}\, V_{ext} \qquad \text{(S2)}$$

Due to the quadratic dependence on z_i, this potential results in a higher force acting on atoms that are further away from the plane compared to those closer to it. Supplementary Figure 45 schematically illustrates the effect of the model on a system.

Using this model, it is possible to optimize minima as well as transition states on the G-FMPES. Therefore, insights on the reactivity of molecular systems under the impact of uniaxial pressure can be achieved.

Force-modified activation energies

Supplementary Table 4 shows the potential activation energies as well as the energies of the reactant and the transition state for the flex-activated reaction of the mechanophore in the PMBs under the impact of a pulling force. For the simulation of a pulling force the FMPES approach is used.

Supplementary Table 5 shows the potential activation energies as well as the energies of the reactant and the transition state for the flex-activated reaction of the mechanophore in the PMBs under the impact of uniaxial pressure in dependence on the force constant.

Force-modified geometries

In order to illustrate the effect of the uniaxial compression on the geometries of the reactant and the transition state, a selection of the DFT-computed structures is shown in an overlay in Supplementary Figure 46. For simplicity, the structures are reduced to the oxanorbornadiene-motif as this is the part of the molecule where the reaction of interest occurs. The optimized atomic coordinates of the electronic structure calculations are shown in Supplementary Data 1.

2. Synthetic procedures and characterization data

2.1. Synthetic routes overview

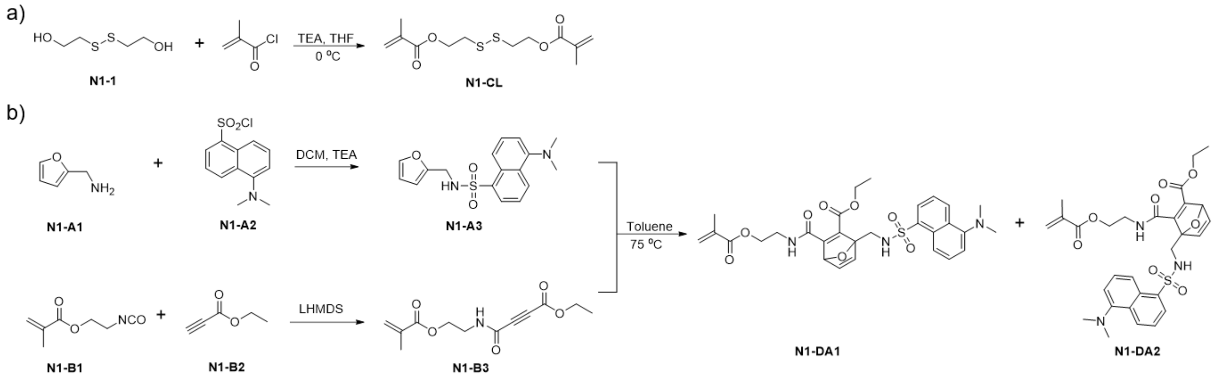

Supplementary Figure 1. Synthetic routes. (**a**) Synthesis of bis(2-methacryloyl)oxyethyl disulfide (**N1-CL**). (**b**) Synthesis of probe molecule (**N1-DA2**).

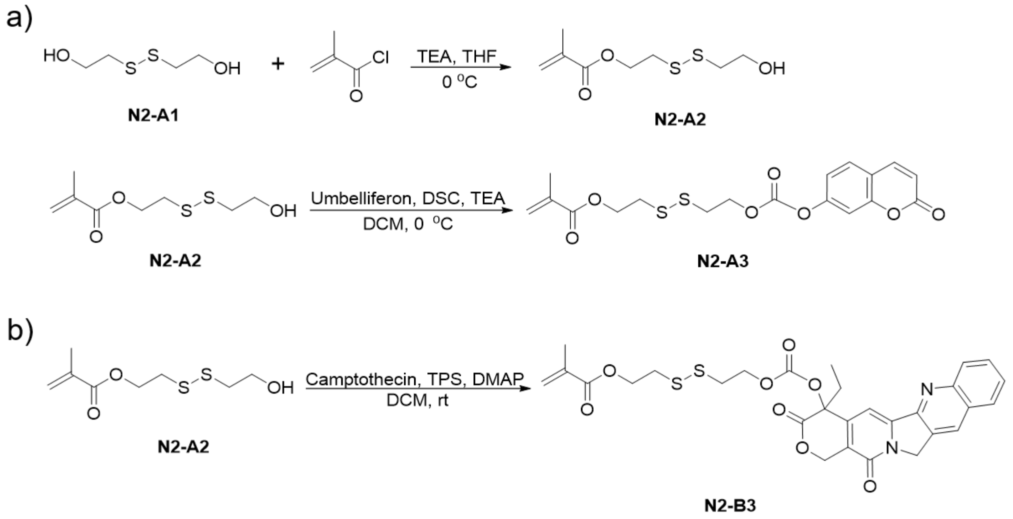

Supplementary Figure 2. Synthetic routes. (**a**) Synthesis of probe molecule (**N2-A3**). (**b**) Synthesis of prodrug (**N2-B3**).

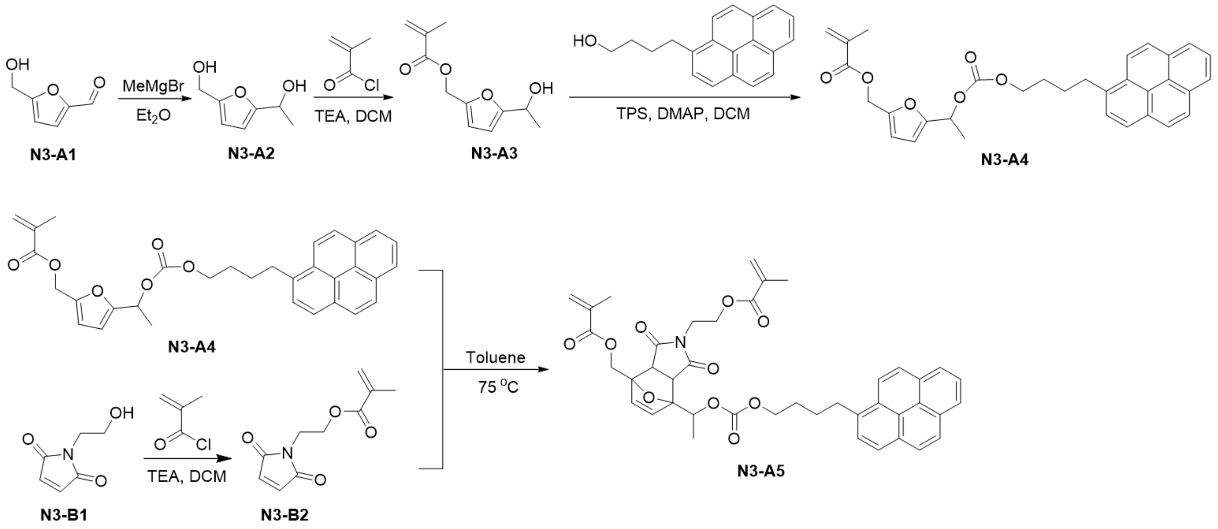

Supplementary Figure 3. Synthetic routes. Synthesis of probe molecule (**N3-A5**).

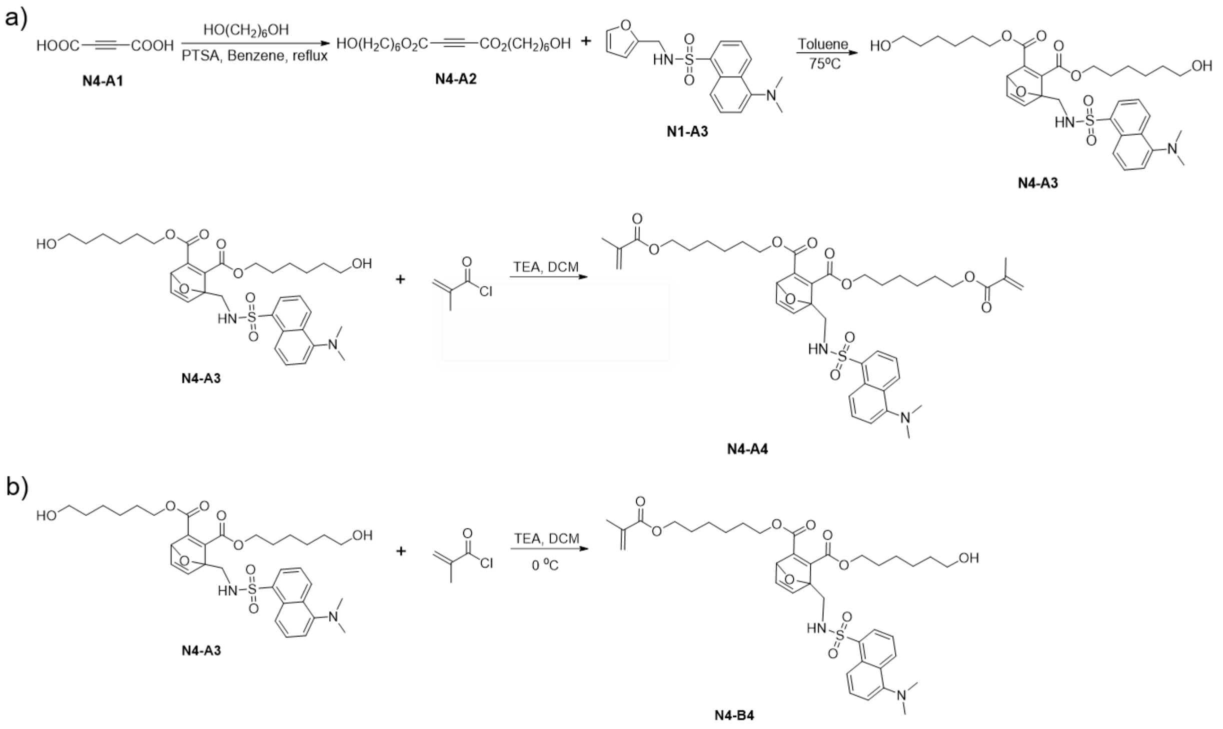

Supplementary Figure 4. Synthetic routes. (**a**) Synthesis of probe molecule (**N4-A4**). (**b**) Synthesis of one side acrylate-probe (**N4-B4**).

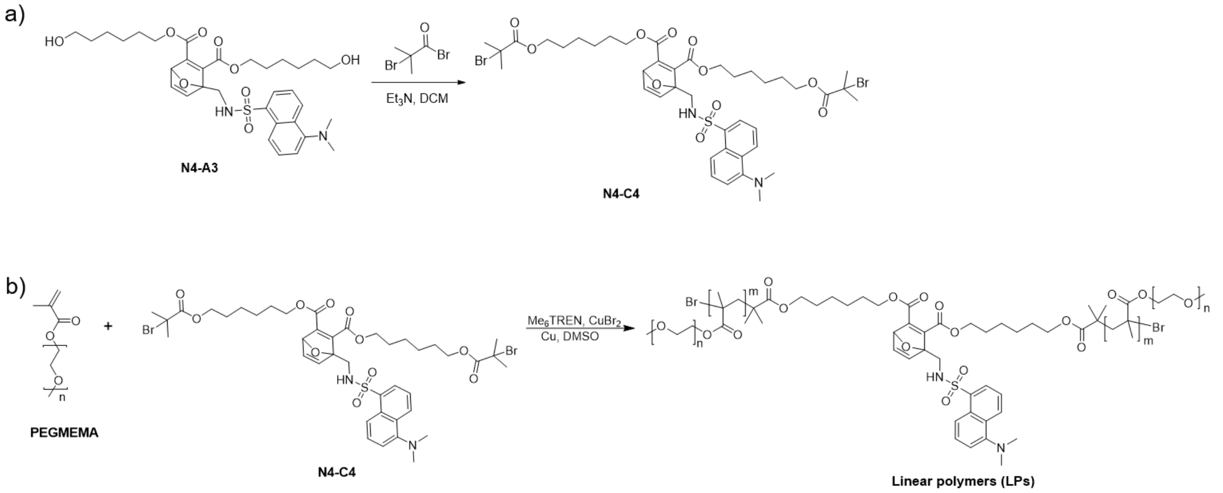

Supplementary Figure 5. Synthetic routes. (**a**) Synthesis of alkyl bromide (**N4-C4**). (**b**) Synthesis of linear polymers (**LPs**).

2.2. Detailed synthetic procedures

*Bis(2-methacryloyl)oxyethyl disulfide (**N1-CL**)*

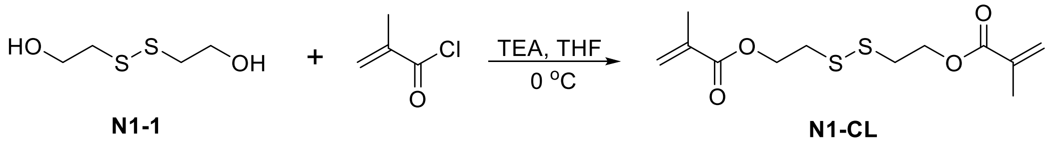

2-hydroxyethyl disulfide (2.0 g, 13 mmol, 1 equiv.) and TEA (3.64 mL, 26 mmol, 2.0 equiv.) were added in THF (30 mL) with a constant flow of N_2. The mixture in a flask was placed into the ice-water bath to keep the temperature at 0 °C. Afterwards, a solution of methacryloyl chloride (2.54 mL, 26 mmol, 2.04 equiv.) and 15 mL CH_2Cl_2 were added dropwise (15 mL/h) to the reaction mixture via an injection pump. The reaction mixture was stirred until complete consumption of the starting material, as indicated by TLC. Subsequently, the reaction mixture

was washed three times with brine and three times with H_2O. Afterwards, the crude product was purified by column chromatography on silica gel (hexane:EtOAc = 5:1) to give compound **N1-CL** (3.2 g, 85% yield) as a colourless oil. **^{1}H NMR (400 MHz, $CDCl_3$):** *δ* (ppm): 6.12 (sept, *J*=1.6 Hz, 2H), 5.58 (sept, *J*=1.6 Hz, 2H), 4.4 (t, *J*=6.8 Hz, 4H), 2.97 (t, *J*=6.8 Hz, 4H), 1.94 (s, *J*=1.2 Hz, 6H). **^{13}C NMR (101 MHz, $CDCl_3$):** *δ* (ppm): 167.23, 136.12, 126.13, 62.6, 37.39, 18.4. **ESI-MS** (*m/z*) for $C_{12}H_{18}O_4S_2$ expected $[M+Na]^+$: 313.0539, Found for $[M+Na]^+$: 313.0590.

*5-(Dimethylamino)-N-(furan-2-ylmethyl)naphthalene-1-sulfonamide (**N1-A3**)*

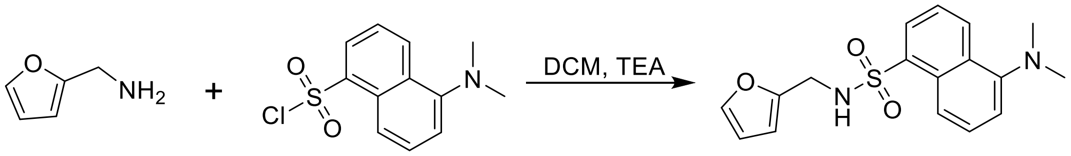

N1-A3

A mixture of 5-(dimethylamino)naphthalene-1-sulfonyl chloride (1.00 g, 3.70 mmol, 1.0 equiv.) and Et_3N (1.0 mL, 7.2 mmol, 2.0 equiv.) in CH_2Cl_2 (20 mL) were added into a solution of furfurylamine (380 mg, 3.9 mmol, 1.05 equiv.) in CH_2Cl_2 (5 mL) via a syringe under N_2 protection. The resulting solution was stirred for 5 h at room temperature and poured into 1.0 mM pH 7 phosphate buffer (30 mL). Afterwards, the crude product was purified by column chromatography on silica gel (hexane:EtOAc = 5:1) to give compound **N1-A3** (1.16 g, 95% yield) as a yellow-green oil. **^{1}H NMR (400 MHz, $CDCl_3$):** *δ* (ppm): 8.50 (d, 1H, *J* = 8.0 Hz), 8.25-8.20 (m, 2H), 7.54-7.45 (m, 2H), 7.16 (d, 1H, *J* = 8.0 Hz), 7.01 (dd, 1H, *J* = 2.0 Hz, 0.8 Hz), 6.02 (dd, 1H, *J* = 3.2 Hz, 2.0 Hz), 5.87 (dd, 1H, *J* = 3.2 Hz, 0.6Hz), 5.19 (m, 1H), 4.12 (d, 2H, *J* = 6.0 Hz), 2.87 (s, 6H). **^{13}C NMR (101 MHz, $CDCl_3$):** *δ* (ppm): 151.95, 149.41, 142.20, 134.72, 130.50, 129.84, 129.60, 128.42, 123.13, 118.61, 115.14, 110.14, 107.98, 45.42, 40.20. **ESI-MS** (*m/z*) for $C_{17}H_{18}N_2O_3S$ expected $[M+H]^+$: 331.1111, Found for $[M+H]^+$: 331.1061.

*Ethyl 4-((2-(methacryloyloxy)ethyl)amino)-4-oxobut-2-ynoate (**N1-B3**)*

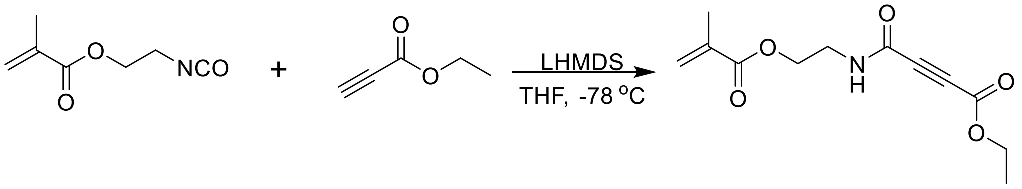

N1-B3

Ethylpropiolate (4.0 mL, 39.5 mmol, 1.0 equiv.) was dissolved in THF (60 mL) and the solution was cooled down to -78 °C with a dry ice/acetone bath. Then, LHMDS (1 M in THF, 40 mL, 40 mmol, 1.01 equiv.) was added with a syringe pump over a period of 30 min. The solution was stirred for an additional 30 min at -78 °C. Then, 2-isocyanatoethyl methacrylate (5.6 mL, 39.5 mmol, 1.0 equiv.) was added dropwise with a syringe pump. After the solution was stirred for 30 min at -78 °C, the reaction was terminated by adding a sat. aq. NH_4Cl solution (80 mL) at -78 °C. When the solution reached room temperature, H_2O (50 mL) was added and the two phases were separated. The aqueous layer was extracted with EtOAc (3×100 mL). The collected organic layers were washed with a sat. aq. $NaHCO_3$ and brine. Then, the organic layer was dried over $MgSO_4$, concentrated, and purified *via* column chromatography (hexane:EtOAc = 10:1) to give compound **N1-B3** (8.09 g, 81% yield) as a colourless oil. **^{1}H NMR (400 MHz, $CDCl_3$):** *δ* (ppm): 6.85 (t, *J* = 6.0 Hz, 1H), 6.10 (s, 1H), 5.61 – 5.56 (m, 1H), 4.28 – 4.20 (m, 4H), 3.61 (q, *J* = 5.6 Hz, 2H), 1.91 (s, 3H), 1.29 (t, *J* = 7.2 Hz, 3H). **^{13}C NMR (101 MHz, $CDCl_3$):** *δ* (ppm): 167.45, 152.34, 151.08, 135.77, 126.55, 76.93, 74.26, 63.03,

62.83, 39.28, 18.30, 13.94. **ESI-MS** (*m/z*) for $C_{12}H_{15}NO_5$ expected $[M+H]^+$: 254.1023, Found for $[M+H]^+$: 254.1030.

*Ethyl 1-(((5-(dimethylamino)naphthalene)-1-sulfonamido)methyl)-3-((2-(methacryloyloxy)ethyl)carbamoyl)-7-oxabicyclo[2.2.1]hepta-2,5-diene-2-carboxylate (**N1-DA1**) and Ethyl 4-(((5-(dimethylamino)naphthalene)-1-sulfonamido)methyl)-3-((2-(methacryloyloxy)ethyl)carbamoyl)-7-oxabicyclo[2.2.1]hepta-2,5-diene-2- carboxylate (**N1-DA2**)*

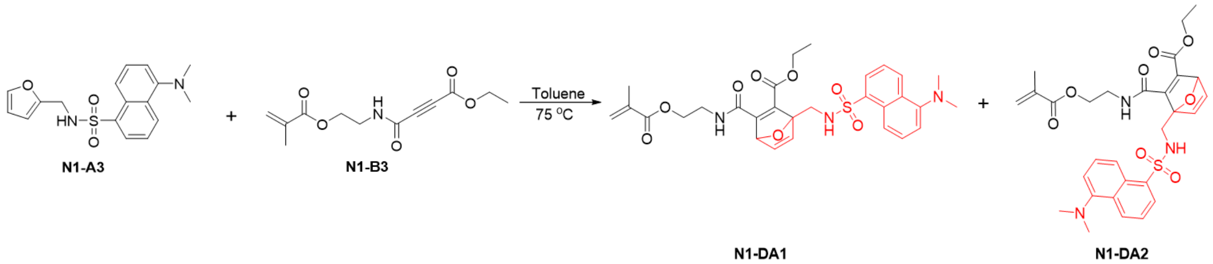

N1-A3 (0.66 g, 2.0 mmol, 1.01 equiv.) and **N1-B3** (0.5 g, 1.97 mmol, 1.0 equiv.) were dissolved in 3.5 mL dry toluene. The solution was stirred at 75 °C for 48 h. After the solvent was removed *in vacuo*, the crude product was purified by column chromatography on silica gel (hexane:EtOAc = 3:1) to give compound **N1-DA1** (0.22 g, 19% yield, as a yellow oil) and **N1-DA2** (0.37 g, 32% yield, as a yellow solid).

N1-DA1, **^{1}H NMR (400 MHz, $CDCl_3$):** *δ* (ppm): 8.93 (t, *J* = 5.6 Hz, 1H), 8.53 (d, *J* = 8.4 Hz, 1H), 8.32 – 8.24 (m, 2H), 7.57 – 7.48 (m, 2H), 7.17 (d, *J* = 7.6 Hz, 1H), 7.05 – 7.01 (dd, *J* = 5.2 Hz, 1.6 Hz, 1H), 6.94 (d, *J* = 5.2 Hz, 1H), 6.15 (s, 1H), 6.02 (t, *J* = 6.4 Hz, 1H), 5.56 – 5.53 (m, 1H), 5.50 (d, *J* = 2.0 Hz, 1H), 4.28 – 4.14 (m, 4H), 4.78 – 4.68 (m, 2H), 3.54 – 3.43 (m, 2H), 2.88 (s, 6H), 1.92 (s, 3H), 1.29 (t, *J* = 7.2 Hz, 3H). **^{13}C NMR (101 MHz, $CDCl_3$):** *δ* (ppm): 167.22, 164.17, 162.23, 159.57, 149.52, 144.03, 143.11, 136.07, 134.95, 130.34, 129.92, 129.74, 128.27, 126.14, 123.44, 115.31, 96.78, 83.57, 63.04, 62.28, 45.57, 42.72, 38.63, 18.33, 14.11. **ESI-MS** (*m/z*) for $C_{29}H_{33}N_3O_8S$ expected $[M+H]^+$: 584.2061, Found for $[M+H]^+$: 584.2000.

N1-DA2, **^{1}H NMR (400 MHz, $CDCl_3$):** *δ* (ppm): 8.86 (t, *J* = 5.6 Hz, 1H), 8.59 (d, *J* = 8.0 Hz, 1H), 8.28 – 8.21 (m, 2H), 7.58 – 7.50 (m, 2H), 7.22 – 7.14 (m, 2H), 6.74 (d, *J* = 5.2 Hz, 1H), 6.18 (s, 1H), 5.63 (d, *J* = 2.0 Hz, 1H), 5.59 (m, 1H), 5.09 (m, 1H), 4.23 (t, *J* = 5.2 Hz, 2H), 4.16 – 4.09 (qd, *J* = 7.2 Hz, 2.4 Hz, 2H), 3.95 – 3.87 (m, 1H), 3.65 – 3.58 (m, 2H), 3.53 – 3.46 (m, 1H), 2.90 (s, 6H), 1.95 (s, 3H), 1.27 (t, *J* = 7.2 Hz, 3H). **^{13}C NMR (101 MHz, $CDCl_3$):** *δ* (ppm): 167.27, 164.38, 162.10, 161.84, 146.18, 145.65, 141.57, 136.08, 134.24, 130.81, 129.84, 129.74, 128.79, 126.21, 123.34, 115.65, 95.72, 84.53, 63.25, 62.49, 45.60, 42.90, 38.61, 18.39, 13.89. **ESI-MS** (*m/z*) for $C_{29}H_{33}N_3O_8S$ expected $[M+H]^+$: 584.2061, Found for $[M+H]^+$: 584.2016.

We distinguished **N1-DA1** and **N1-DA2** by ^{1}H NMR (Supplementary Figure 62, 63). The chemical shifts of proton peak of H–N (**b**) in the two compounds show a characteristic difference. One is around 5.1 ppm, the other one is around 6.0 ppm. This is due to the fact that H–N (**b**) in **N1-DA1** forms hydrogen bonds with the oxygen atom belonging to the oxanorbornadiene structure. The formation of hydrogen bonds increased the electron density around the proton, causing the chemical shift of the proton to a high field. In contrast, the H–N (**b**) in **N1-DA2** cannot form hydrogen bonds with any oxygen atom.

*2-((2-Hydroxyethyl)disulfaneyl)ethyl methacrylate (**N2-A2**)*

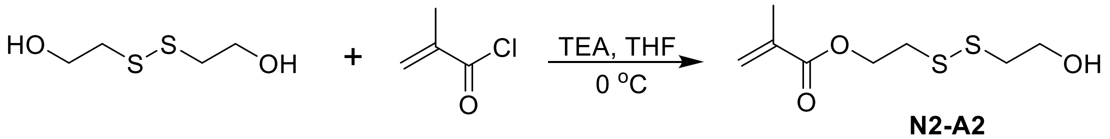

N2-A2

The preparation of **N2-A2** was carried out according to an adapted literature protocol[1]. 2-hydroxyethyl disulfide (2.0 g, 13 mmol, 1 equiv.) and TEA (3.0 mL, 21.5 mmol, 1.65 equiv.) were added in THF (30 mL) with a constant flow of N_2. The flask with the mixture was placed in an ice-water bath to keep the temperature at 0 °C. Afterwards, a solution of methacryloyl chloride (1.3 mL, 13.3 mmol, 1.02 equiv.) and 15 mL CH_2Cl_2 was added dropwise (15 mL/h) to the reaction mixture via an injection pump. The reaction mixture was stirred until complete consumption of the starting material, as indicated by TLC. Subsequently, the reaction mixture was washed three times with brine and three times with H_2O. Afterwards, the crude product was purified by column chromatography on silica gel (hexane:EtOAc = 5:1) to give compound **N2-A2** (2.2 g, 76% yield) as a colorless oil. **^{1}H NMR (400 MHz, $CDCl_3$):** *δ* (ppm): 6.12 (s, 1H), 5.55 (t, *J*=1.6 Hz, 1H), 4.41 (t, *J*=6.8 Hz, 2H), 3.87 (t, *J*=6.0 Hz, 2H), 2.96 (t, *J*=6.8 Hz, 2H), 2.87 (t, *J*=6.0 Hz, 2H), 2.21 (br, 1H), 1.93 (s, 3H). **^{13}C NMR (101 MHz, $CDCl_3$):** *δ* (ppm): 167.37, 136.09, 126.24, 62.73, 60.30, 41.74, 37.08, 18.38. **ESI-MS** (*m*/*z*) for $C_8H_{14}O_3S_2$ expected $[M+Na]^+$: 245.0277, Found for $[M+Na]^+$: 245.0242.

*2-((2-((((2-Oxo-2H-chromen-7-yl)oxy)carbonyl)oxy)ethyl)disulfaneyl)ethyl methacrylate (**N2-A3**)*

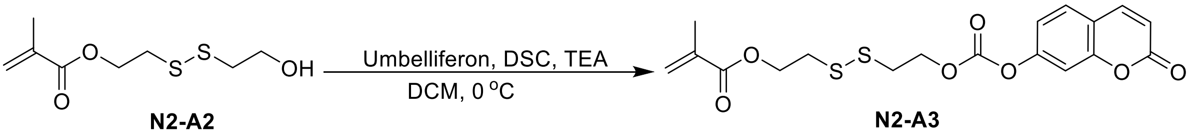

N2-A2 **N2-A3**

N,N'-disuccinimidyl carbonate (133 mg, 0.52 mmol, 1.0 equiv.) and Et_3N (90 µL, 0.65 µmol, 1.25 equiv.) were dissolved in 1.5 mL CH_2Cl_2. The mixture was cooled to 0 °C with an ice-water bath. Then, a solution of **N2-A2** (115 mg, 0.52 mmol, 1.0 equiv.) in 1 mL CH_2Cl_2 was added. The reaction mixture was allowed to stir in the ice-water bath for 1 h. Subsequently, a solution of umbelliferone (UMB, 84 mg, 0.52 mmol, 1 equiv.), Et_3N (90 µL, 0.65 mmol, 1.25 equiv.) and CH_2Cl_2/DMF (1.65 mL, v/v,10:1) was added into the reaction mixture. The reaction mixture was stirred for another 1 h at 0 °C. Then, the reaction mixture was allowed to warm up slowly to room temperature for further reaction. The reaction mixture was stirred until complete consumption of the starting material, as indicated by TLC. Afterwards, the solvent was removed *in vacuo*. The residue was re-dissolved in CH_2Cl_2, and washed with H_2O (3×20 mL). The mixture was then purified by silica column chromatography (hexane:EtOAc = 4:1) to give compound **N2-A3** (128 mg, 60% yield) as a colorless oil. **^{1}H NMR (400 MHz, $CDCl_3$):** *δ* (ppm): 7.68 (d, *J*=9.6 Hz, 1H), 7.48 (d, *J*=8.4 Hz, 1H), 7.19 (d, *J*=2.4 Hz, 1H), 7.11-7.14 (dd, *J*=2.4, 8.4 Hz, 1H), 6.37 (d, *J*=9.6 Hz, 1H), 6.11 (s, 1H), 5.57 (t, *J*=1.6 Hz, 1H), 4.51 (t, *J*=6.8 Hz, 2H), 4.40 (t, *J*=6.8 Hz, 2H), 2.97-3.03 (m, 4H), 1.92 (s, 3H). **^{13}C NMR (101 MHz, $CDCl_3$):** *δ* (ppm): 167.13, 160.20, 154.65, 153.25, 152.61, 142.80, 136.00, 128.79, 126.13, 117.69, 116.92, 116.31, 109.88, 66.73, 62.50, 37.25, 36.85, 18.32. **ESI-MS** (*m*/*z*) for $C_{18}H_{18}O_7S_2$ expected $[M+H]^+$: 411.0567, Found for $[M+H]^+$: 411.0531.

*2-((2-((((4-Ethyl-3,14-dioxo-3,4,12,14-tetrahydro-1H-pyrano[3',4':6,7]indolizino[1,2-b]quinolin-4-yl)oxy)carbonyl)oxy)ethyl)disulfaneyl)ethyl methacrylate (**N2-B3**)*

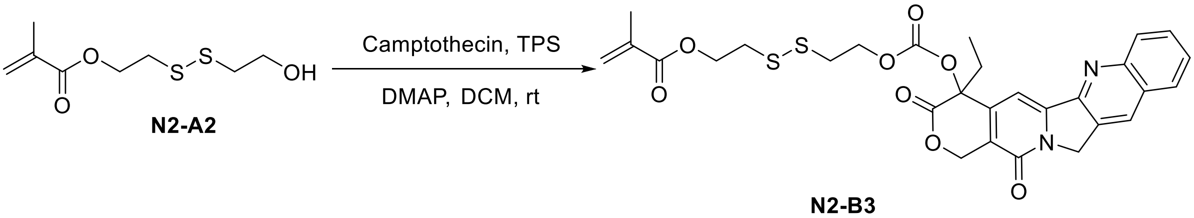

The synthesis of **N2-B3** was performed using an adapted literature protocol[2]. Camptothecin (CPT, 200 mg, 0.57 mmol) was dissolved in 12 mL anhydrous CH_2Cl_2. Then, triphosgene (TPS, 70 mg, 0.23 mmol) was added under stirring. After 5 min stirring, DMAP (224 mg, 1.83 mmol) in 0.5 mL CH_2Cl_2 was added into the mixture and stirred for another 1 h at room temperature. Afterwards, a solution of **N2-A2** (378mg, 1.7 mmol) in 1 mL CH_2Cl_2 was added to the reaction mixture for further reaction overnight. Subsequently, the excess phosgene (CAUTION: TOXIC) was removed by Ar purging and neutralized by bubbling the exhaust gas through a 2 M NaOH solution. Afterwards, the reaction mixture was washed three times with H_2O. Then, the crude product was purified by column chromatography on silica gel (hexane:EtOAc = 1:1) to give compound **N2-B3** (180 mg, 53% yield) as a white solid. **^{1}H NMR (400 MHz, $CDCl_3$):** *δ* (ppm): 8.39 (s, 1H), 8.22 (d, *J*=8.4 Hz, 1H), 7.93 (d, *J*=8.0 Hz, 1H), 7.83 (m, 1H), 7.66 (m, 1H), 7.34 (s, 1H), 6.07 (s, 1H), 5.69 (d, *J*=17.2 Hz, 1H), 5.54 (t, *J*=1.6 Hz, 1H), 5.38 (d, *J*=17.2 Hz, 1H), 5.28 (s, 2H), 4.31-4.39 (m, 4H), 2.92 (q, *J*=6.0 Hz, 4H), 2.32-2.23 (m, 1H), 2.19-2.10 (m, 1H), 1.89 (s, 3H), 1.00 (t, *J*=7.6 Hz, 3H). **^{13}C NMR (101 MHz, $CDCl_3$):** *δ* (ppm): 167.37, 167.15, 157.39, 153.57, 152.37, 148.94, 146.56, 145.71, 136.06, 131.35, 130.88, 129.74, 128.59, 128.32, 128.30, 128.23, 126.10, 120.40, 96.13, 78.14, 67.19, 66.65, 62.52, 50.12, 37.35, 36.67, 32.00, 18.36, 7.75. **ESI-MS:** (*m/z*) for $C_{29}H_{28}N_2O_8S_2$ expected $[M+H]^+$: 597.1360, Found for $[M+H]^+$: 597.1328.

*1-(5-(Hydroxymethyl)furan-2-yl)ethan-1-ol (**N3-A2**)*

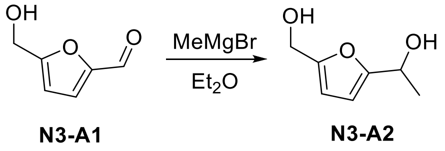

N3-A1 (1.14 g, 9.0 mmol) and diethyl ether (45 mL) were added into a round bottom flask. The solution was cooled to -30 °C, followed by the dropwise addition of MeMgBr (3 M in Et_2O, 7.0 mL, 21.0 mmol). The mixture was allowed to warm to room temperature and stirred for another 12 h, after that the reaction was cooled to 0 °C and terminated with 10% NH_4Cl (30 mL). The reaction mixture was extracted with EtOAc (3×100 mL) and the combined organic phase was dried over $MgSO_4$, filtered, and concentrated under reduced pressure. Then, the crude product was purified by column chromatography on silica gel (CH_2Cl_2:MeOH = 100:5) to give compound **N3-A2** as a viscous yellow oil (1.18 g, 92%). **^{1}H NMR (400 MHz, $(CD_3)_2SO$):** *δ* (ppm): 6.16 (d, *J*=3.2 Hz, 1H), 6.12 (d, *J*=3.2 Hz, 1H), 5.21 (d, *J*=5.2 Hz, 1H), 5.16 (t, *J*=5.6 Hz, 1H), 4.68-4.60 (m, 1H), 4.34 (d, *J*=5.6 Hz, 2H), 1.34 (d, *J*=6.4 Hz, 3H). **^{13}C NMR (101 MHz, $(CD_3)_2SO$):** *δ* (ppm): 158.24, 154.06, 107.34, 105.10, 61.89, 55.77, 22.09.

*(5-(1-Hydroxyethyl)furan-2-yl)methyl methacrylate (**N3-A3**)*

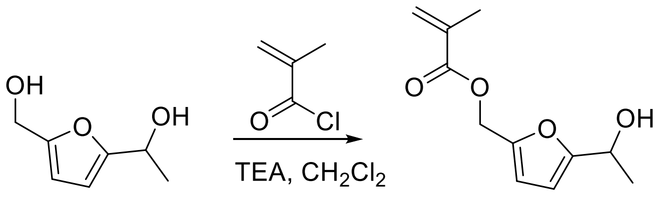

N3-A2 **N3-A3**

N3-A2 (0.45 g, 3.17 mmol), Et_3N (0.33 mL, 4.7 mmol), and CH_2Cl_2 (30 mL) were added into a three neck flask with a constant flow of N_2. The flask with the mixture was placed in an ice-water bath to keep the temperature at 0 °C. Afterwards, a solution of methacryloyl chloride (0.36 mL, 3.7 mmol, 1.17 equiv.) and 8 mL CH_2Cl_2 was added dropwise (10 mL/h) to the reaction mixture via an injection pump. The reaction mixture was stirred under nitrogen for another 30 min and was allowed to warm slowly to room temperature. After 15 h, the reaction mixture was extracted with EtOAc (3×20 mL) and the combined organic phase was dried over $MgSO_4$, filtered, and concentrated under reduced pressure. Then, the crude product was purified by column chromatography on silica gel (hexane:EtOAc = 6:1) to give compound **N3-A3** (0.64 g, 96% yield) as a colorless oil. **^{1}H NMR (400 MHz, $CDCl_3$):** *δ* (ppm): 6.34 (d, *J*=3.2 Hz, 1H), 6.18 (d, *J*=3.2 Hz, 1H), 6.11 (s, 1H), 5.58-5.54 (m, 1H), 5.08 (s, 2H), 4.85 (q, *J*=6.4 Hz, 1H), 2.26 (s, 1H), 1.92 (s, 3H), 1.52 (d, *J*=6.4 Hz, 3H). **^{13}C NMR (101 MHz, $CDCl_3$):** *δ* (ppm): 167.21, 158.51, 148.98, 136.08, 126.24, 111.41, 106.15, 63.67, 58.52, 21.27, 18.40. **ESI-MS:** (*m/z*) for $C_{11}H_{14}O_4$ expected $[M+Na]^+$: 233.0972, found for $[M+H]^+$: 233.0784.

*2-(2,5-Dioxo-2,5-dihydro-1H-pyrrol-1-yl)ethyl methacrylate (**N3-B2**)*

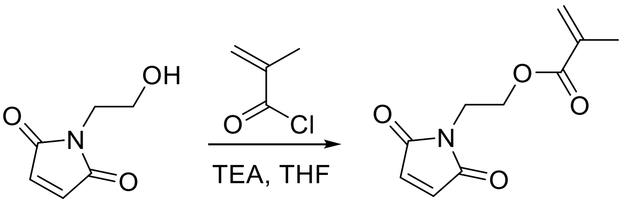

N3-B1 **N3-B2**

N3-B1 (1.0 g, 7.1 mmol, 1.0 equiv.) and Et_3N (1,520 mL, 10.9 mmol, 1.5 equiv.) were dissolved in 50 mL THF. The mixture was cooled to 0 °C with an ice-water bath. Then, a solution of methacryloyl chloride (1.05 g, 10 mmol, 1.4 equiv.) in 1.5 mL THF was added slowly into the mixture. The reaction mixture was stirred further in the ice-water bath for 2 h. Then, the reaction mixture was allowed to warm up slowly to room temperature. The reaction mixture was stirred until complete consumption of the starting material, as indicated by TLC. Afterwards, the solvent was removed *in vacuo*. The residue was re-dissolved in EtOAc, and washed with H_2O. After condensation under reduced pressure, the mixture was purified by silica column chromatography (hexane:EtOAc = 3:1) to give compound **N3-B2** (1.21 g, 82% yield) as a white solid. **^{1}H NMR (400 MHz, $CDCl_3$):** *δ* (ppm): 6.71 (s, 1H), 6.04 (s, 1H), 5.56-5.53 (m, 1H), 4.27 (t, *J*=5.2 Hz, 2H), 3.83 (t, *J*=5.2 Hz, 2H), 1.88 (s, 3H). **^{13}C NMR (101 MHz, $CDCl_3$):** *δ* (ppm): 170.48, 167.14, 135.85, 134.34, 126.35, 61.83, 36.90, 18.31. **ESI-MS:** (*m/z*) for $C_{10}H_{11}NO_4$ expected $[M+Na]^+$: 232.0581, Found for $[M+H]^+$: 232.0580.

*(5-(1-(((4-(Pyren-1-yl)butoxy)carbonyl)oxy)ethyl)furan-2-yl)methyl methacrylate (**N3-A4**)*

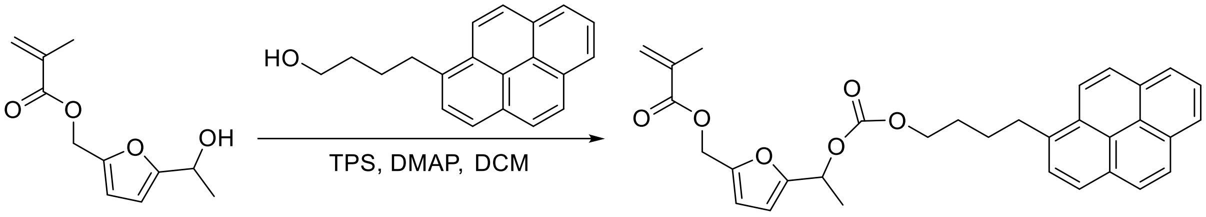

N3-A3 **N3-A4**

N3-A3 (140 mg, 0.66 mmol) was dissolved in 10 mL anhydrous CH_2Cl_2. Then, triphosgene (TPS, 94 mg, 0.31 mmol) was added under stirring. After 5 min stirring, DMAP (224 mg, 1.83 mmol) was added into the mixture and stirred for another 1 h at room temperature. Afterwards, a solution of 1-pyrenebutanol (300 mg, 1.09 mmol) in 2 mL CH_2Cl_2 was added to the reaction mixture and reacted overnight. Subsequently, the excess phosgene (CAUTION: TOXIC) was removed by Ar purging and neutralized by bubbling the exhaust gas through a 2 M NaOH solution. Afterwards, the reaction mixture was washed three times with H_2O. After evaporation of the solvent under reduced pressure, the crude product was purified by column chromatography on silica gel (hexane:EtOAc = 10:1) to give compound **N3-A4** (68 mg, 43% yield) as a white solid. **^{1}H NMR (400 MHz, $CDCl_3$):** δ (ppm): 8.27 (d, J=9.2 Hz, 1H), 8.18-7.96 (m, 7H), 7.85 (d, J=7.6 Hz, 1H), 6.34 (d, J=3.2 Hz, 1H), 6.20 (d, J=3.2 Hz, 1H), 6.08 (s, 1H), 5.50-5.47 (m, 1H), 5.09 (s, 2H), 4.44 (q, J=6.8 Hz, 1H), 3.54-3.42 (m, 2H), 3.34 (t, J=8.0 Hz, 2H), 1.96-1.87 (m, 5H), 1.79-1.70 (m, 2H), 1.51 (d, J=6.4 Hz, 3H). **^{13}C NMR (101 MHz, $CDCl_3$):** δ (ppm): 167.14, 156.94, 149.05, 137.01, 136.11, 131.56, 131.04, 129.88, 128.74, 127.64, 127.41, 127.25, 126.66, 126.09, 125.90, 125.20, 125.16, 124.93, 124.89, 124.76, 123.63, 111.19, 107.60, 70.85, 68.62, 58.60, 33.37, 29.95, 28.54, 19.90, 18.38.

*(2-(2-(Methacryloyloxy)ethyl)-1,3-dioxo-7-(1-(((4-(pyren-1-yl)butoxy)carbonyl)oxy)ethyl)-1,2,3,3a,7,7a-hexahydro-4H-4,7-epoxyisoindol-4-yl)methyl methacrylate (**N3-A5**)*

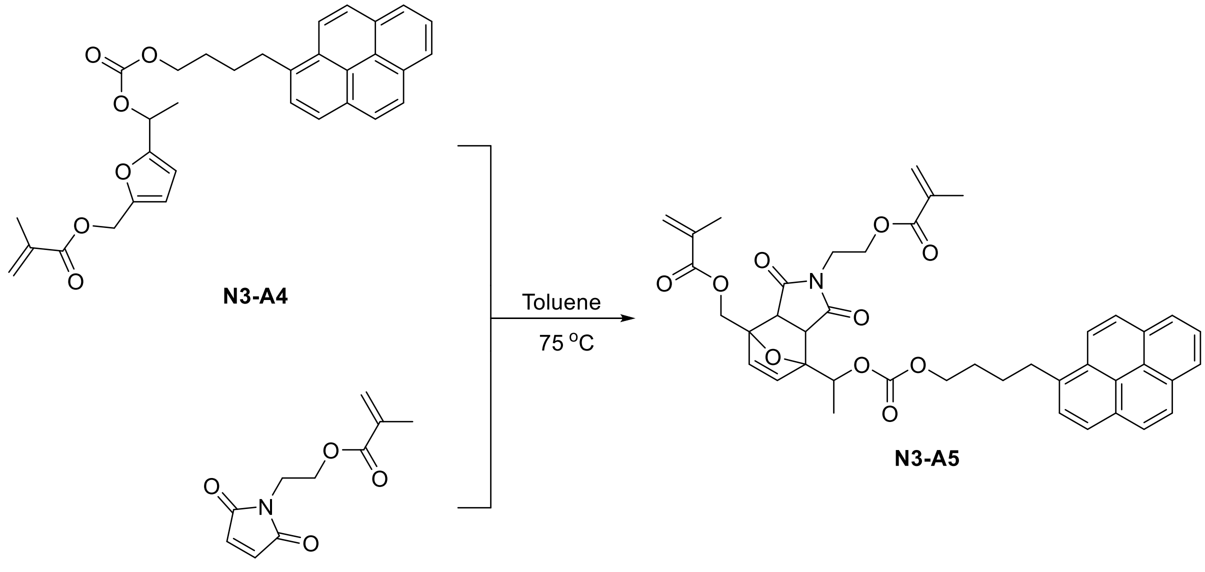

N3-B2

N3-A4 (60 mg, 0.11 mmol, 1.0 equiv.) and **N3-B2** (42 mg, 0.2 mmol, 1.8 equiv.) were dissolved in toluene (1.1 mL) and stirred for 48 h at 75 °C. Afterwards, the solvent was removed *in vacuo*. The residue was re-dissolved in EtOAc, and washed with H_2O. After removal of the solvent

under reduced pressure, the crude reaction mixture was purified by column chromatography (hexane:EtOAc = 1:1) to give compound **N3-A5** (35 mg, 44% yield) as a yellow oil. **^{1}H NMR (400 MHz, $CDCl_3$):** δ (ppm): 8.31 (d, *J*=9.2 Hz, 1H), 8.15 (d, *J*=7.6 Hz, 2H), 8.12-8.06 (m, 2H), 8.04-7.95 (m, 3H), 7.88 (d, *J*=8.0 Hz, 1H), 6.46 (d, *J*=5.6 Hz, 1H), 6.29 (d, *J*=5.6 Hz, 1H), 6.10 (s, 1H), 6.03 (s, 1H), 5.58 (t, *J*=1.6 Hz, 1H), 5.48 (t, *J*=1.6 Hz, 1H), 4.88 (d, *J*=12.8 Hz, 1H), 4.41 (d, *J*=12.8 Hz, 1H), 4.33-4.20 (m, 2H), 3.89 (q, *J*=6.0 Hz, 1H), 3.78-3.67 (m, 4H), 3.40 (t, *J*=7.6 Hz, 2H), 2.92 (d, *J*=6.4 Hz, 1H), 2.82 (d, *J*=6.4 Hz, 1H), 2.04-1.95 (m, 2H), 1.94 (s, 3H), 1.85 (s, 3H), 1.83-1.76 (m, 2H), 1.13 (d, *J*=6.4 Hz, 3H). **^{13}C NMR (101 MHz, $CDCl_3$):** δ (ppm): 174.01, 173.97, 167.05, 166.87, 138.10, 137.25, 137.20, 136.00, 135.96, 131.55, 131.04, 129.87, 128.78, 127.66, 127.58, 127.20, 126.66, 126.25, 126.21, 125.95, 125.22, 125.16, 124.97, 124.91, 124.76, 123.73, 93.29, 89.44, 71.57, 68.94, 61.72, 60.82, 51.45, 50.25, 37.90, 33.55, 30.27, 28.58, 18.40, 18.31, 15.97.

*Bis(6-hydroxyhexyl) but-2-ynedioate (**N4-A2**)*

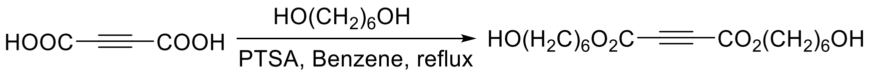

N4-A1 **N4-A2**

Acetylenedicarboxylic acid (2.0 g, 17.5 mmol), 1,6-hexanediol (20.8 g, 176 mmol) and *p*-toluenesulfonic acid monohydrate (333.2 mg, 1.75 mmol) were dissolved in 28 mL benzene in a 100 mL round bottom flask. Then, the reaction flask was equipped with a reflux condenser fitted with a Dean-Stark trap. The reaction mixture was heated up to 100 °C. After 24 h, ca. 15 mL of solution had collected in the trap. The reaction mixture was allowed to cool down to room temperature. Then, the reaction solution was diluted with Et_2O (30 mL) and cooled to -10 °C for 1 h. The solution was decanted and the solids were rinsed with additional portions of Et_2O. The combined organic layer was washed with saturated $NaHCO_3$ (2×20 mL), water (3×20 mL) and brine (20 mL). Afterwards, the solvent was removed *in vacuo*. The crude product was purified by column chromatography (CH_2Cl_2:MeOH = 15:1) to give compound **N4-A2** (3.7 g, 67% yield) as a pale-yellow oil. **^{1}H NMR (400 MHz, $CDCl_3$):** δ (ppm): 4.24 (t, *J*=6.4 Hz, 4H), 3.63 (t, *J*=6.4 Hz, 4H), 2.08 (s, 2H), 1.75-1.67 (m, 4H), 1.62-1.54 (m, 4H), 1.45-1.36 (m, 8H). **^{13}C NMR (101 MHz, $CDCl_3$):** δ (ppm): 151.99, 74.75, 67.04, 62.62, 32.49, 28.27, 25.57, 25.34. **ESI-MS:** (*m/z*) for $C_{16}H_{26}O_6$ expected $[M+H]^+$: 315.1802, Found for $[M+H]^+$: 315.1951.

*Bis(6-hydroxyhexyl) 1-(((5-(dimethylamino)naphthalene)-1-sulfonamido)methyl)-7-oxabicyclo[2.2.1]hepta-2,5-diene-2,3-dicarboxylate (**N4-A3**)*

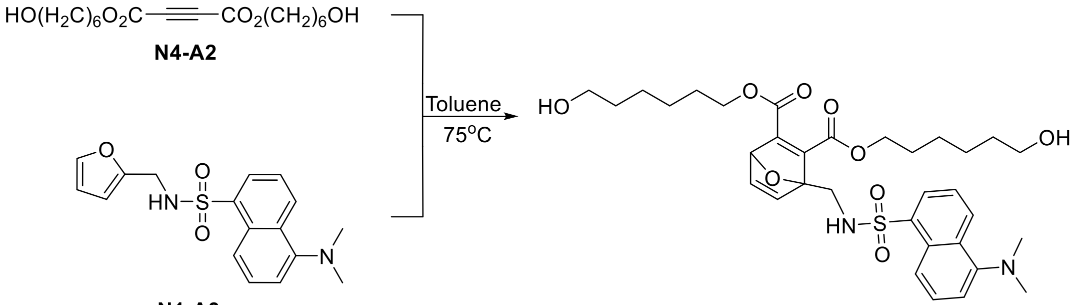

N1-A3 **N4-A3**

N4-A2 (2.0 g, 6.37 mmol, 1.0 equiv.) and compound **N1-A3** (3.16 g, 9.55 mmol, 1.5 equiv.) were dissolved in 10 mL toluene and stirred at 75 °C in a 50 mL round bottom flask. After 36 h, the reaction solution was cooled down to room temperature. Afterwards, the solvent was removed *in vacuo*. The residue was re-dissolved in EtOAc (50 mL), and washed with H_2O (30

mL). The organic layer was removed with a rotary evaporator. Then, the crude product mixture was purified by column chromatography (hexane:EtOAc = 1:5) to give compound **N4-A3** (2.3 g, 56% yield) as an orange oil. **^{1}H NMR (400 MHz, CDCl$_3$):** *δ* (ppm): 8.53 (d, *J*=8.4 Hz, 1H), 8.26-8.20 (m, 2H), 7.52 (q, *J*=8.8 Hz, 2H), 7.16 (d, *J*=7.6 Hz, 1H), 7.10 (dd, *J*=5.2 Hz 2.0 Hz, 1H), 6.86 (d, *J*=5.2 Hz, 1H), 5.54 (d, *J*=2.0 Hz, 1H), 5.46 (t, *J*=6.0 Hz, 1H), 4.19-4.05 (m, 4H), 3.70 (dd, *J*=13.6 Hz 7.2 Hz, 1H), 3.62-3.52 (m, 5H), 2.86 (s, 6H), 2.16 (s, 2H), 1.68-1.59 (m, 4H), 1.58-1.48 (m, 4H), 1.40-1.30 (m, 8H). **^{13}C NMR (101 MHz, CDCl$_3$):** *δ* (ppm): 163.69, 162.67, 153.19, 152.03, 151.73, 144.89, 142.73, 134.38, 130.70, 129.93, 129.69, 129.63, 128.62, 123.25, 118.68, 115.37, 95.91, 84.01, 65.91, 65.66, 62.58, 45.48, 41.98, 32.51, 28.48, 28.39, 25.66, 25.65, 25.44, 25.40. **ESI-MS:** (*m/z*) for $C_{33}H_{44}N_2O_9S$ expected [M+H]$^+$: 645.2840, Found for [M+H]$^+$: 645.3102.

*Bis(6-(methacryloyloxy)hexyl) 1-(((5-(dimethylamino)naphthalene)-1-sulfonamido)methyl)-7-oxabicyclo[2.2.1]-hepta-2,5-diene-2,3-dicarboxylate (**N4-A4**)*

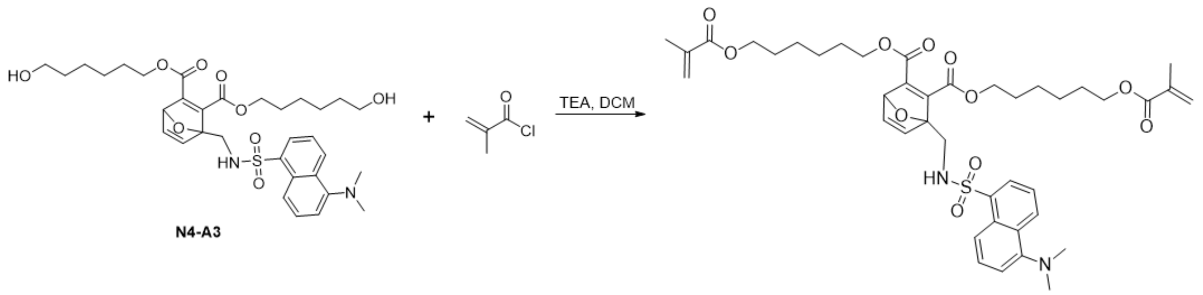

N4-A3 (1.74 g, 2.7 mmol, 1.0 equiv.) and Et_3N (964 uL, 6.9 mmol, 2.5 equiv.) were dissolved in 30 mL CH_2Cl_2. The mixture was cooled to 0 °C with an ice-water bath. Then, a solution of methacryloyl chloride (653 µL, 6.75 mmol, 2.5 equiv.) in 6 mL CH_2Cl_2 was added slowly into the mixture. The reaction mixture was stirred in the ice-water bath for 1 h. Then, the reaction mixture was allowed to warm up to room temperature to react further. The reaction mixture was stirred until complete consumption of the starting material, as indicated by TLC. Afterwards, 10 mL water was added into the reaction solution to hydrolyze the unreacted methacryloyl chloride. The organic solvent was collected and removed *in vacuo*. The crude product was then purified by silica column chromatography (hexane:EtOAc = 1:1) to give compound **N4-A4** (1.26 g, 60% yield) as a light yellow oil. **^{1}H NMR (400 MHz, CDCl$_3$):** *δ* (ppm): 8.53 (d, *J*=8.4 Hz, 1H), 8.26-8.20 (m, 2H), 7.57-7.48 (m, 2H), 7.17 (d, *J*=7.6 Hz, 1H), 7.10 (dd, *J*=5.2 Hz 2.0 Hz, 1H), 6.87 (d, *J*=5.2 Hz, 1H), 6.08 (s, 2H), 5.56-5.51 (m, 3H), 5.29-5.23 (m, 1H), 4.18-4.07 (m, 8H), 3.70 (dd, *J*=13.6 Hz 6.8 Hz, 1H), 3.57 (dd, *J*=13.6 Hz 5.6 Hz, 1H), 2.87 (s, 6H), 1.92 (s, 6H), 1.71-1.61 (m, 8H), 1.43-1.34 (m, 8H). **^{13}C NMR (101 MHz, CDCl$_3$):** *δ* (ppm): 167.58, 167.55, 163.54, 162.50, 153.47, 152.09, 151.82, 144.90, 142.79, 136.54, 136.51, 134.40, 130.73, 129.98, 129.74, 129.66, 128.66, 125.39, 125.37, 123.24, 118.67, 115.38, 95.84, 84.04, 65.81, 65.50, 64.65, 64.57, 45.50, 42.05, 28.56, 28.46, 28.36, 25.73, 25.70, 25.57, 18.42. **ESI-MS:** (*m/z*) for $C_{41}H_{52}N_2O_{11}S$ expected [M+H]$^+$: 781.3365, Found for [M+H]$^+$: 781.3690.

*2-(6-Hydroxyhexyl) 3-(6-(methacryloyloxy)hexyl) 1-(((5-(dimethylamino) naphthalene)-1-sulfonamido)methyl)-7-oxabicyclo[2.2.1]hepta-2,5-diene-2,3-dicarboxylate (**N4-B4**)*

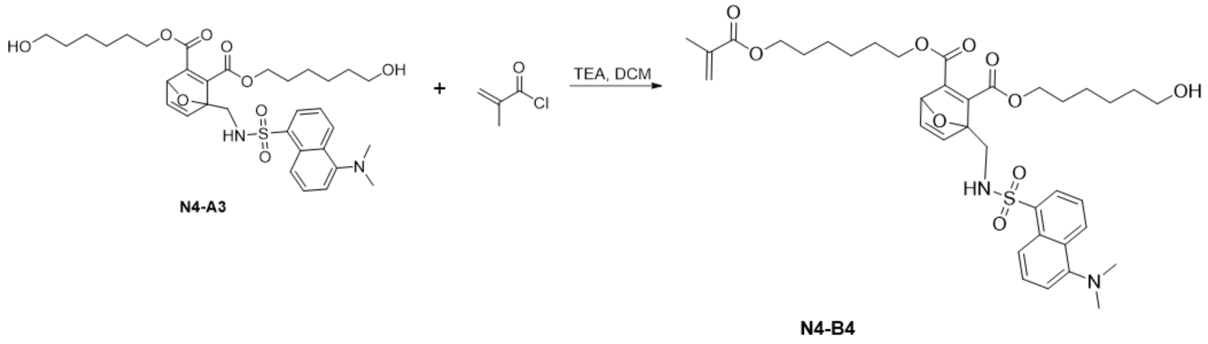

N4-A3 (0.64 g, 1.0 mmol, 1.0 equiv.) and Et_3N (154 uL, 1.1 mmol, 1.1 equiv.) were dissolved in 20 mL CH_2Cl_2. The mixture was cooled to 0 °C with an ice-water bath. Then, a solution of methacryloyl chloride (102 µL, 1.05 mmol, 1.05 equiv.) in 2 mL CH_2Cl_2 was added slowly into the mixture. The reaction mixture was stirred in the ice-water bath for 1 h. Then, the reaction mixture was allowed to warm up to room temperature to react further. The reaction mixture was stirred until complete consumption of the starting material, as indicated by TLC. Afterwards, 3 mL water was added into the reaction solution to hydrolyze the unreacted methacryloyl chloride. Then, the organic phase was collected and removed *in vacuo*. The crude product was then purified by silica column chromatography (hexane:EtOAc = 1:2) to give compound **N4-B4** (0.28 g, 39% yield) as a light yellow oil. **^{1}H NMR (400 MHz, $CDCl_3$):** δ (ppm): 8.54 (d, J=8.4 Hz, 1H), 8.27-8.20 (m, 2H), 7.57-7.49 (m, 2H), 7.17 (d, J=7.6 Hz, 1H), 7.11 (d, J=5.2 Hz 2.0 Hz, 1H), 6.87 (d, J=5.2 Hz, 1H), 6.08 (s, 1H), 5.56-5.51 (m, 2H), 5.27 (t, J=6.0 Hz, 1H), 4.19-4.08 (m, 6H), 3.70 (dd, J=13.6 Hz 6.8 Hz, 1H), 3.65-3.53 (m, 3H), 2.87 (d, J=4.4 Hz, 6H), 1.93 (s, 3H), 1.73-1.60 (m, 8H), 1.42-1.33 (m, 8H). **^{13}C NMR (101 MHz, $CDCl_3$):** δ (ppm): 167.66, 163.62, 162.59, 153.45, 152.10, 151.85, 144.93, 142.78, 136.53, 134.39, 130.76, 129.98, 129.79, 129.66, 128.69, 125.46, 123.25, 118.67, 115.39, 95.86, 84.05, 65.88, 65.62, 64.69, 62.72, 45.51, 42.05, 32.59, 28.57, 28.46, 28.37, 25.72, 25.57, 25.46, 25.41, 18.44.

*Bis(6-((2-bromo-2-methylpropanoyl)oxy)hexyl) 1-(((5-(dimethylamino) naphthalene)-1-sulfonamido)methyl)-7-oxabicyclo[2.2.1]hepta-2,5-diene-2,3-dicarboxylate (**N4-C4**)*

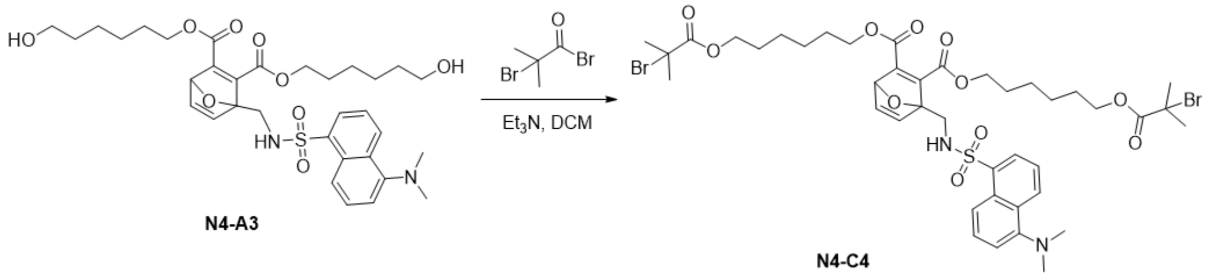

N4-A3 (0.13 g, 0.2 mmol, 1.0 equiv.) and Et_3N (69 µL, 0.5 mmol, 2.5 equiv.) were dissolved in 6 mL CH_2Cl_2. The mixture was cooled to 0 °C with an ice-water bath. Then, a solution of α-bromoisobutyryl bromide (102 mg, 0.44 mmol, 2.2 equiv.) was added dropwise into the mixture. The reaction mixture was stirred in the ice-water bath for 30 min. Then, the reaction mixture was allowed to warm up to room temperature to react further. The reaction mixture was stirred until complete consumption of the starting material, as indicated by TLC. Then, the reaction mixture was collected and solvent was removed *in vacuo*. The crude product was then purified

by silica column chromatography (hexane:EtOAc = 2:1) to give compound **N4-C4** (0.16 g, 85% yield) as a yellow oil. **^{1}H NMR (400 MHz, $CDCl_3$):** *δ* (ppm): 8.57 (d, *J*=8.0 Hz, 1H), 8.25 (d, *J*=7.6 Hz, 2H), 7.54 (q, *J*=8.0 Hz, 2H), 7.19 (d, *J*=7.6 Hz, 1H), 7.11 (dd, *J*=5.2 Hz 2.0 Hz, 1H), 6.88 (d, *J*=5.2 Hz, 1H), 5.54 (d, *J*=1.6 Hz, 1H), 5.23 (t, *J*=6.0 Hz, 1H), 4.19-4.07 (m, 8H), 3.71 (dd, *J*=13.6 Hz 6.8 Hz, 1H), 3.56 (dd, *J*=13.6 Hz 5.6 Hz, 1H), 2.89 (s, 6H), 1.92 (s, 12H), 1.73-1.61 (m, 8H), 1.45-1.34 (m, 8H). **^{13}C NMR (101 MHz, $CDCl_3$):** *δ* (ppm): 171.78, 163.54, 162.49, 153.52, 151.81, 144.95, 142.79, 134.45, 130.69, 129.81, 129.67, 128.65, 123.40, 115.51, 95.83, 84.06, 65.97, 65.87, 65.80, 65.49, 56.13, 56.07, 45.59, 42.08, 30.87, 30.85, 28.45, 28.35, 28.30, 25.52. **ESI-MS:** (*m/z*) for $C_{41}H_{54}Br_2N_2O_{11}S$ expected $[M+H]^+$: 941.1888, Found for $[M+H]^+$: 941.2257.

2.3. Preparation of linear polymers

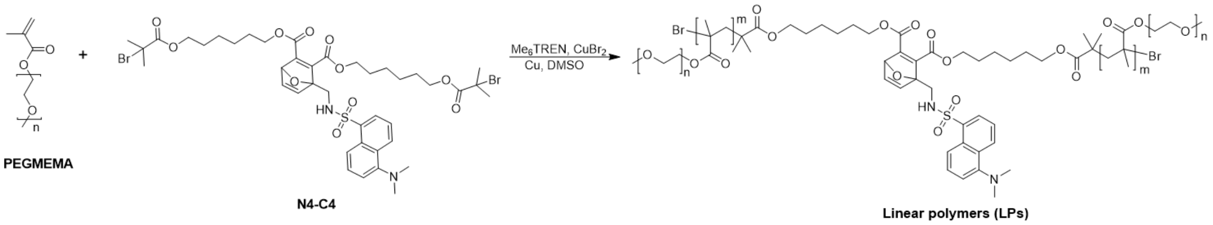

LPs were synthesized as follows. PEGMEMA (8 g, 26.6 mmol, 1330 equiv.), **N4-C4** (20 mg, 0.02 mmol, 1.0 equiv.), Me_6TREN (0.55 mg, 2.4 μmol), and $CuBr_2$ (0.29 mg, 1.3 μmol) were dissolved in DMSO (6 mL) in a Schlenk flask and sealed with a rubber septum reinforced with a cable tie. The solution was degassed by 3 consecutive freeze-pump-thaw cycles. During that time, copper wire (6 cm) was activated in 37% HCl for 30 min, subsequently washed with H_2O and acetone, and then dried. The copper wire was added to the reaction solution and then polymerization was allowed to proceed for 11 h at room temperature. The viscous solution was diluted with THF to terminate the polymerization by passing through a plug of basic Al_2O_3. After concentration *in vacuo*, the mixture was added dropwise to ice-cold Et_2O under stirring. Et_2O was decanted and the viscous polymer redissolved in THF. Afterwards, it was again precipitated in fresh Et_2O. Linear polymers with molar masses of 140 kDa were obtained after repeating the precipitation process for three times.

3. Preparation of polymeric microbubbles and microgels

3.1. Preparation of PMBs with masked dansyl-fluorophore (PMB-MDF)

High-purity N_2 was used as the gas source to prepare the G/O/W double-emulsion polymeric microbubbles. First, N_2 was blown into the microfluidic channel with a flow controller (digital pressure valve) to regulate the flow rate. Then, the oil phase solution was loaded into a glass syringe (Hamilton-Gastight #1001), which was further injected into the channel by a syringe pump (PHD ULTRA™, Harvard Apparatus). The continuous phase solution was squeezed into the microfluidic device through a pressure pump (Millipore) with the flow controller. Once the flow rate of each phase reaches the desired value, the double emulsion production in the microfluidic device proceeded automatically. PMB products was collected in a glass vial. Finally, PMBs were washed for 3× using DI H_2O before sonication. G/O/W emulsion: Gas: N_2; Oil phase: PPGDA (400 mg), PPG (50 mg), TPO-L (10 mg), toluene (440 mg), **N1-CL** (100 mg), **N1-DA2** (2 mg); H_2O phase: PVA (4.5%, w/w), glycerol (40%, v/v). Flow rate: gas (530 mbar), oil phase (90 nL min^{-1}), H_2O phase (2.3 bar). A high-power UV LED LZ1-10UV0R-0000 (set to 200 mA, Osram) was fixated above the outlet tube to initiate the polymerization of oil-phase shell of PMBs. Next, 100 µL PMBs was mixed with 1 mL MeCN:H_2O (1:1, v/v), then was transferred to a 1.5 mL Eppendorf tube or 24 well plate (lumox® multiwall 24, SARSTEDT) for sonication.

Quantification of molecular release: 100 µL PMB-MDF suspension was dispersed in 1 mL MeCN/H_2O (1:1, v/v) solution, then was transferred to a 1.5 mL Eppendorf tube for 30 min sonication (20 kHz). Then, 3 mg MCE (1000× excess to **N1-DA2**) was added into the mixture and reacted with covalently loaded **N1-DA2** in the fragments. 3 d incubation (Eppendorf ThermoMixer® C, 700 rpm) at 37 ^{o}C, leading to the occurrence of complete reaction of **N1-DA2** and MCE. After centrifugation at RCF 8000 ×g for 15 min, fragments was removed through a centrifugal filter (3,000 Da MWCO) by centrifugation at 5,000 rpm. The fluorescence intensity of collected solution was measured at 545 nm, the value of fluorescence intensity as F_m.

Subsequently, we measured the fluorescence intensity of sonicated PMBs (no MCE added) by the same method. Fluorescence intensity at 545 nm of sonicated suspension was collected as F_s. F_m and F_s were used to calculate the percentage of molecular release based on the standard (fluorescence intensity-concentration) curve of fluorophore.

3.2. Preparation of PMB-UMB or PMB-CPT

The preparation of PMBs-UMB or PMBs-CPT is similar

Production of G/O/W double emulsion: Gas: N_2; Oil phase: PPGDA (400 mg), PPG (50 mg), TPO-L (10 mg), Toluene (440 mg), **N1-CL** (100 mg), **N2-A3** or **N2-B3** (2 mg); H_2O phase: PVA (4.5%, w/w), glycerol (40%, v/v). Flow rate: gas (530 mbar), oil phase (90 nL min^{-1}), H_2O phase (2.3 bar). A high-power UV LED light was used to initiate the polymerization of oil-phase shell of PMBs. 100 µL PMBs was mixed with 1 mL DMSO/H_2O (1:4, v/v) solution, then was transferred to a 1.5 mL Eppendorf tube or 24 well plate (lumox® multiwall 24, SARSTEDT) for sonication.

Quantification of molecular release: 100 µL PMB-UMB or PMB-CPT was dispersed into 1 mL DMSO/H_2O (1:4, v/v) solution, then transferred into a 1.5 mL Eppendorf tube for 30 min of sonication (20 kHz). Then, 3 mg MCE was added to the mixture to react with covalently loaded **N2-A3** or **N2-B3** in the fragments. 3 d incubation at 37 ^{o}C leading to the occurrence of completely reaction of **N2-A3** (or **N2-B3)** and MCE. The following quantification was conducted by using the same operations in Section 3.1 of the Supplementary Information. With MCE addition, the fluorescence intensity of collected solution was measured at 465 nm (UMB) or 450 nm (CPT), the value of fluorescence intensity as F_m.

Subsequently, we measured the fluorescence intensity of sonicated PMBs (no MCE added) by the same method. Fluorescence intensity at 465 nm or 450 nm of sonicated PMB suspension was collected as F_s. F_m and F_s were used to calculate the percentage of UMB or CPT release based on the standard (fluorescence intensity-concentration) curve of fluorophore.

3.3. Preparation of PMB-PBL

The preparation of PMBs-PBL is similar as detailed in Section 3.1.

G/O/W emulsion: Gas: N_2; Oil phase: PPGDA (500 mg), PPG (50 mg), TPO-L (10 mg), Toluene (440 mg), **N3-A5** (2 mg); H_2O phase: PVA (4.5%, w/w), glycerol (40%, v/v). Flow rate: gas (530 mbar), oil phase (90 nL min^{-1}), H_2O phase (2.3 bar). A high-power UV LED LZ1-10UV0R-0000 (set to 200 mA, Osram) was used to initiate the polymerization of oil-phase shell of PMBs. 100 µL PMBs was dispersed into 1 mL MeCN:MeOH:H_2O (3:1:0.5, v/v/v) solution, then was transferred to a 1.5 mL Eppendorf tube or 24 well plate (lumox® multiwall 24, SARSTEDT) for sonication experiment

Quantification of molecular release: **N3-A4** (1.4 mg, 2.78 µmol) was used to prepare the PMBs (control sample) that contained equal molar mass **N3-A4** compared to the **N3-A5** in PMB-PBL for the quantification of molecular release. The control sample (100 µL PMBs) mixed with 1 mL MeCN:MeOH:H_2O (3:1:0.5), then was transferred to a 1.5 mL Eppendorf tube under 20 kHz US for 30 min. 3 d of incubation (1000 rpm) at 37 °C was implemented to maximize the reaction (decomposition of the free furfuryl carbonate in polar protic solvent). After centrifugation at RCF 8000 ×g for 15 min, then the suspension was filtered through a centrifugal filter (3,000 Da MWCO) by centrifugation at 5,000 rpm. The fluorescence intensity of control sample at 390 nm was measured, the value of fluorescence intensity as F_m.

Then, we measured the fluorescence intensity of sonicated PMB-PBL by the same method. The value of fluorescence intensity at 390 nm was collected as F_s. Finally, F_m and F_s were used to calculate the percentage of PBL release based on the standard (fluorescence intensity-concentration) curve of fluorophore.

3.4. Preparation of PMB-Flex

The preparation of PMB-Flex is similar as detailed in Section 3.1.

G/O/W emulsion: Gas: N_2; Oil phase: PPGDA (500 mg), PPG (50 mg), TPO-L (10 mg), Toluene (440 mg), **N4-A4** (2 mg); H_2O phase: PVA (4.5%, w/w), glycerol (40%, v/v). For the control samples, **N4-A3** or **N4-B4** (2 mg) was used to prepare PMBs. Flow rate: gas (530 mbar), oil phase (90 nL min^{-1}), H_2O phase (2.3 bar). A high-power UV LED LZ1-10UV0R-0000 (set to 200 mA, Osram) was fixated above the outlet tube to initiate the polymerization of oil-phase shell of PMBs. The following steps are the same description in Section 3.1 of the Supplementary Information.

Quantification of molecular release: 100 µL PMB-Flex was mixed with 1 mL MeCN/H_2O (1:1, v/v) solution in a 1.5 mL Eppendorf tube. After US treatment for 30 min (20 kHz), 3 mg MCE was added into the mixture to react with covalently loaded **N4-A4** in the fragments. With 3 d incubation (700 rpm) at 37 °C to make sure **N4-A4** was fully reacted with MCE. After centrifugation at RCF 8000 ×g for 15 min, then the solutions were filtered through a centrifugal filter (3,000 Da MWCO) by centrifugation at 5,000 rpm. The fluorescence intensity at 545 nm of collected solution was measured, the obtained value as F_m.

Subsequently, we measured the fluorescence intensity of sonicated PMB-Flex (no MCE addition) by the same method. We collected the fluorescence intensity at 545 nm as F_s. Finally, F_m and F_s were used to calculate the percentage of molecular release based on the standard (fluorescence intensity-concentration) curve of fluorophore.

3.5. Preparation of microgels

3.5.1. Preparation of microgels with masked dansyl-fluorophore

Microgels were prepared using O/W single emulsion method. The oil phase solution was injected into the microfluidic device by a syringe pump. Then, the continuous aqueous phase solution was loaded into the microfluidic device by flow controller. Once the flow rate of each phase reaches the desired value, the single emulsion production in the microfluidic device proceeds automatically. The microgels were collected into a glass vial. Finally, microgels were washed for 3× using DI H_2O before sonication.

O/W emulsion: Oil phase: PPGDA (840 mg), PPG (50 mg), TPO-L (10 mg), **N1-CL** (100 mg), **N1-DA2** (2 mg); H_2O phase: PVA (3%, w/w). Flow rate: Oil phase (150 nL min^{-1}), H_2O phase (0.27 bar). A high-power UV LED LZ1-10UV0R-0000 (set to 400 mA, Osram) was fixated above the outlet tube to initiate the polymerization of oil-phase. Then, 30 µL microgels was mixed with 1 mL MeCN/H_2O (1:1, v/v) solution in a 1.5 mL Eppendorf tube or 24 well plate (lumox® multiwall 24, SARSTEDT) for sonication.

Quantification of molecular release: 30 µL microgels was mixed with 1 mL MeCN/H_2O (1:1, v/v) in a 1.5 mL Eppendorf tube. Then, 3 µL MCE (excess to **N1-DA2**) were added to the mixture. The subsequent incubation (700 rpm) at 37 °C for 3 d made sure all the covalently loaded **N1-DA2** in the microgels reacted with MCE. After centrifugation at RCF 8000 ×g for 15 min, the fluorescence intensity at 545 nm of the collected solution was measured, the obtained value of fluorescence intensity at 545 nm as F_m.

Subsequently, we measured the fluorescence intensity of sonicated microgels (no MCE addition) by the same method. We collected the fluorescence intensity at 545 nm as F_s. Finally, F_m and F_s were used to calculate the percentage of molecular release based on the standard (fluorescence intensity-concentration) curve of fluorophore.

3.5.2. Preparation of microgels with flex-mechanophores (microgels-Flex)

Microgels with 'flex-activated' mechanophores were prepared in O/W single emulsion microfluidic device. The oil phase solution was injected into the microfluidic device by syringe pump. Then, the aqueous phase solution was loaded into the microfluidic device. Once the flow rate of each phase reaches the desired value, the single emulsion production in the microfluidic device proceeds automatically. The microgels was collected into a glass vial. Finally, microgels was washed 3× using DI H_2O before sonication.

O/W emulsion: Oil phase: PPGDA (940 mg), PPG (50 mg), TPO-L (10 mg), **N4-A4** (2 mg); H_2O phase: PVA (3%, w/w). Flow rate: Oil phase (150 nL min^{-1}), H_2O phase (0.27 bar). A high-power UV LED LZ1-10UV0R-0000 (set to 400 mA, Osram) was fixated above the outlet tube to initiate the polymerization of oil-phase. Then, 30 µL microgels were mixed with 1 mL MeCN/H_2O (1:1, v/v) solution in a 1.5 mL Eppendorf tube or 24 well plate (lumox® multiwall 24, SARSTEDT) for sonication.

Quantification of molecular release: 30 µL microgels was mixed with 1 mL MeCN/H_2O (1:1, v/v) in a 1.5 mL Eppendorf tube. Then, 3 µL MCE (excess to **N4-A4**) were added to the mixture. The subsequent 3 d incubation (700 rpm) at 37 °C made sure all the covalently loaded **N4-A4** in microgels reacted with MCE. After centrifugation at RCF 8000 ×g for 15 min, the fluorescence intensity at 545 nm of the collected solution was measured, the obtained value of fluorescence intensity at 545 nm as F_m.

Subsequently, we measured the fluorescence intensity of sonicated microgels (no MCE addition) by the same method. The collected fluorescence intensity at 545 nm as F_s. Finally, F_m and F_s were used to calculate the percentage of molecular release based on the standard (fluorescence intensity-concentration) curve of fluorophore.

4. Supplementary figures

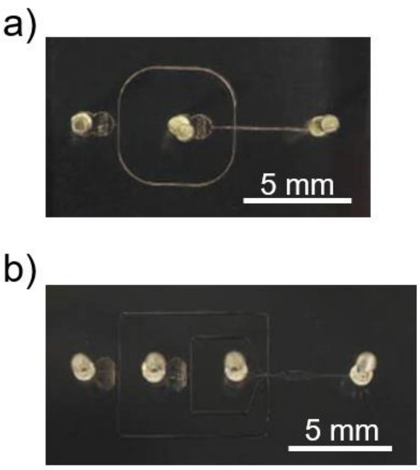

Supplementary Figure 6. The images of microfluidic chips for production of (**a**) O/W emulsion, and (**b**) G/O/W emulsion.

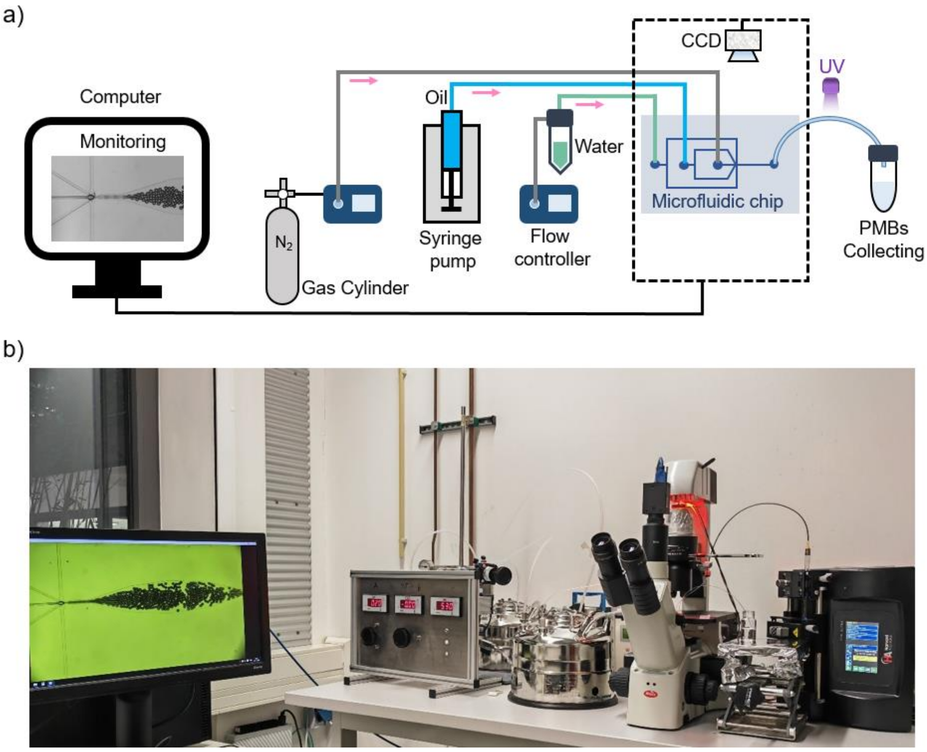

Supplementary Figure 7. Microfluidic system. (**a**) Schematic illustration of the working process. (**b**) Photograph of the setup.

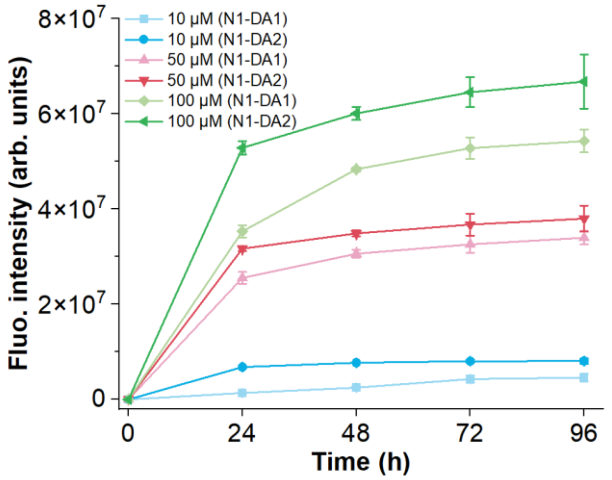

Supplementary Figure 8. The kinetics analysis of 2-mercaptoethanol (MCE) reacted with **N1-DA1** and **N1-DA2** (**N1-DA1** or **N1-DA2** mixed with excess MCE (10 mM) in MeCN/H_2O). Data are presented as mean values +/− the standard deviation. *N* = 3 independent sonications.

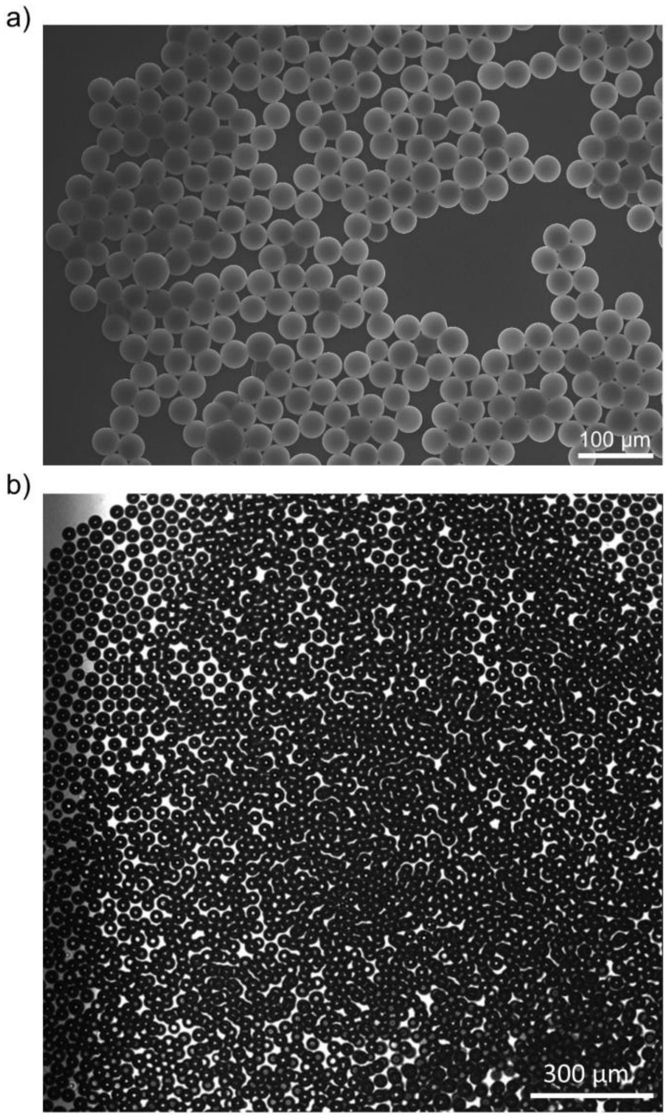

Supplementary Figure 9. PMB-MDF. (**a**) SEM micrograph. Three times the experiment was repeated, and similar images were obtained. (**b**) Brightfield optical microscopy micrograph. Three times the experiment was repeated, and similar images were obtained.

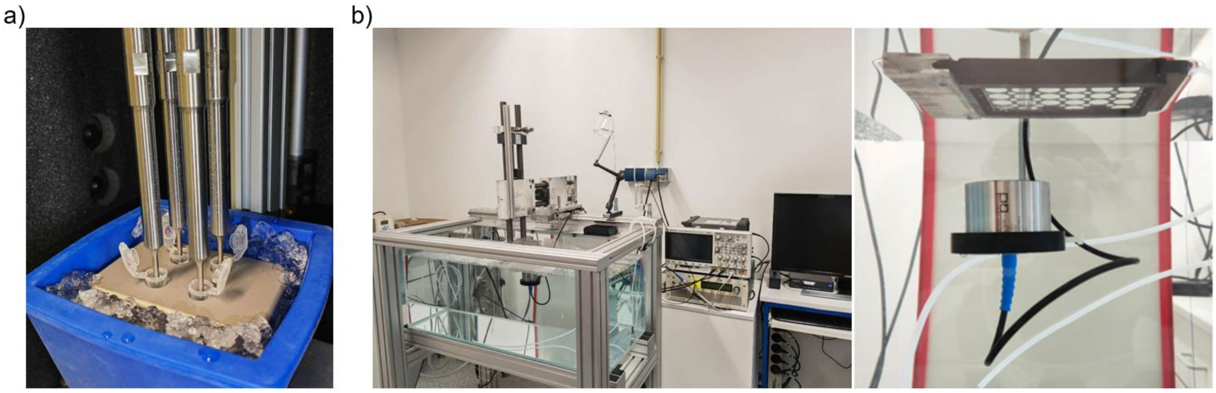

Supplementary Figure 10. Sonication systems. (**a**) 20 kHz 4-probe sonicator. (**b**) HIFU system (left), transducer and 24-well plate (right).

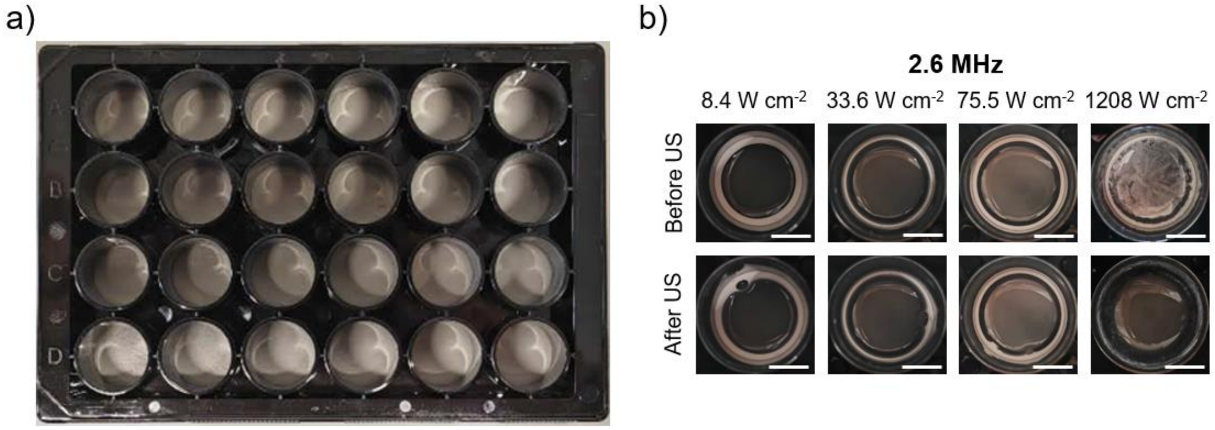

Supplementary Figure 11. HIFU experiments. (**a**) 24-well plate with an acoustically transparent base for HIFU experiments. (**b**) The response of PMBs to different sound intensity irradiations (2.6 MHz, 5 min), scale bar: 5 mm.

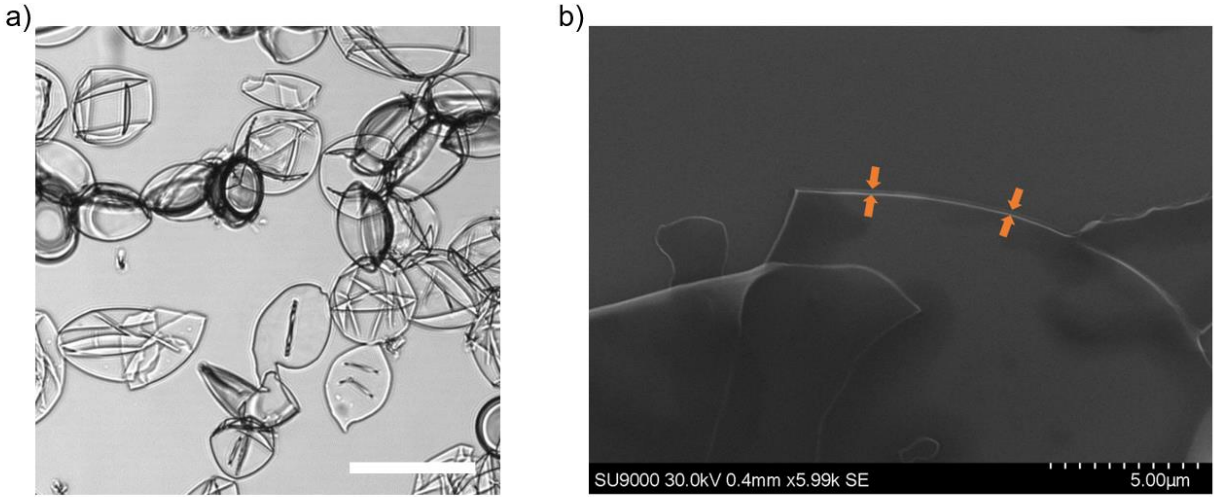

Supplementary Figure 12. Images of PMB fragments. (**a**) Confocal microscopy image of PMB fragments after 2.6 MHz ultrasound irradiation (1208 W cm^{-2}, 15 min), scale bar: 50 μm. Three times the experiment was repeated, and similar images were obtained. (**b**) SEM image of fragments of PMB-MDF. The shell thickness of PMBs is around 230 nm. Three times the experiment was repeated, and similar images were obtained.

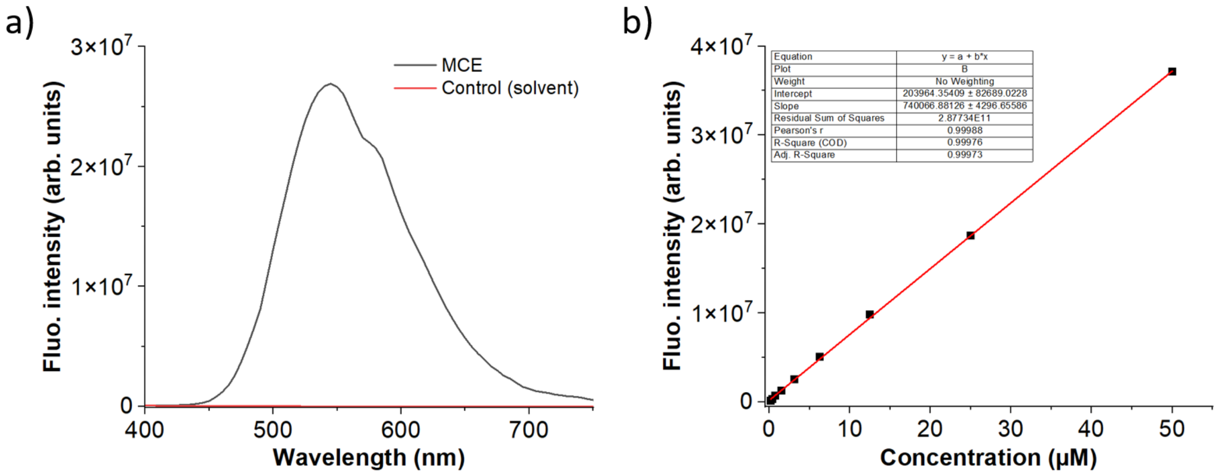

Supplementary Figure 13. Standard curve of dansyl-fluorophore. (**a**) Fluorescence spectrum of PMBs-MDF mixed with an excess of MCE (solvent: MeCN/H_2O). (**b**) Standard curve of dansyl-fluorophore (**N1-A3**), the relationship between concentration and fluorescence intensity at 545 nm (λ_{exc} = 340 nm).

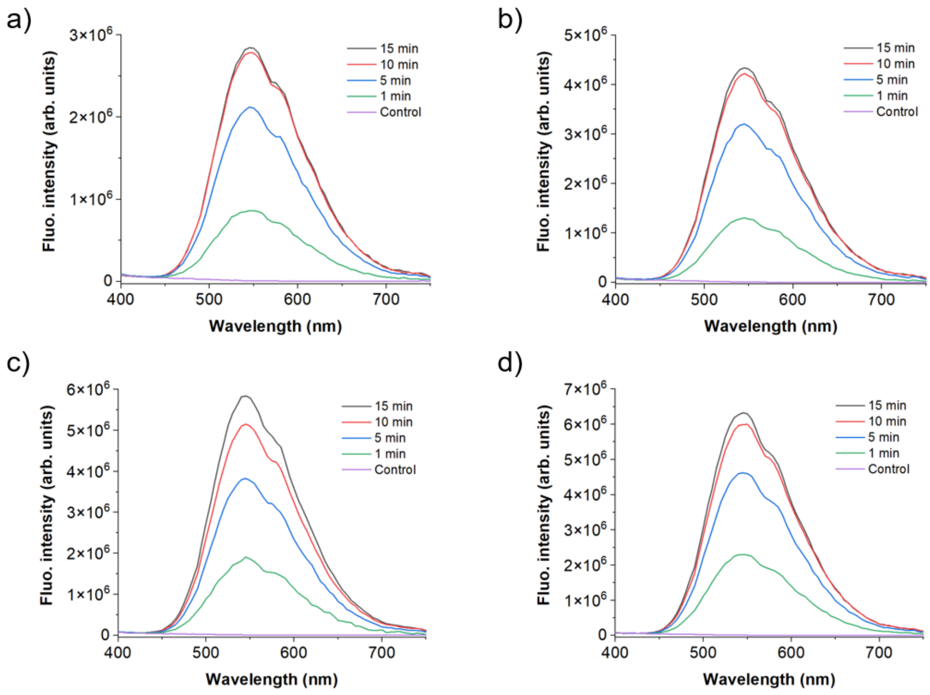

Supplementary Figure 14. Fluorescence spectra of PMB-MDF suspension (solvent: MeCN/H_2O) under the treatment of 20 kHz sonication with the intensity of (**a**) 1 W cm^{-2}, (**b**) 3 W cm^{-2}, (**c**) 6 W cm^{-2}, and (**d**) 12 W cm^{-2}.

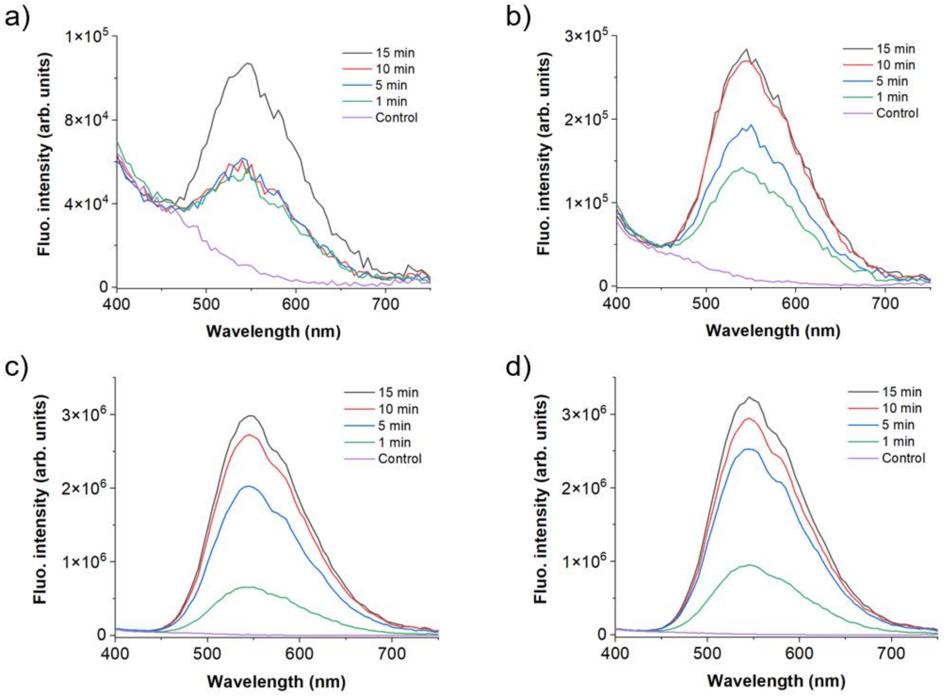

Supplementary Figure 15. Fluorescence spectra of PMB-MDF suspension (solvent: MeCN/H_2O) under 0.68 MHz irradiation with the sound intensity of (**a**) 8.4 W cm^{-2}, (**b**) 33.6 W cm^{-2}, (**c**) 75.5 W cm^{-2}, and (**d**) 134.2 W cm^{-2}.

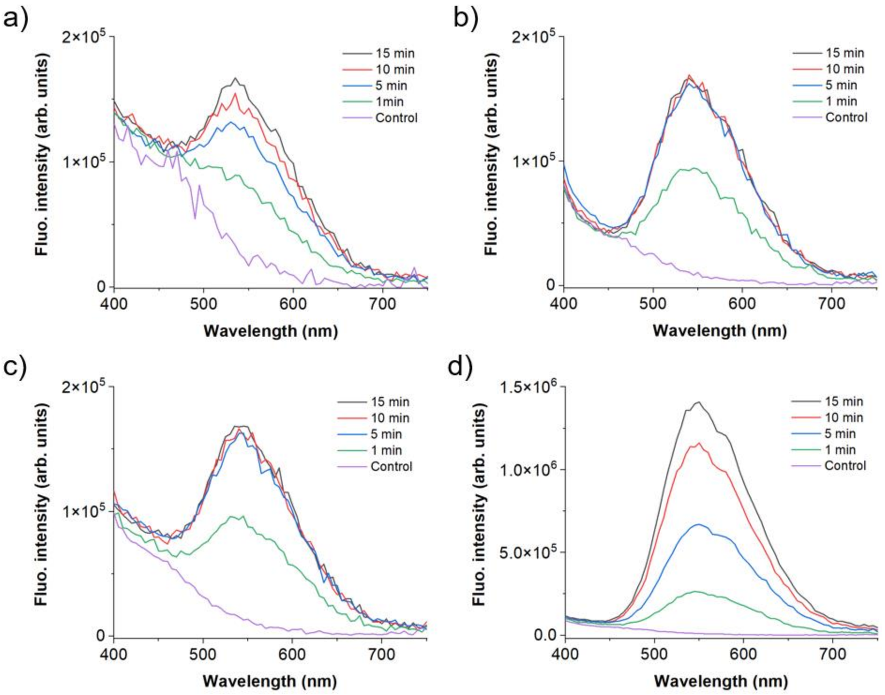

Supplementary Figure 16. Fluorescence spectra of PMB-MDF suspension (solvent: MeCN/H_2O) under 1.52 MHz irradiation with the sound intensity of (**a**) 8.4 W cm^{-2}, (**b**) 33.6 W cm^{-2}, (**c**) 75.5 W cm^{-2}, and (**d**) 134.2 W cm^{-2}.

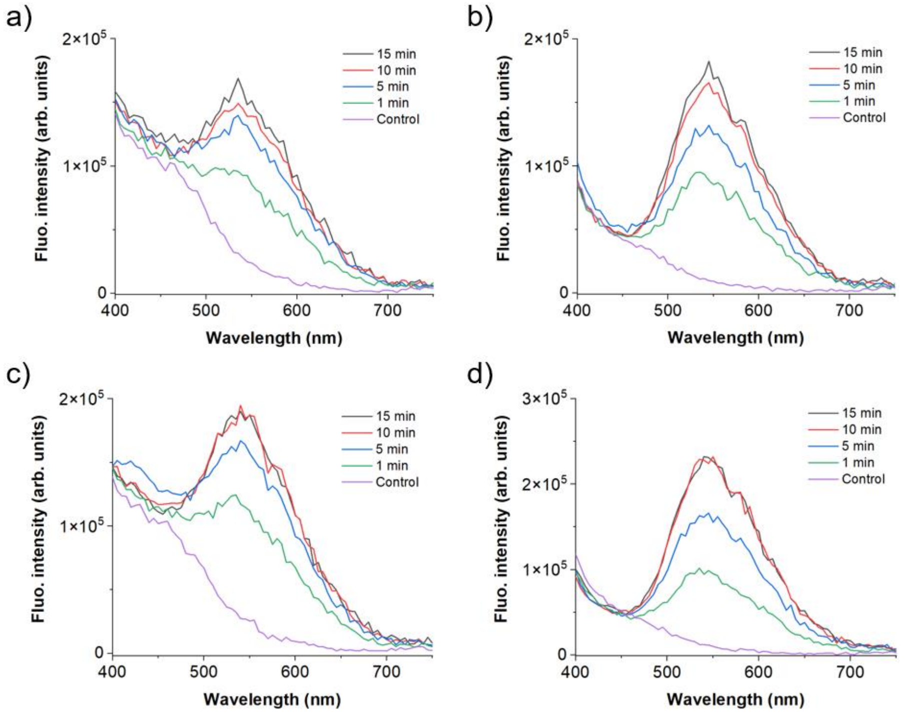

Supplementary Figure 17. Fluorescence spectra of PMB-MDF suspension (solvent: MeCN/H_2O) under 2.6 MHz irradiation with the sound intensity of (**a**) 8.4 W cm^{-2}, (**b**) 33.6 W cm^{-2}, (**c**) 75.5 W cm^{-2}, and (**d**) 134.2 W cm^{-2}.

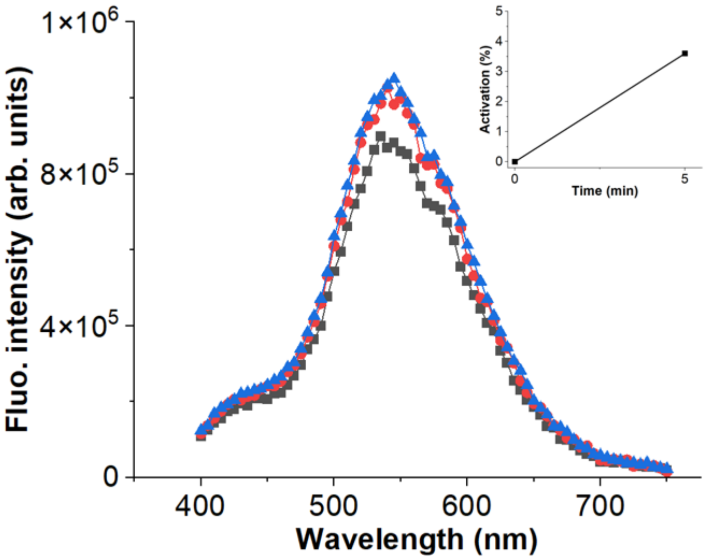

Supplementary Figure 18. Fluorescence spectra collected after sonication with a very low sound intensity (5 min, 20 KHz, 0.2 W cm^{-2}). Data are from 3 independent sonications.

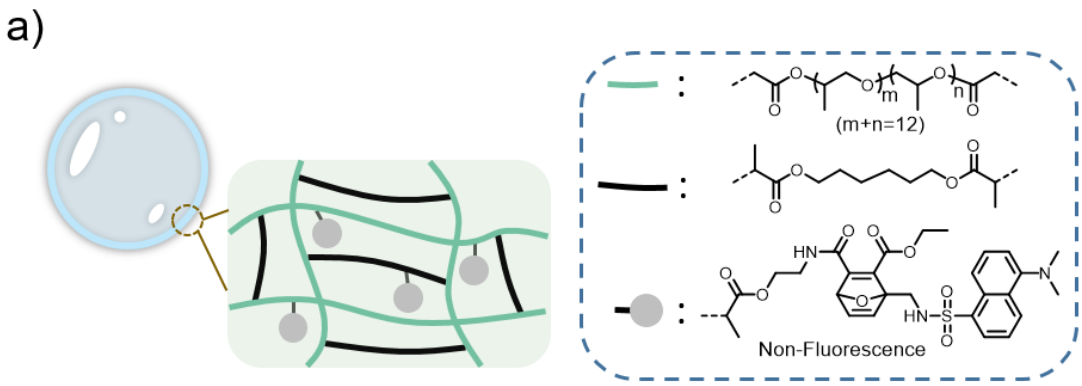

Supplementary Figure 19. Scheme of PMBs without disulfide mechanophores in polymeric shell.

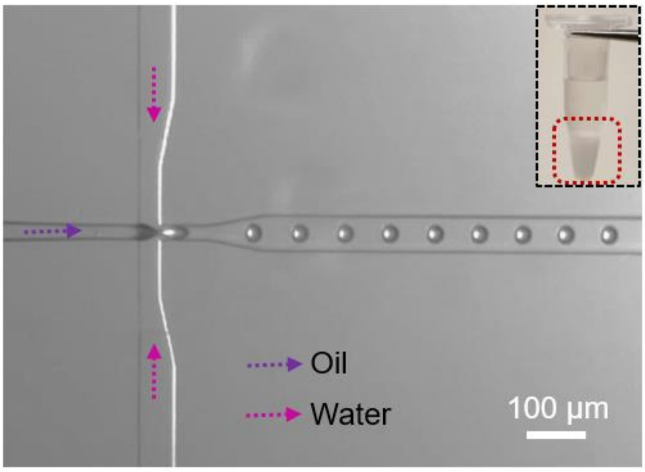

Supplementary Figure 20. The production image of microgels in the microfluidic chip. Three times the experiment was repeated, and similar images were obtained.

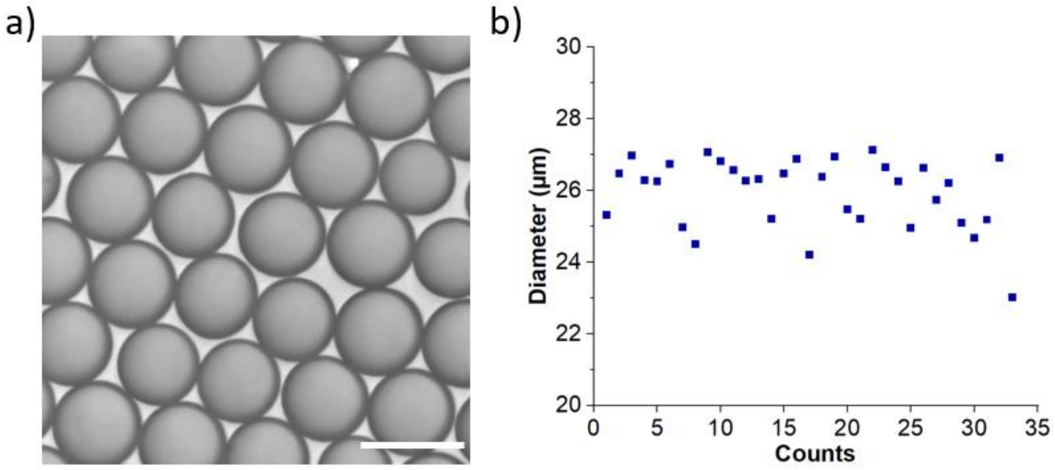

Supplementary Figure 21. Size distribution of microgels. (**a**) Microscopy image of microgels, scale bar: 30 µm. Three times the experiment was repeated, and similar images were obtained. (**b**) Size distribution of produced microgels.

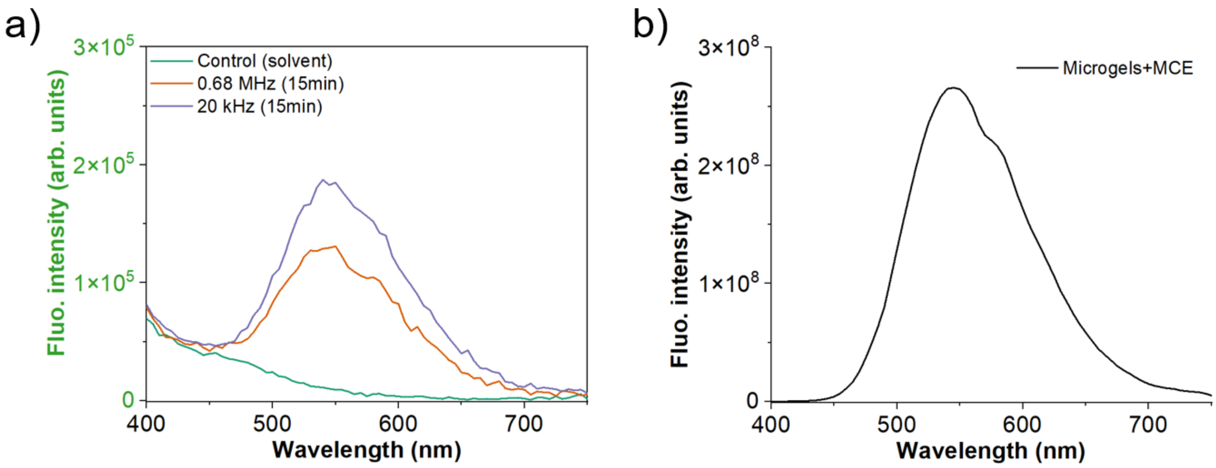

Supplementary Figure 22. Mechanophore activation in microgels. (**a**) The fluorescence spectra of microgels under US irradiation (20 kHz, 12 W cm^{-2}; 0.68 MHz, 134.2 W cm^{-2}). (**b**) The fluorescence spectra of microgel suspension with the treatment of excess MCE (solvent: MeCN/H_2O).

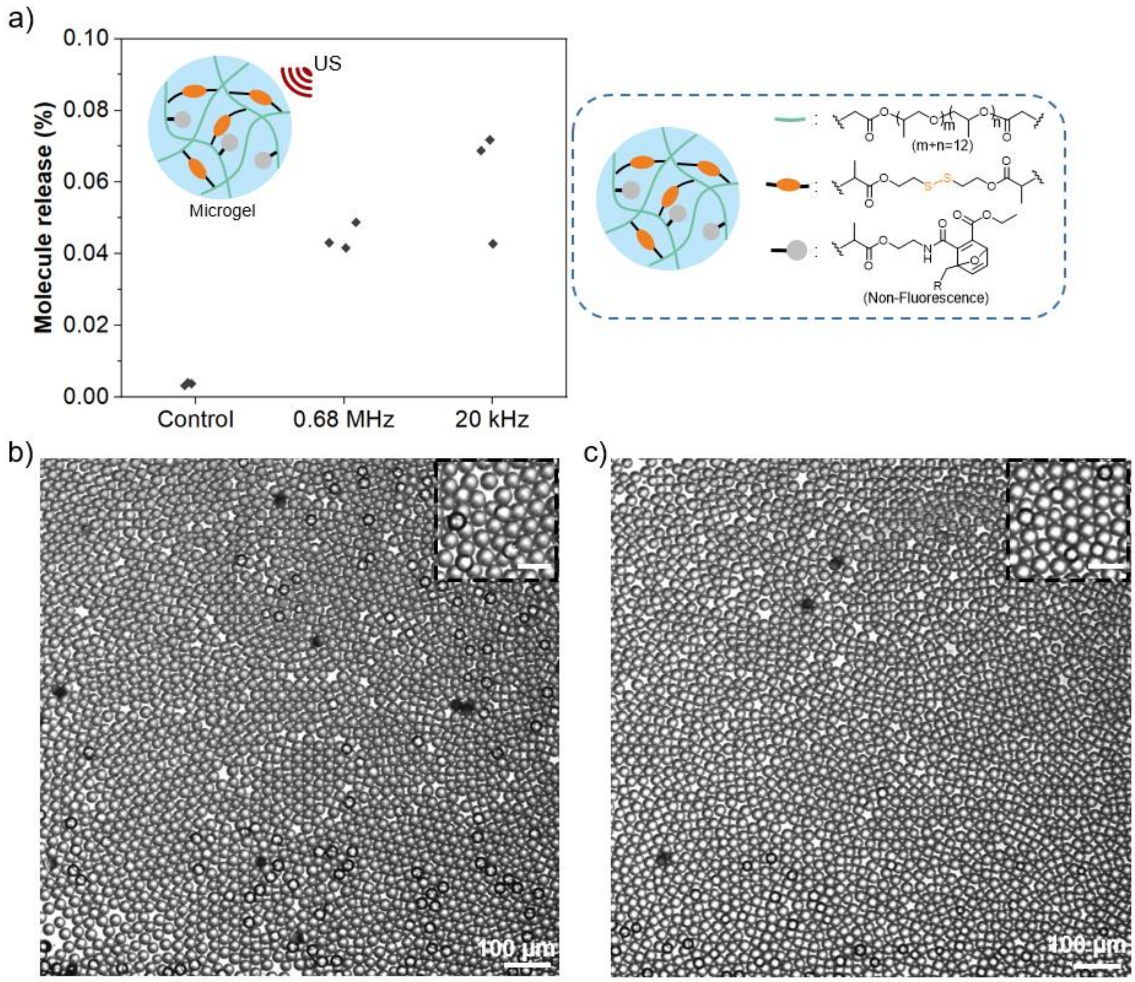

Supplementary Figure 23. Mechanophore in microgels. (**a**) Fluorophores release from microgels after 15 min US irradiations. Optical brightfield micrographs of microgels. Data from 3 independent sonications. (**b**) before and (**c**) after sonication (20 kHz, 15 min). Inset: confocal microscopy image, scale bar: 50 µm. Three times the experiment was repeated, and similar images were obtained.

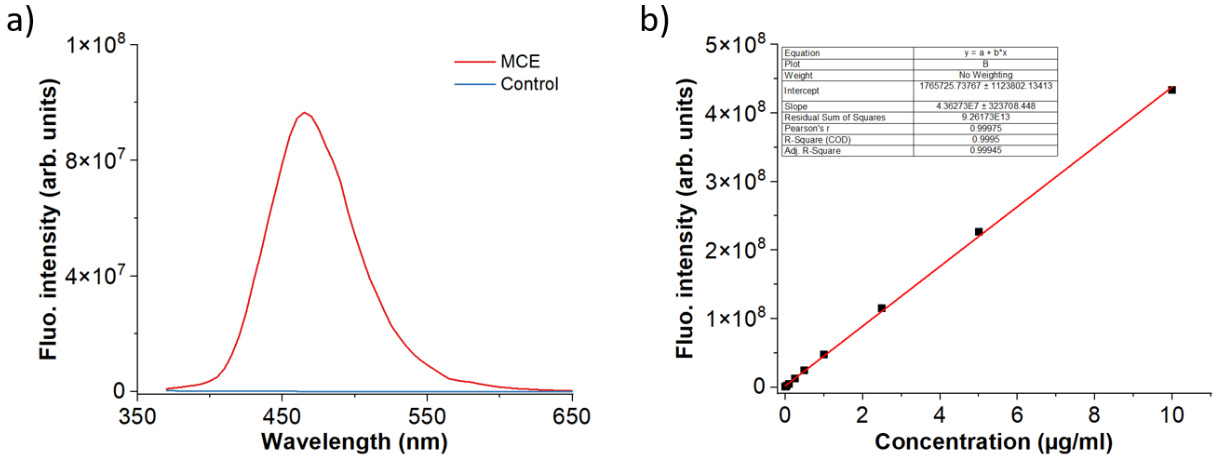

Supplementary Figure 24. Standard curve of UMB. (**a**) The fluorescence spectrum of PMB-UMB suspension with the treatment of excess MCE (solvent: DMSO/H_2O). (**b**) The relationship between concentration and fluorescence intensity at 465 nm (λ_{exc} = 325 nm).

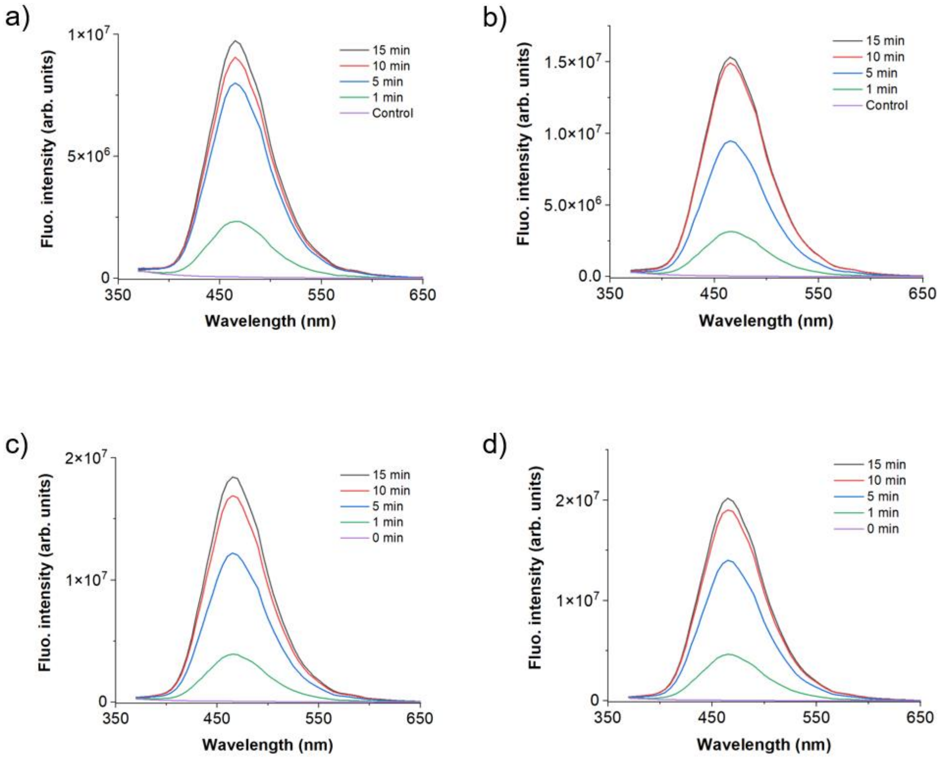

Supplementary Figure 25. Fluorescence spectra of PMB-UMB suspension (solvent: DMSO/H_2O) under the treatment of 20 kHz sonication with the intensity of (**a**) 1 W cm^{-2}, (**b**) 3 W cm^{-2}, (**c**) 6 W cm^{-2}, and (**d**) 12 W cm^{-2}.

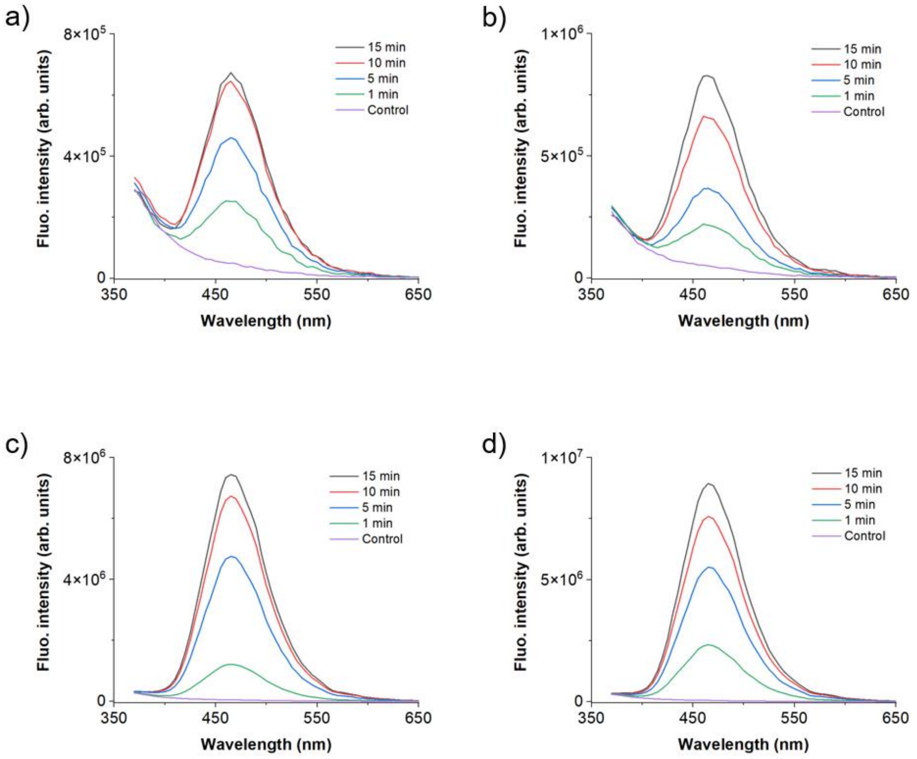

Supplementary Figure 26. Fluorescence spectra of PMB-UMB suspension (solvent: DMSO/H_2O) under the treatment of 0.68 MHz sonication with the sound intensity of (**a**) 8.4 W cm^{-2}, (**b**) 33.6 W cm^{-2}, (**c**) 75.5 W cm^{-2}, and (**d**) 134.2 W cm^{-2}.

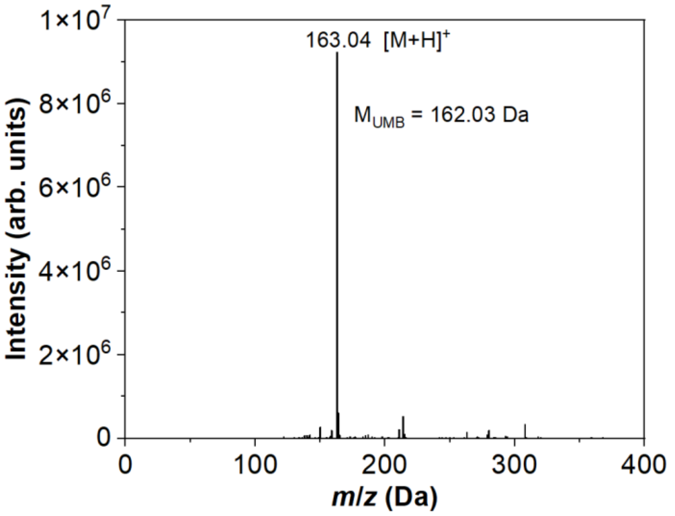

Supplementary Figure 27. LC-MS result obtained from PMB-UMB suspension after US treatment (20 kHz).

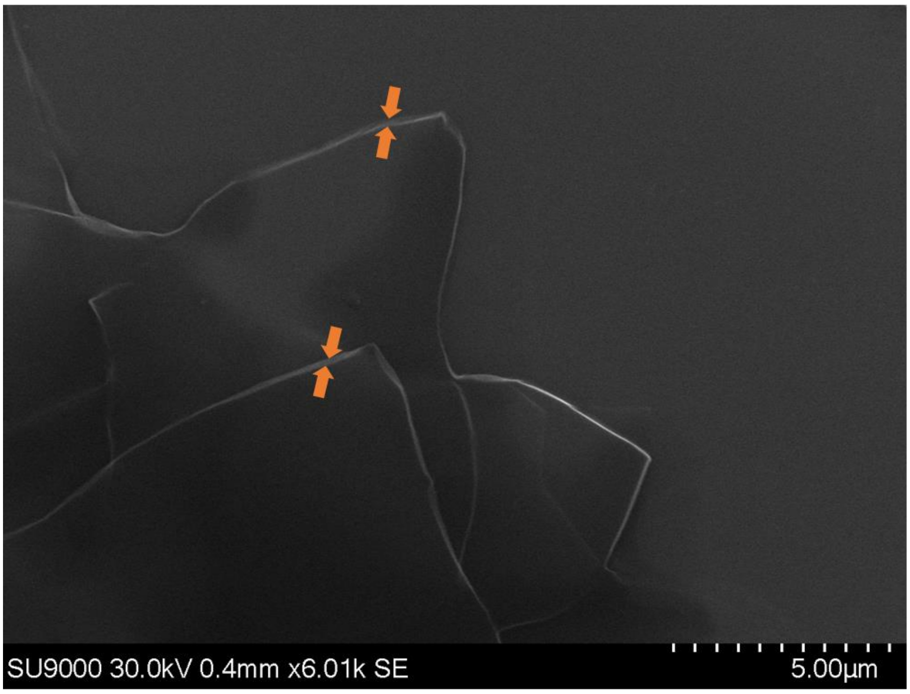

Supplementary Figure 28. SEM images of fragments from PMB-UMB. The shell thickness of PMB-UMB is around 235 nm. Three times the experiment was repeated, and similar images were obtained.

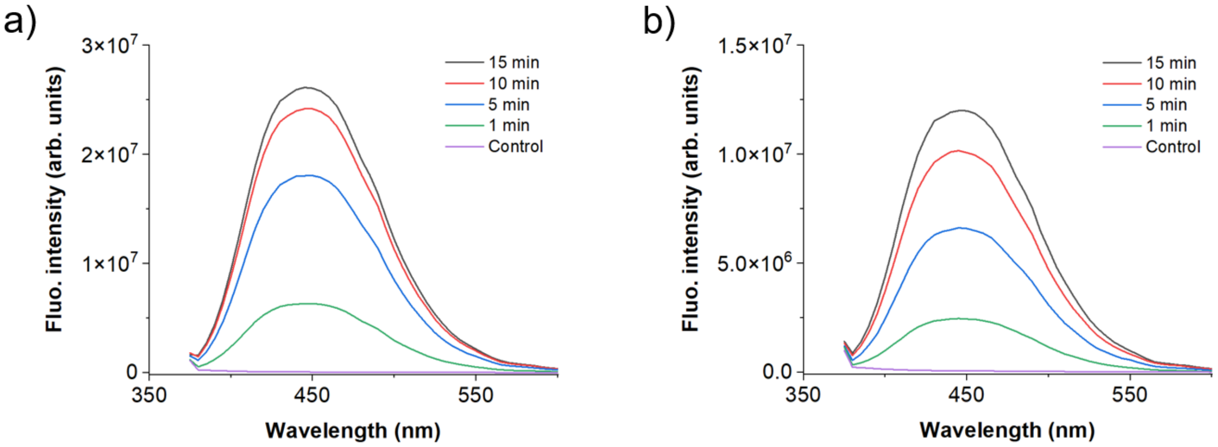

Supplementary Figure 29. Fluorescence spectra of PMB-CPT suspension (solvent: DMSO/H_2O) after treatment with (**a**) 20 kHz sonications (12 W cm^{-2}) and (**b**) 0.68 MHz ultrasound irradiation (134.2 W cm^{-2}).

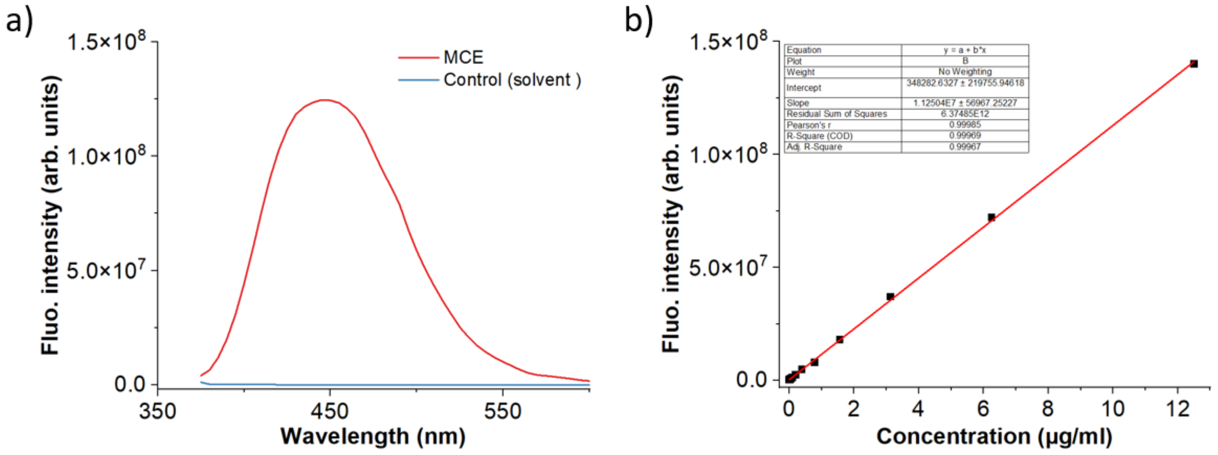

Supplementary Figure 30. Standard curve of CPT. (**a**) The fluorescence spectrum of PMB-CPT suspension with the addition of excess MCE (solvent: DMSO/H_2O). (**b**) The relationship between concentration and fluorescence intensity at 450 nm (λ_{exc} = 335 nm).

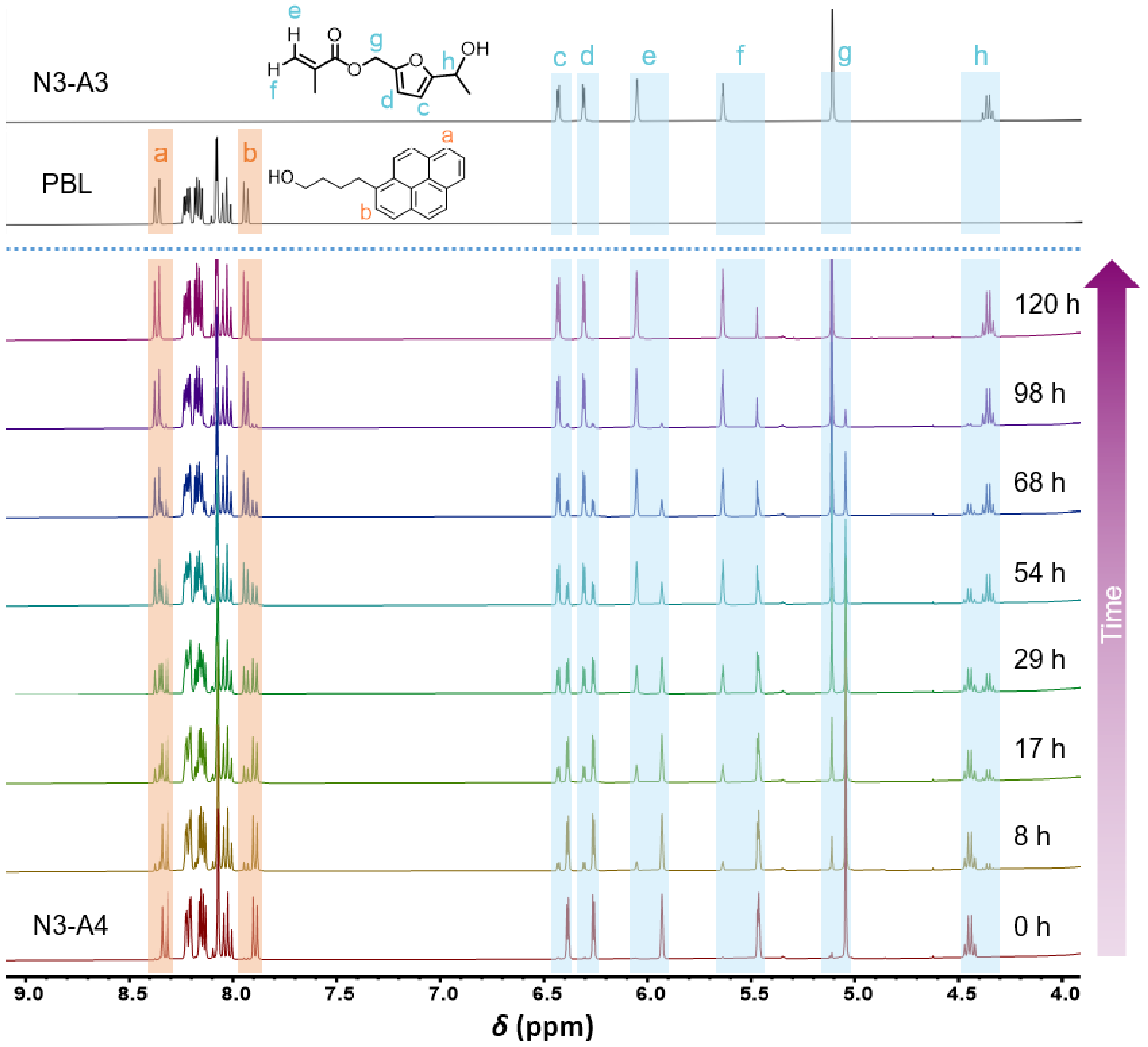

Supplementary Figure 31. ^{1}H NMR spectra (400 MHz) demonstrating the conversion of **N3-A4** to **N3-A3** and PBL at 23 °C (5 mg **N3-A4** in 0.5 mL solvent (MeCN-d_3:MeOH:H_2O, 3:1:0.5)).

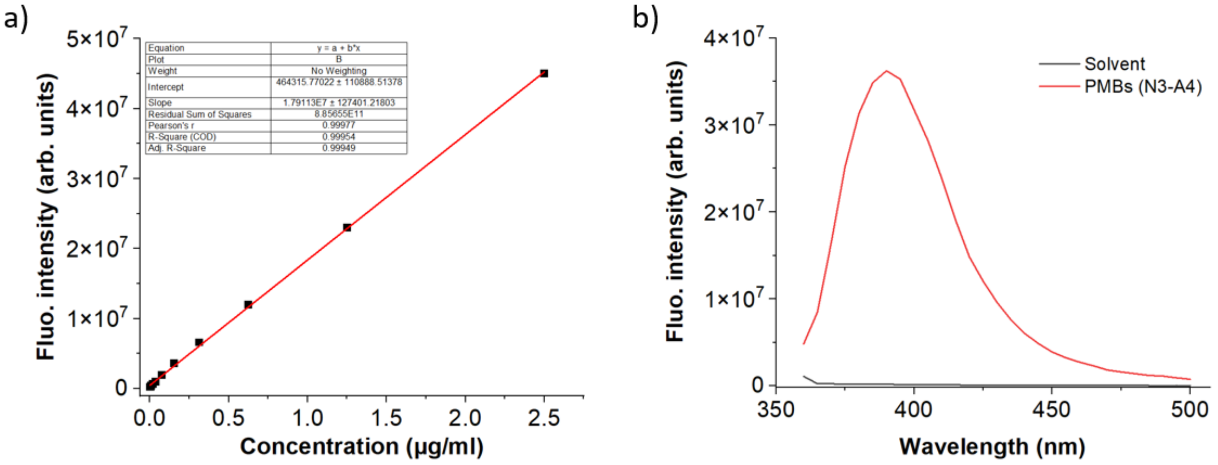

Supplementary Figure 32. Standard curve of PBL. (**a**) The relationship between concentration and fluorescence intensity at 390 nm (solvent: MeCN/MeOH/H_2O, λ_{exc} = 320 nm). (**b**) The fluorescence spectra of PMBs with **N3-A4** for the quantification of PBL release.

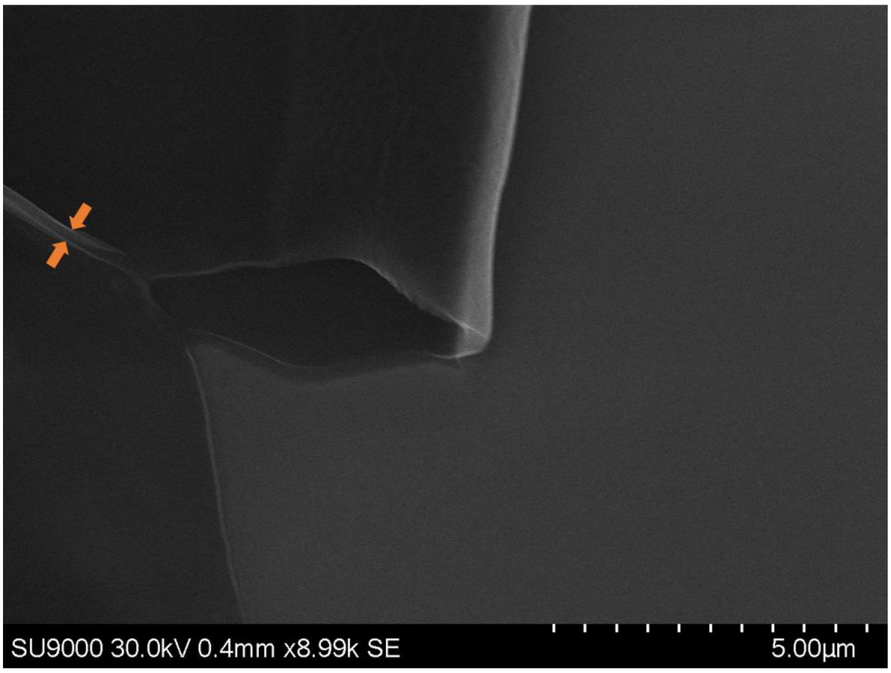

Supplementary Figure 33. SEM image of fragments of PMB-PBL. The shell thickness of PMBs is around 220 nm. Three times the experiment was repeated, and similar images were obtained.

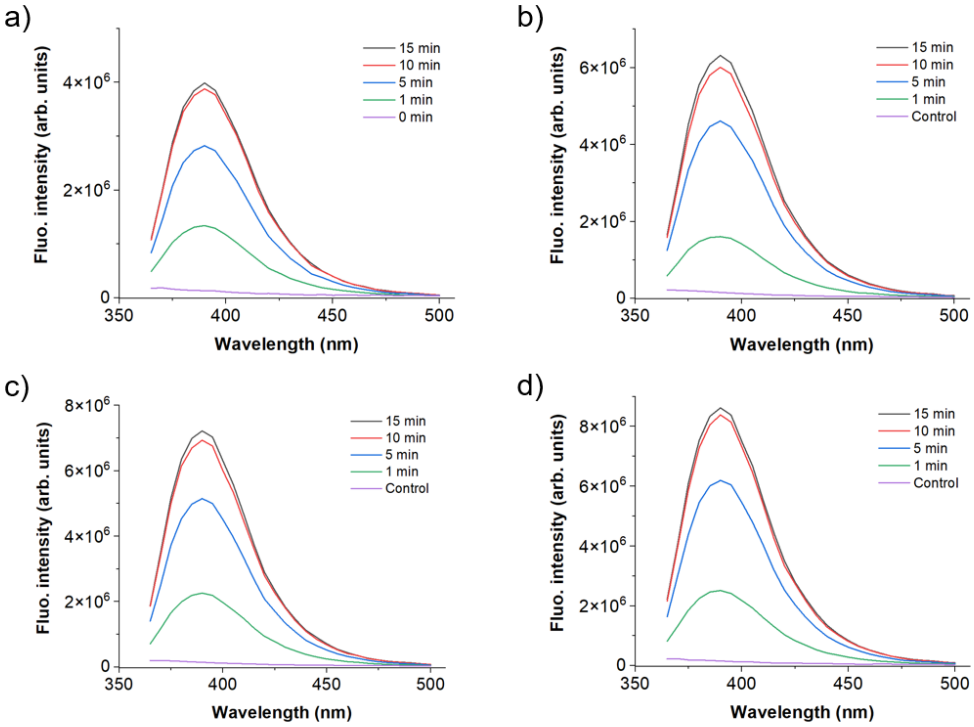

Supplementary Figure 34. Fluorescence spectra of PMB-PBL suspension (solvent: MeCN/MeOH/H_2O) under the treatment of 20 kHz sonication with the intensity of (**a**) 1 W cm^{-2}, (**b**) 3 W cm^{-2}, (**c**) 6 W cm^{-2}, and (**d**) 12 W cm^{-2}.

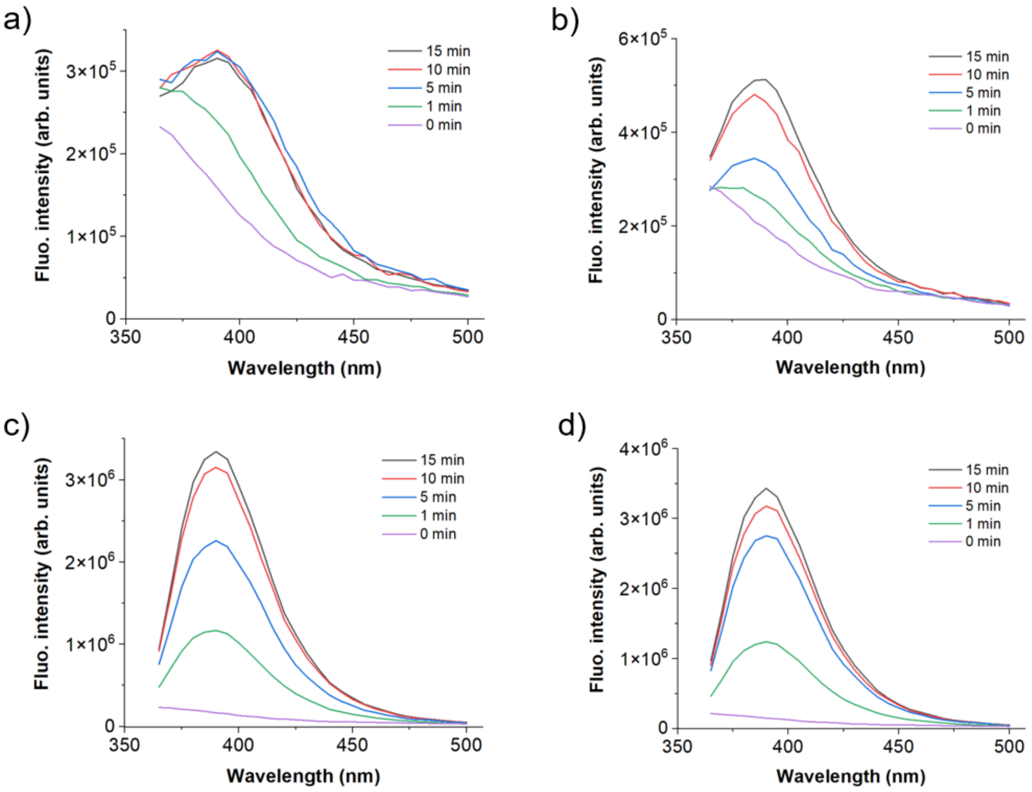

Supplementary Figure 35. Fluorescence spectra of PMB-PBL suspension (solvent: MeCN/MeOH/H_2O) under the treatment of 0.68 MHz sonication with the sound intensity of (**a**) 8.4 W cm^{-2}, (**b**) 33.6 W cm^{-2}, (**c**) 75.5 W cm^{-2}, and (**d**) 134.2 W cm^{-2}.

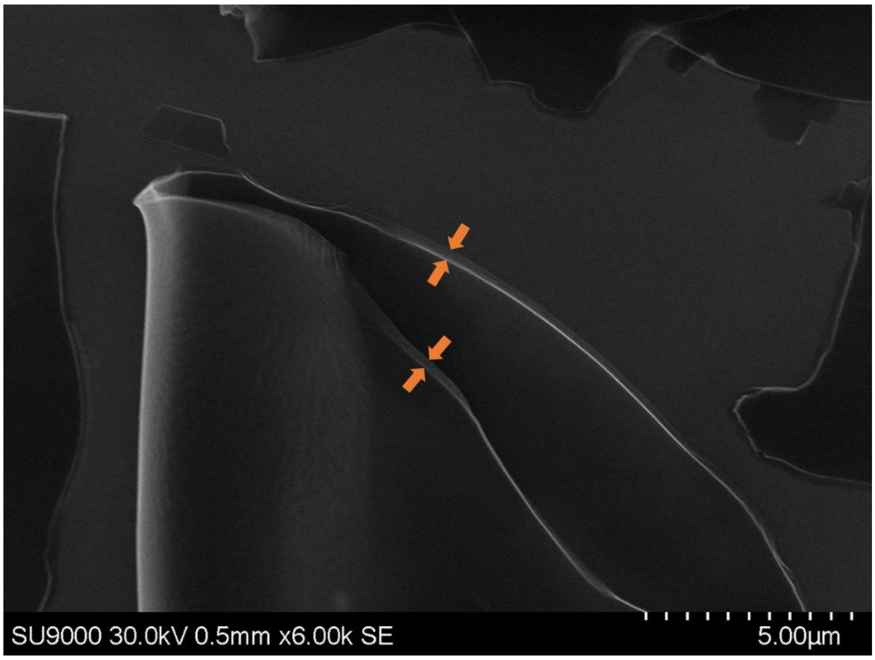

Supplementary Figure 36. SEM image of fragments of PMB-Flex after HIFU treatment. The shell thickness of PMBs is around 230 nm. Three times the experiment was repeated, and similar images were obtained.

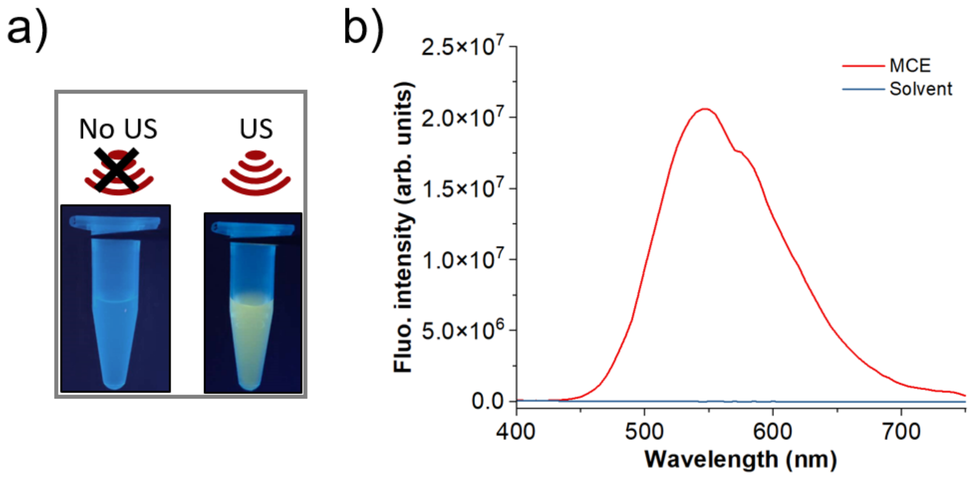

Supplementary Figure 37. Flex-mechanophore activation. (**a**) Photographs of filtered PMB-Flex solution before (left) and after (right) the 20 kHz sonication (15 min; solvent: MeCN/H_2O, 365 nm hand lamp UV illumination). (**b**) Fluorescence spectra of PMB-Flex mixed with excess MCE.

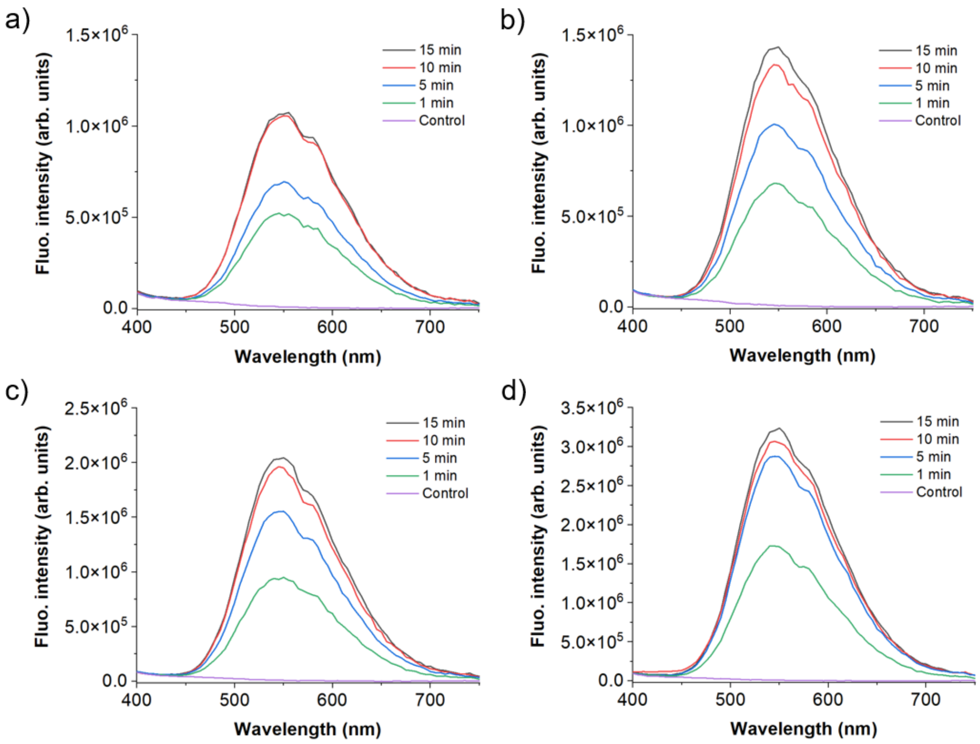

Supplementary Figure 38. Fluorescence spectra of PMB-Flex suspension (solvent: MeCN/H_2O) under the treatment of 20 kHz sonication with the intensity of (**a**) 1 W cm^{-2}, (**b**) 3 W cm^{-2}, (**c**) 6 W cm^{-2}, and (**d**) 12 W cm^{-2}.

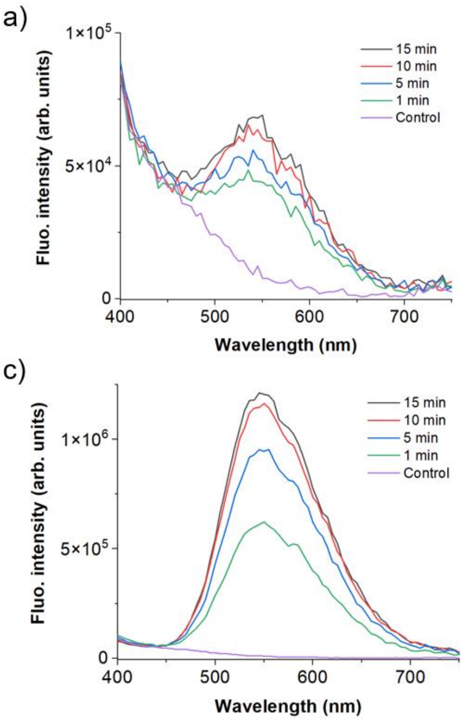

Supplementary Figure 39. Fluorescence spectra of PMB-Flex suspension (solvent: MeCN/H_2O) under the treatment of 0.68 MHz sonication with the sound intensity of (**a**) 8.4 W cm^{-2}, (**b**) 33.6 W cm^{-2}, (**c**) 75.5 W cm^{-2}, and (**d**) 134.2 W cm^{-2}.

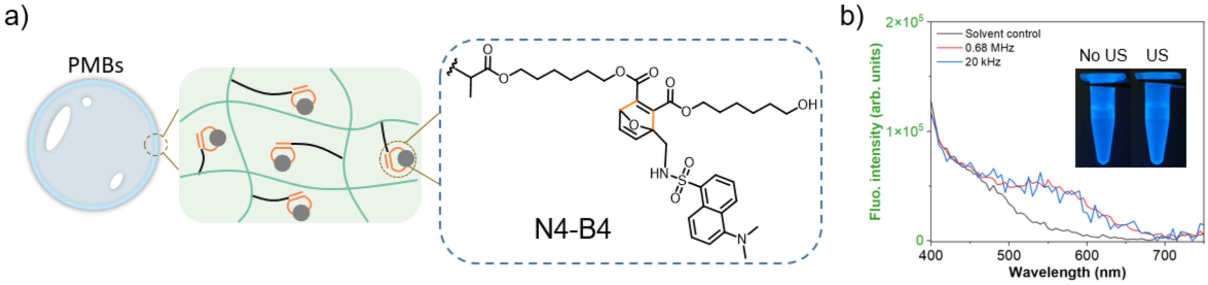

Supplementary Figure 40. PMBs with the polymeric shell contains monofunctional acrylate-mechanophores (**N4-B4**). (**a**) The shell structure of PMBs. (**b**) Fluorescence spectra of PMBs before and after US irradiation (20 KHz, 12 W/cm^2, 15 min). Insets: photographs of filtered PMBs suspension before and after the treatment of 20 kHz sonication (solvent: MeCN/H_2O, under 365 nm hand lamp UV illumination).

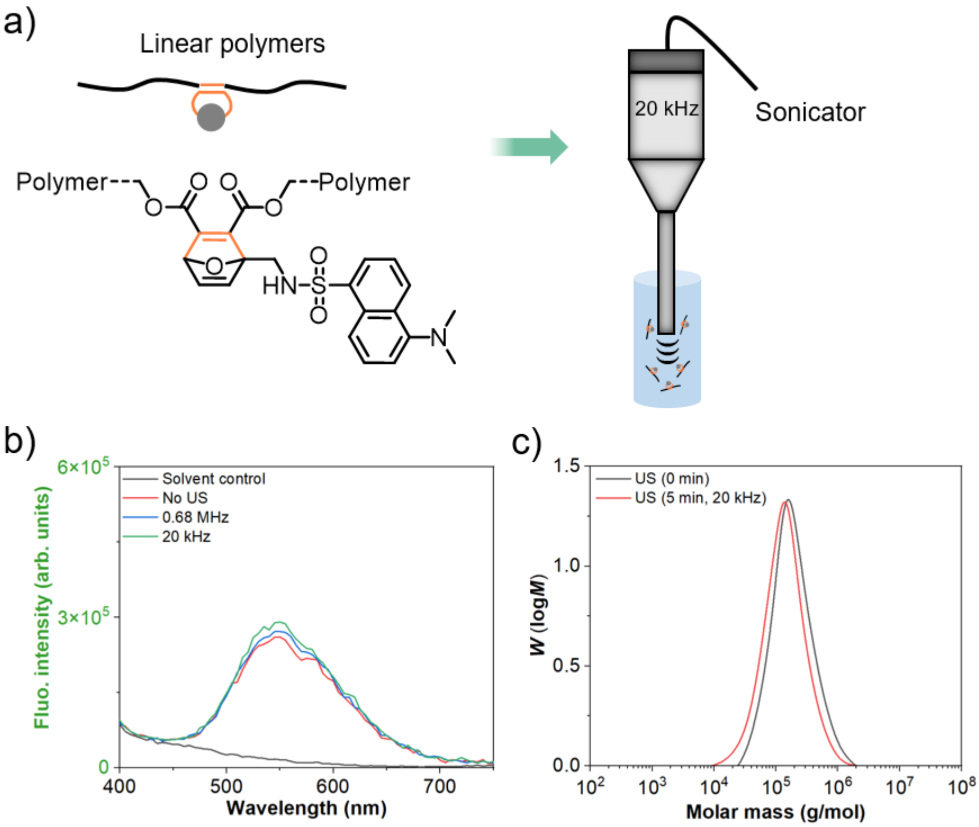

Supplementary Figure 41. Flex-mechanophore in linear polymers (**a**) Representation of mechanophore-centered LPs upon sonication. (**b**) Fluorescence spectra of LPs before and after US. (**c**) GPC RI molar mass distributions of LPs before and after sonication. Insets: photographs of LP solutions under 365 nm UV in MeCN/H_2O.

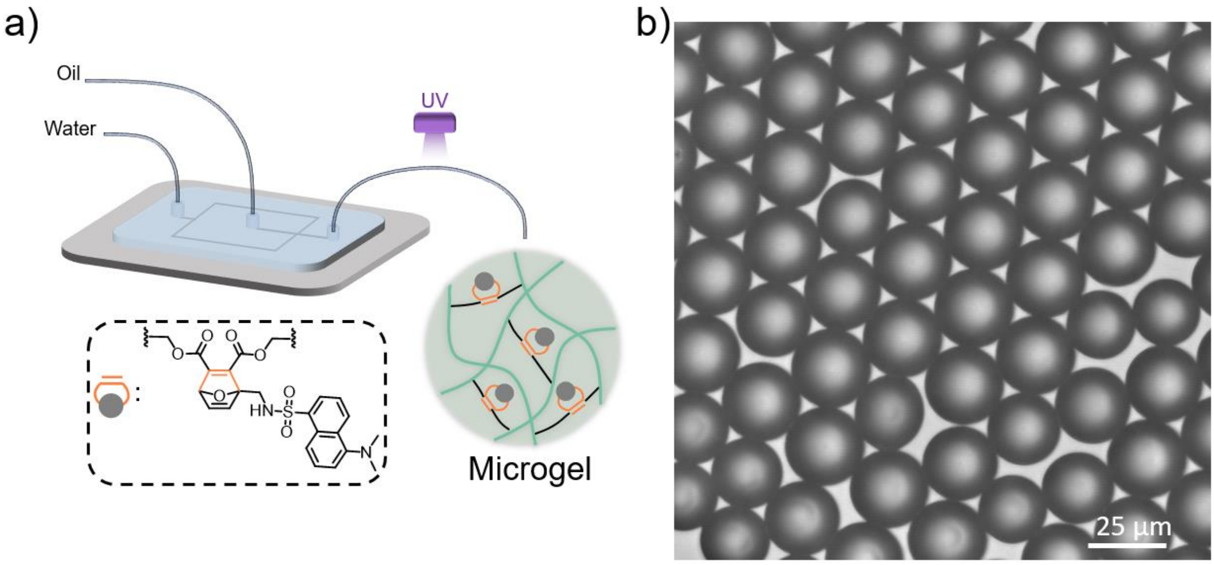

Supplementary Figure 42. Preparation of microgels. (**a**) Schematic production of microgels in the microfluidic device. (**b**) Optical brightfield micrograph of Microgels-Flex. Three times the experiment was repeated, and similar images were obtained.

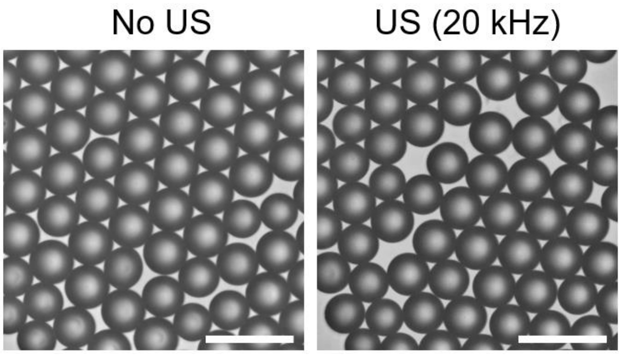

Supplementary Figure 43. Optical brightfield micrograph of microgels before and after 5 min US irradiation (scale bar: 50 µm). Three times the experiment was repeated, and similar images were obtained.

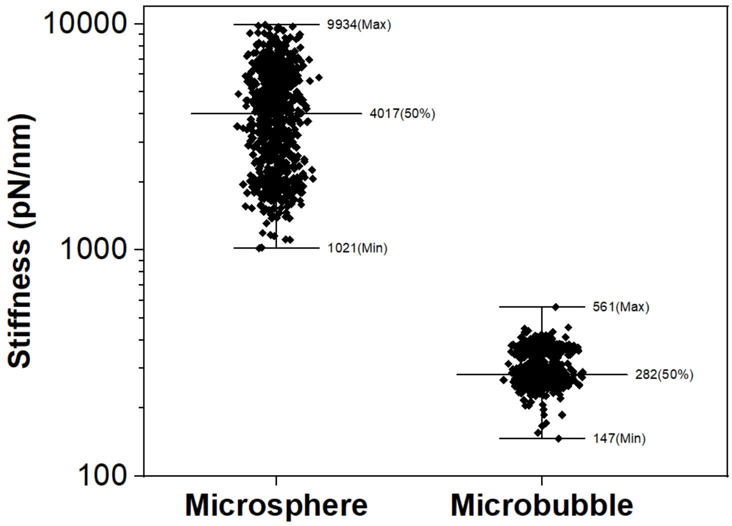

Supplementary Figure 44. Stiffness of PMB-Flex and Microgels-Flex measured by AFM. Minima, maxima and median are shown in the figure.

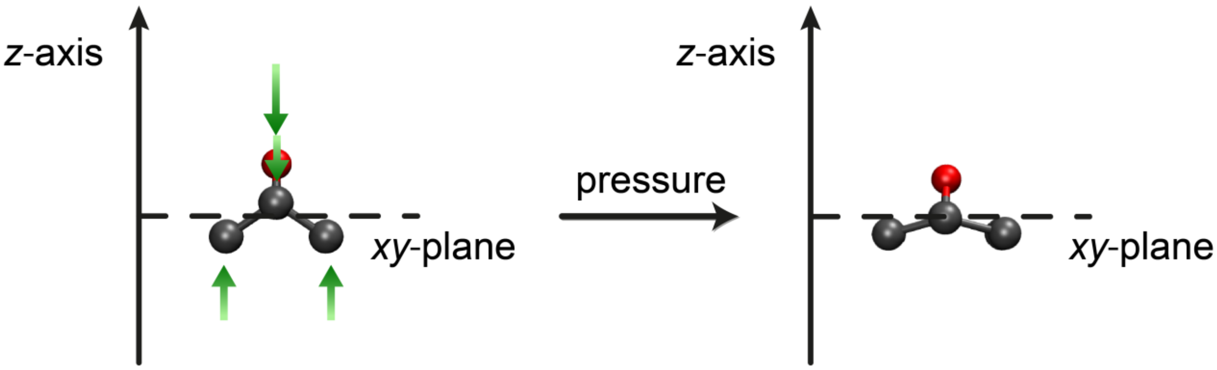

Supplementary Figure 45. Illustration of the effect of the model for simulating uniaxial pressure. The green arrows show the force acting onto the atoms. The sizes of the arrows indicate the difference in the strength of the force depending on the distance of the sphere to the *xy*-plane. On the right side, the atoms of the molecule are closer to the *xy*-plane and the molecular system is slightly deformed which is caused by the uniaxial pressure.

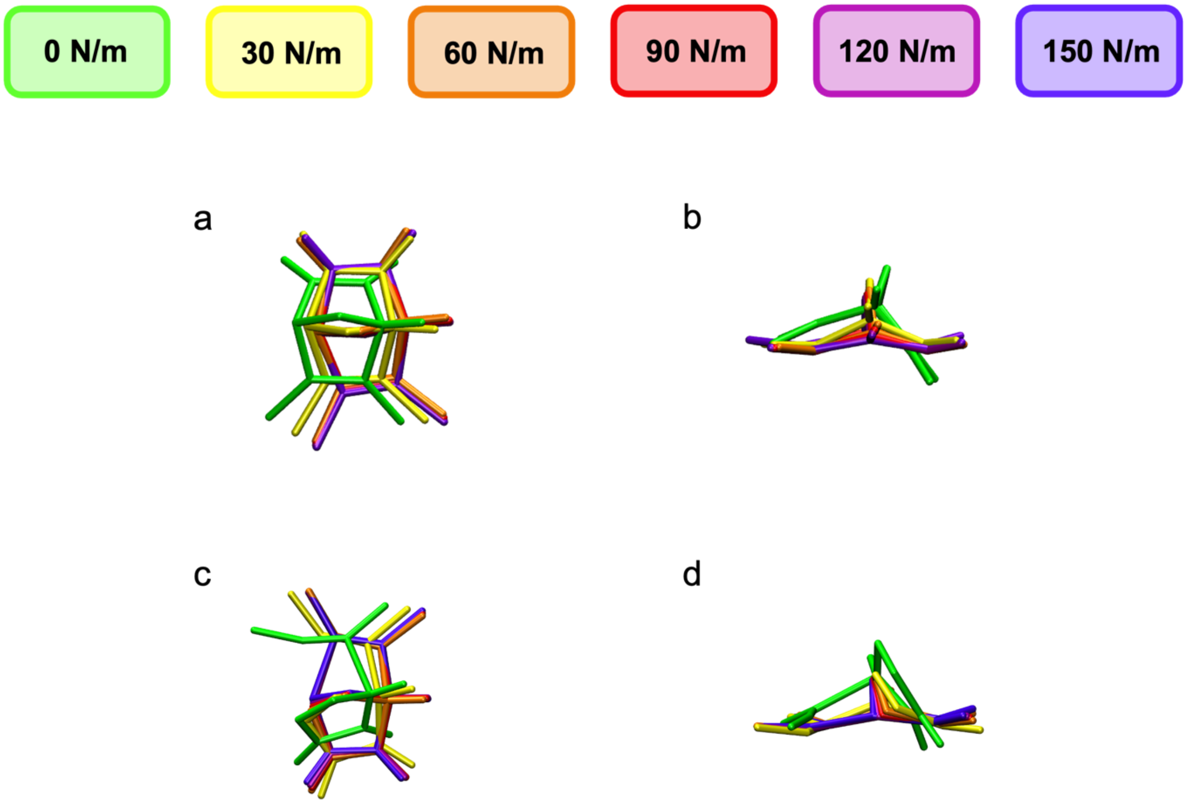

Supplementary Figure 46. Selected structures of the reactant and the transition state at various force constant to illustrate structural changes under the impact of uniaxial pressure. For simplicity, only the oxanorbornadiene-motif is shown. (**a**) top view and (**b**) side view on the reactant structures with varying uniaxial pressure. (**c**) top view and (**d**) side view on the transition state structures with varying uniaxial pressure.

5. NMR and ESI-MS spectra

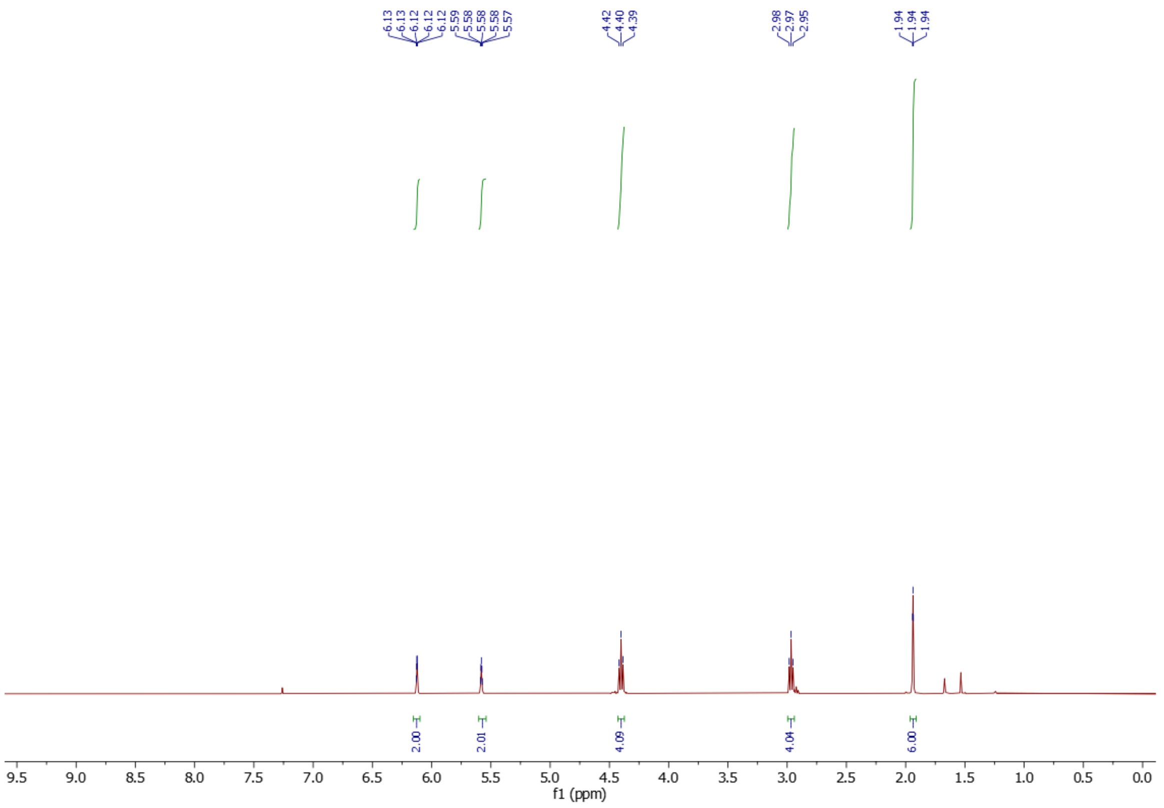

Supplementary Figure 47. ^{1}H NMR spectrum (400 MHz, $CDCl_3$) of bis(2-methacryloyl)oxyethyl disulfide (**N1-CL**).

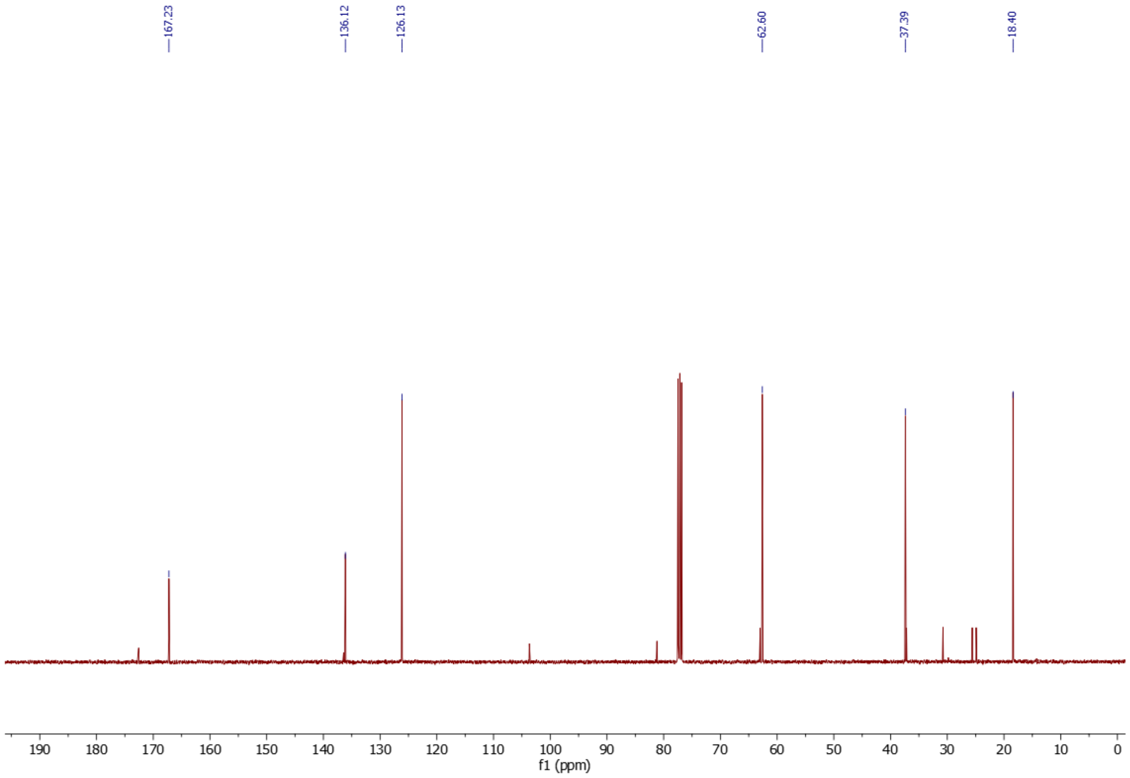

Supplementary Figure 48. ^{13}C NMR spectrum (101 MHz, $CDCl_3$) of bis(2-methacryloyl)oxyethyl disulfide (**N1-CL**).

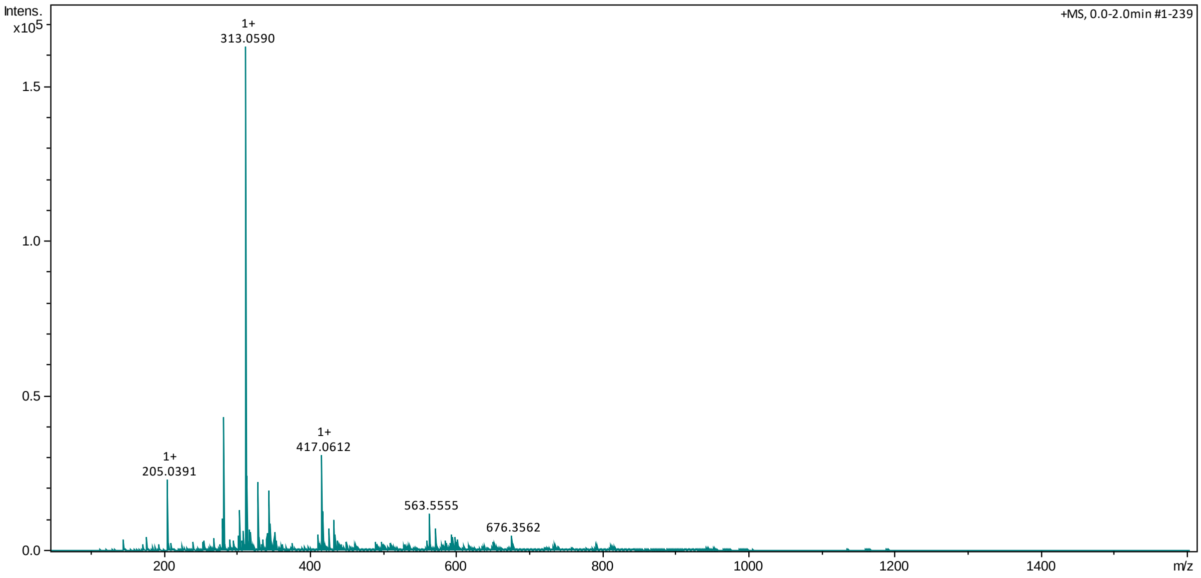

Supplementary Figure 49. ESI-MS (*m/z*) of bis(2-methacryloyl)oxyethyl disulfide (**N1-CL**).

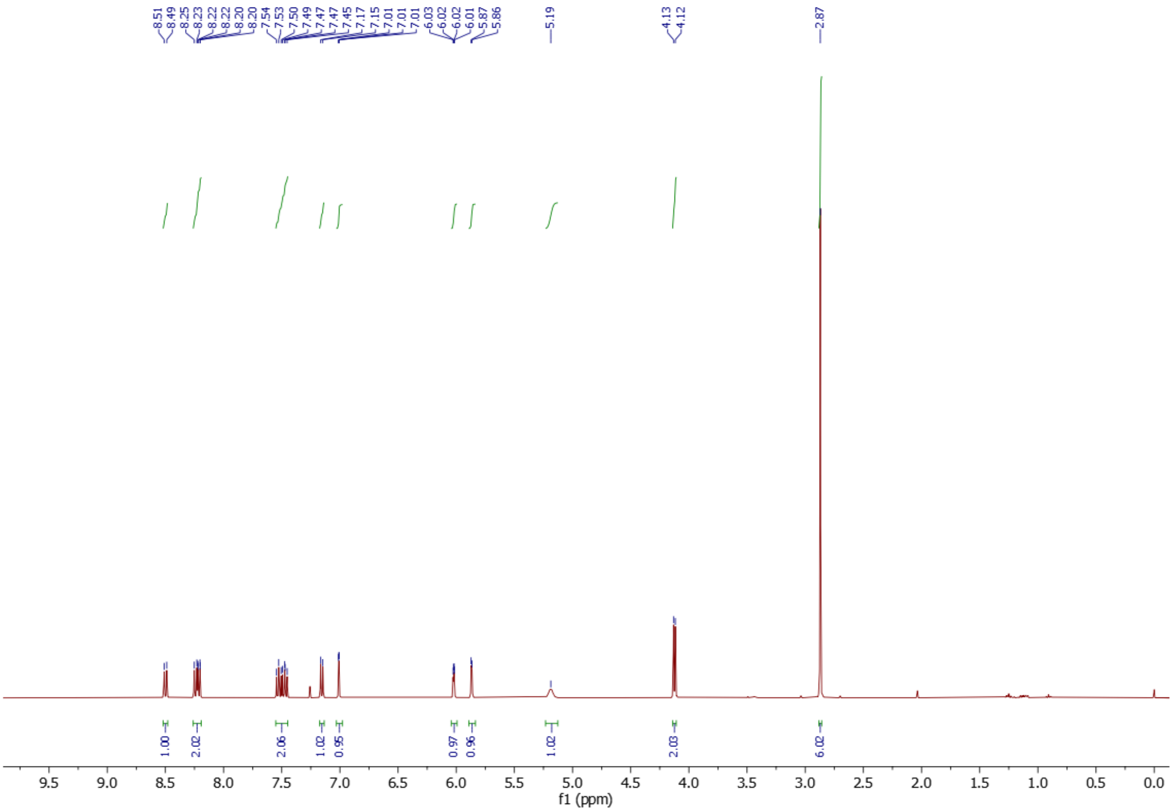

Supplementary Figure 50. ^{1}H NMR spectrum (400 MHz, $CDCl_3$) of 5-(dimethylamino)-N-(furan-2-ylmethyl)naphthalene-1-sulfonamide (**N1-A3**).

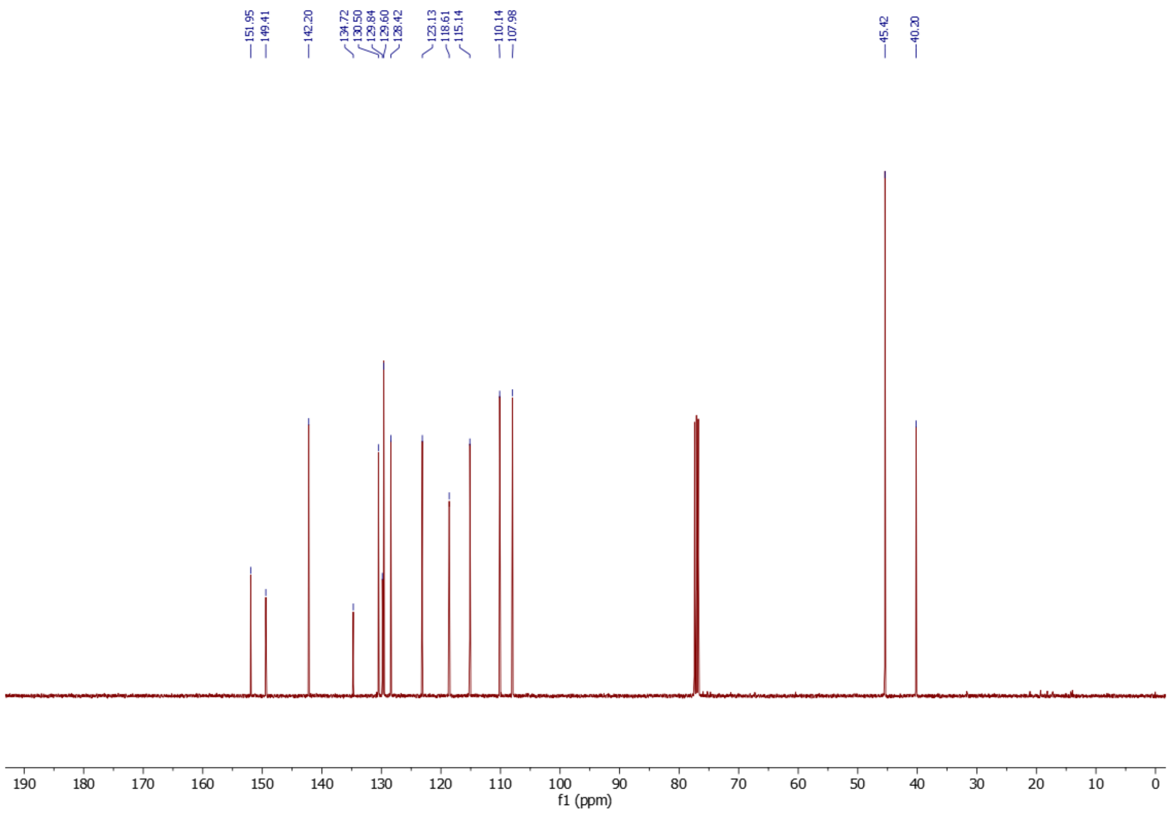

Supplementary Figure 51. ^{13}C NMR spectrum (101 MHz, $CDCl_3$) of 5-(dimethylamino)-N-(furan-2-ylmethyl)naphthalene-1-sulfonamide (**N1-A3**).

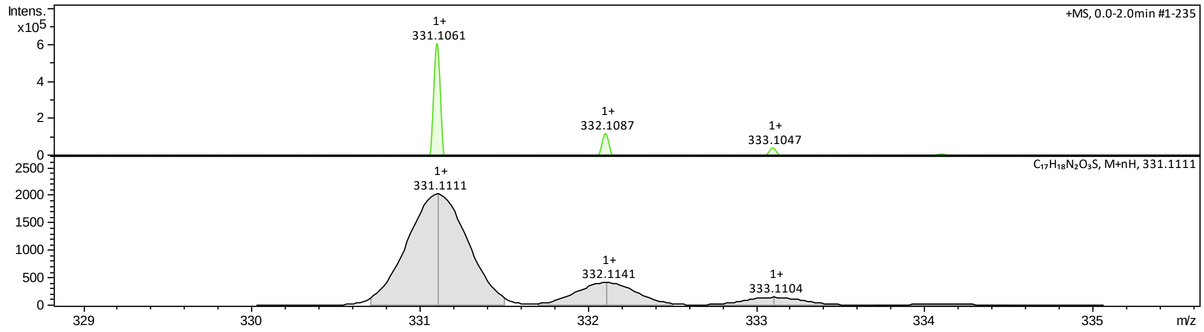

Supplementary Figure 52. ESI-MS (*m/z*) of 5-(dimethylamino)-N-(furan-2-ylmethyl)naphthalene-1-sulfonamide (**N1-A3**).

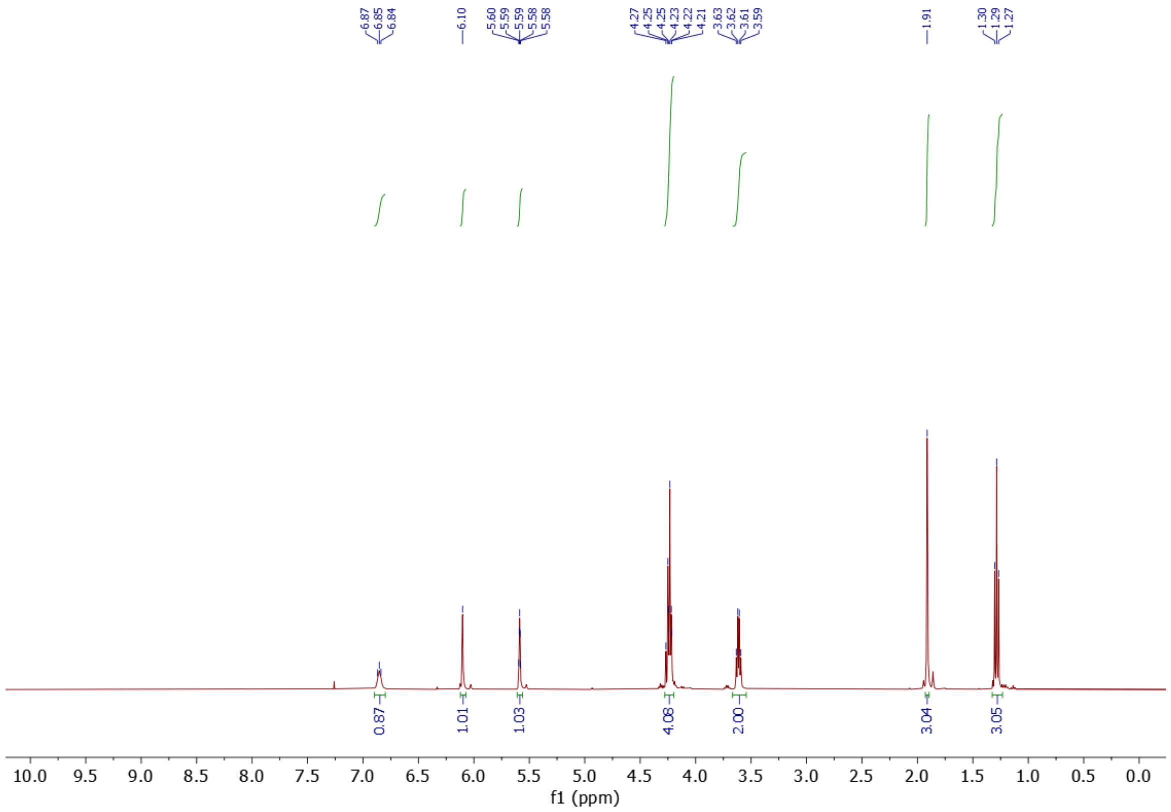

Supplementary Figure 53. ^1H NMR spectrum (400 MHz, $CDCl_3$) of Ethyl 4-((2-(methacryloyloxy)ethyl)amino)-4-oxobut-2-ynoate (**N1-B3**).

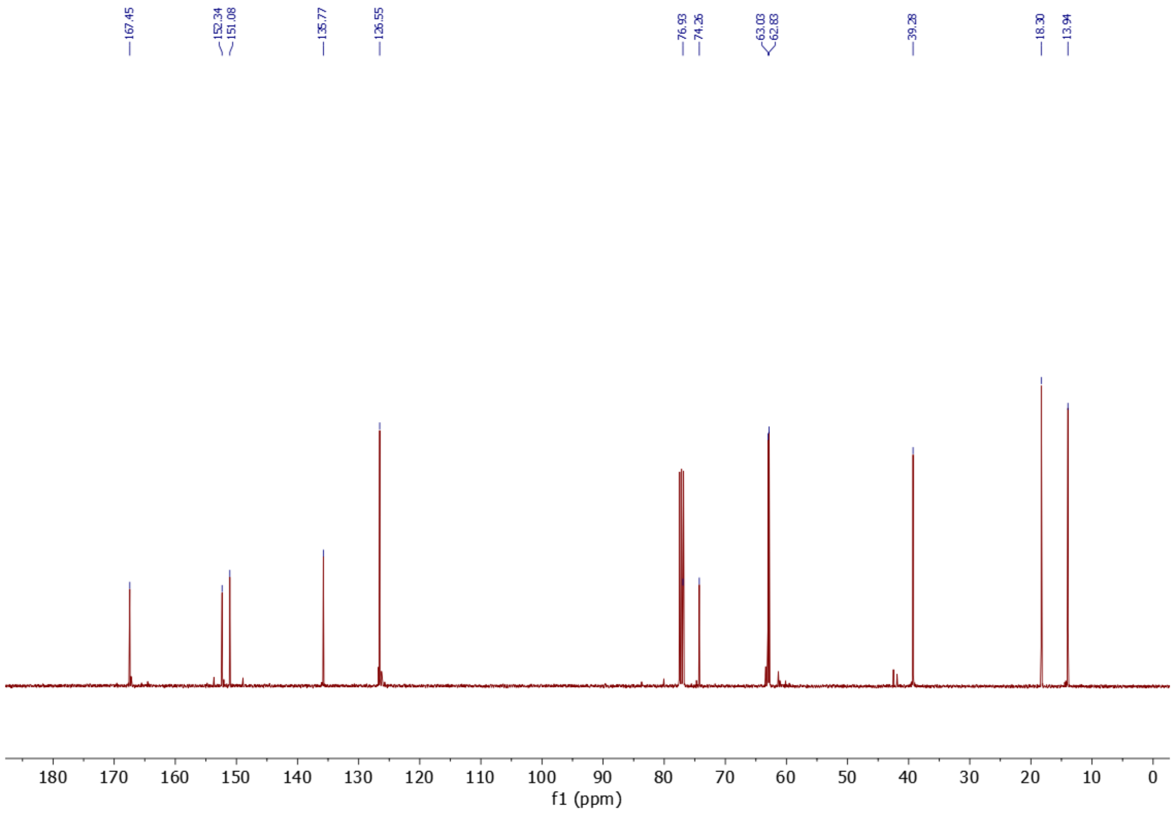

Supplementary Figure 54. ^{13}C NMR spectrum (101 MHz, $CDCl_3$) of Ethyl 4-((2-(methacryloyloxy)ethyl)amino)-4-oxobut-2-ynoate (**N1-B3**).

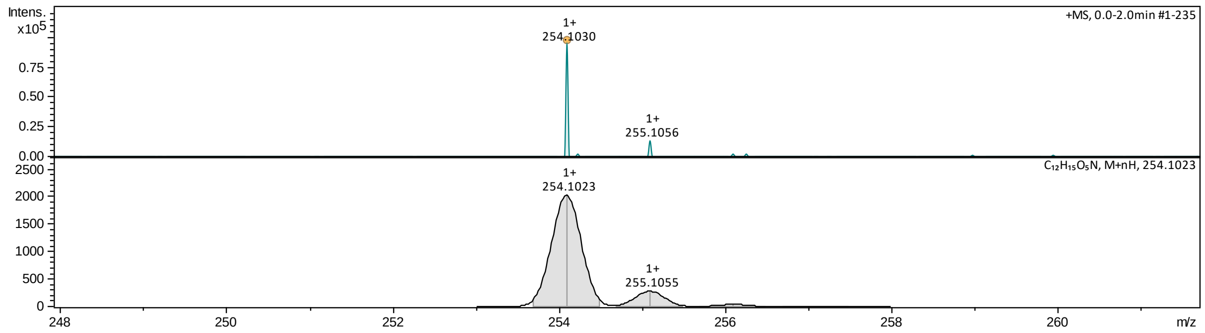

Supplementary Figure 55. ESI-MS (*m/z*) of Ethyl 4-((2-(methacryloyloxy)ethyl)amino)-4-oxobut-2-ynoate (**N1-B3**).

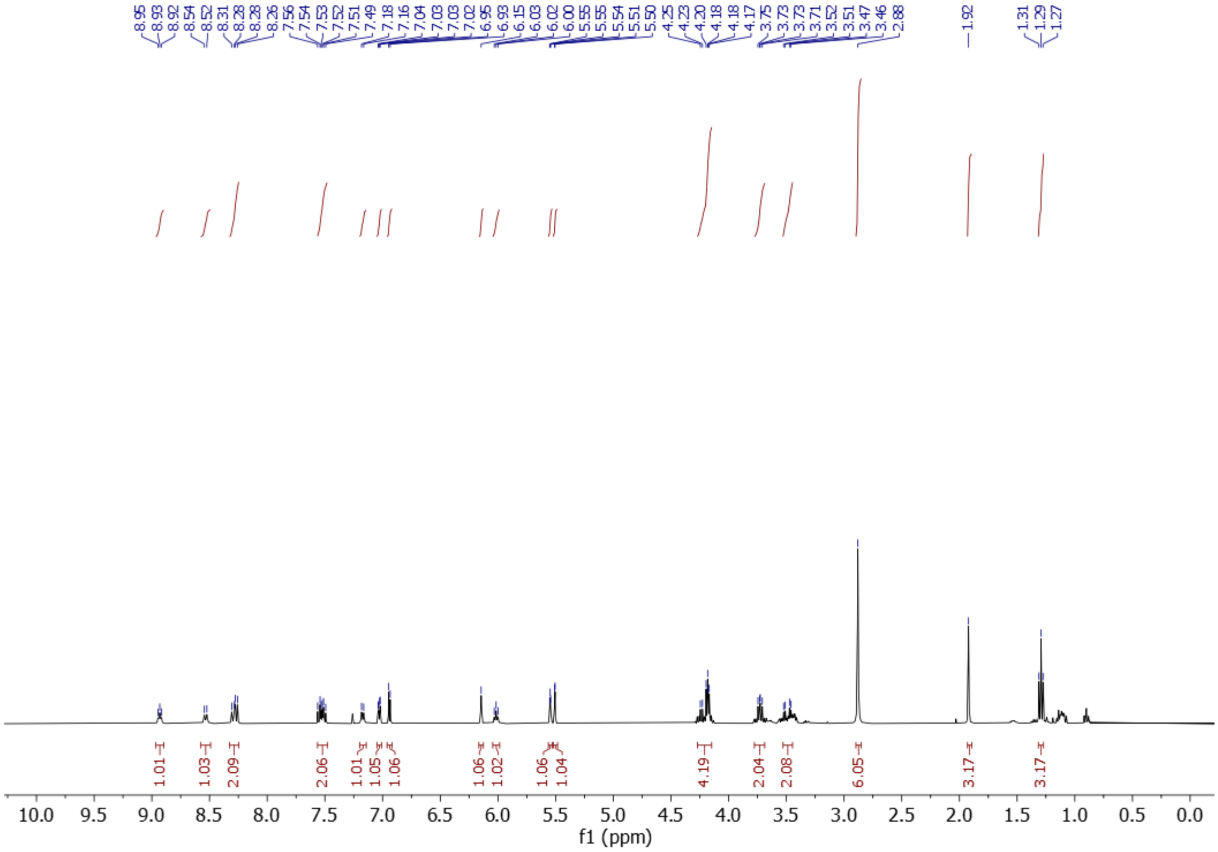

Supplementary Figure 56. ^{1}H NMR spectrum (400 MHz, $CDCl_3$) of ethyl 1-(((5-(dimethylamino)naphthalene)-1-sulfonamido)methyl)-3-((2 (methacryloyloxy)ethyl)carbamoyl)-7-oxabicyclo[2.2.1]hepta-2,5-diene-2-carboxylate (**N1-DA1**).

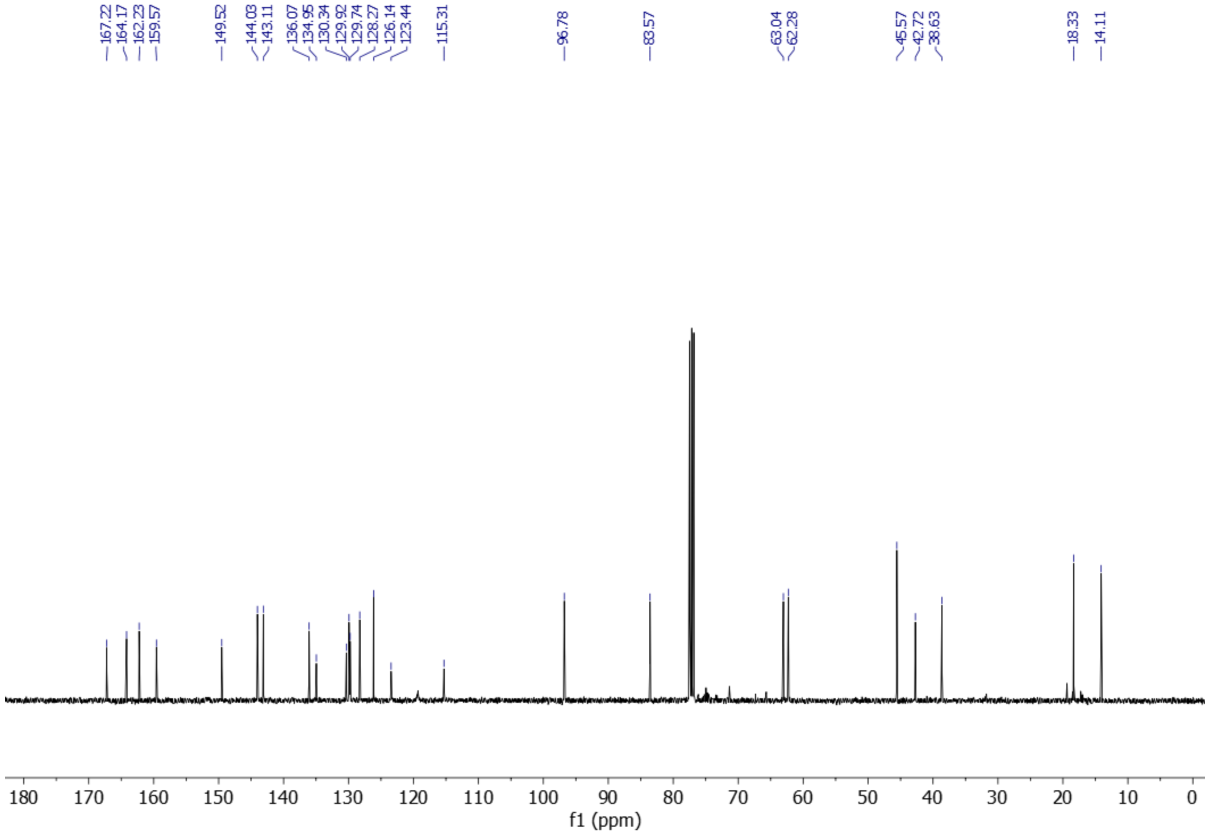

Supplementary Figure 57. ^{13}C NMR spectrum (101 MHz, $CDCl_3$) of ethyl 1-(((5-(dimethylamino)naphthalene)-1-sulfonamido)methyl)-3-((2 (methacryloyloxy)ethyl)carbamoyl)-7-oxabicyclo[2.2.1]hepta-2,5-diene-2-carboxylate (**N1-DA1**).

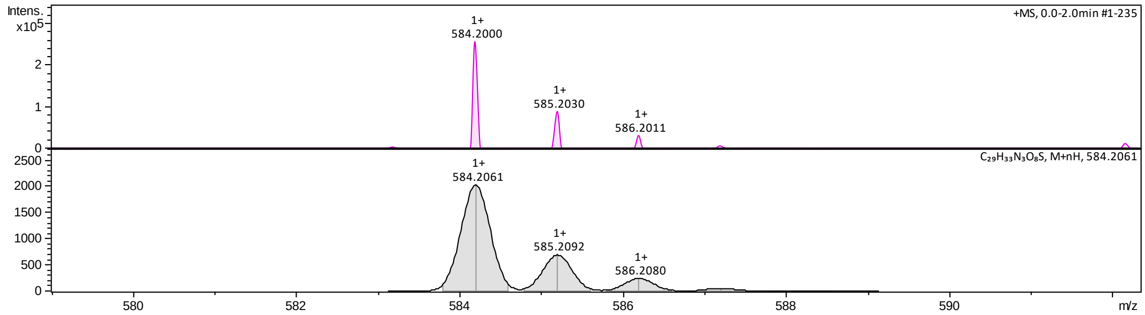

Supplementary Figure 58. ESI-MS (*m/z*) of ethyl 1-(((5-(dimethylamino)naphthalene)-1-sulfonamido)methyl)-3-((2 (methacryloyloxy)ethyl)carbamoyl)-7-oxabicyclo[2.2.1]hepta-2,5-diene-2-carboxylate (**N1-DA1**).

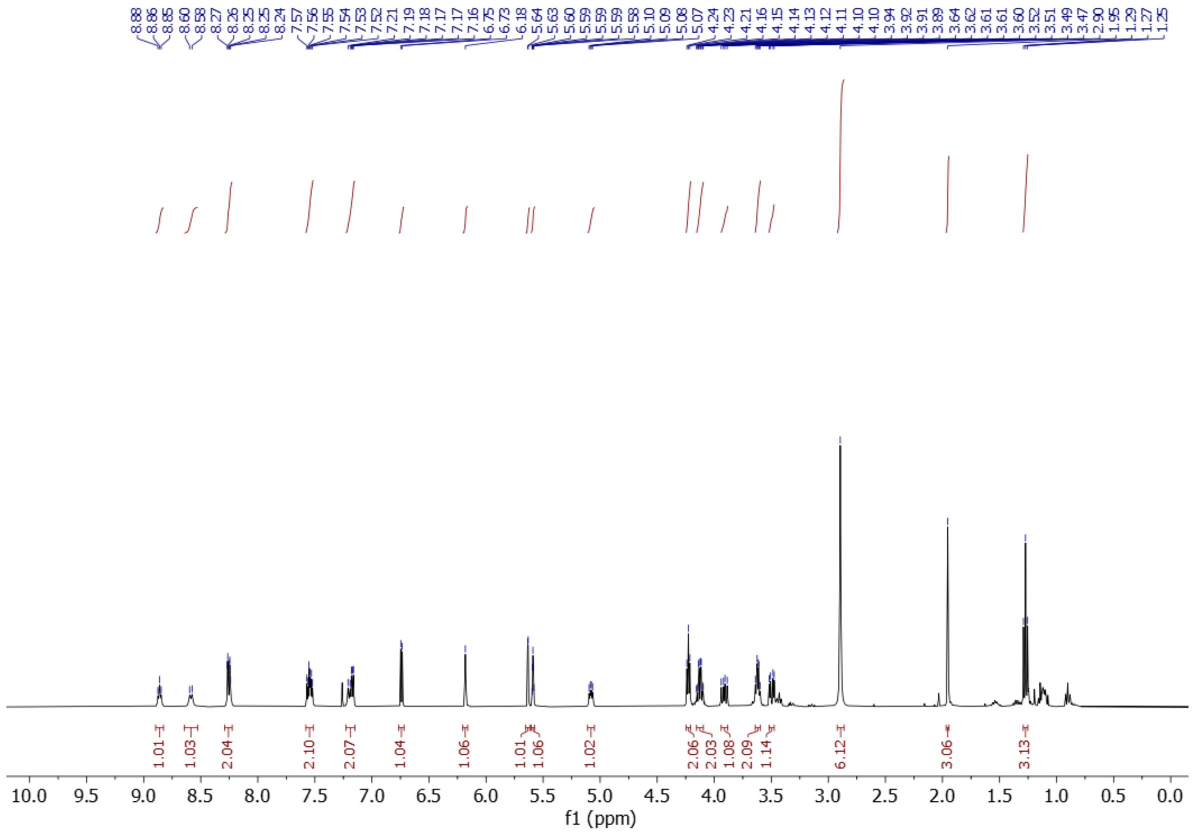

Supplementary Figure 59. ^{1}H NMR spectrum (400 MHz, $CDCl_3$) of ethyl 1-(((5-(dimethylamino)naphthalene)-1-sulfonamido)methyl)-3-((2 (methacryloyloxy)ethyl)carbamoyl)-7-oxabicyclo[2.2.1]hepta-2,5-diene-2-carboxylate (**N1-DA2**).

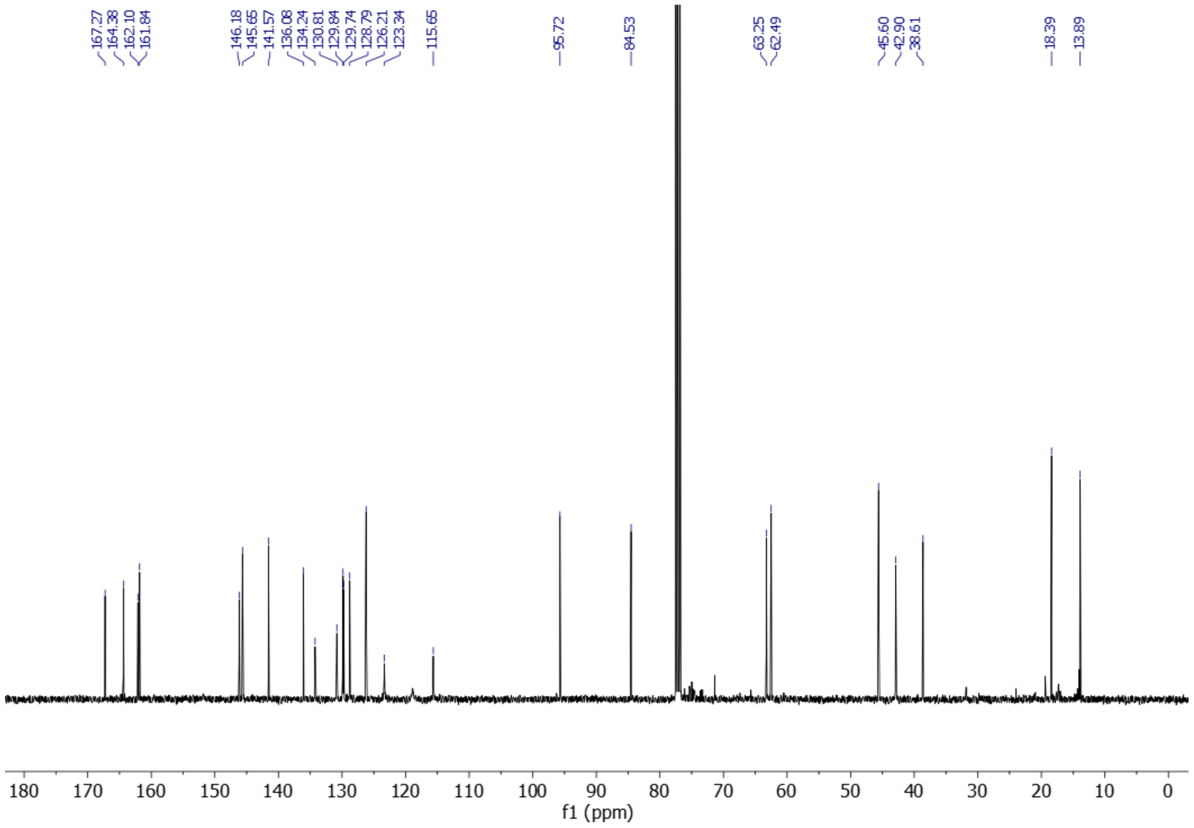

Supplementary Figure 60. ^{13}C NMR spectrum (101 MHz, $CDCl_3$) of ethyl 1-(((5-(dimethylamino)naphthalene)-1-sulfonamido)methyl)-3-((2 (methacryloyloxy)ethyl)carbamoyl)-7-oxabicyclo[2.2.1]hepta-2,5-diene-2-carboxylate (**N1-DA2**).

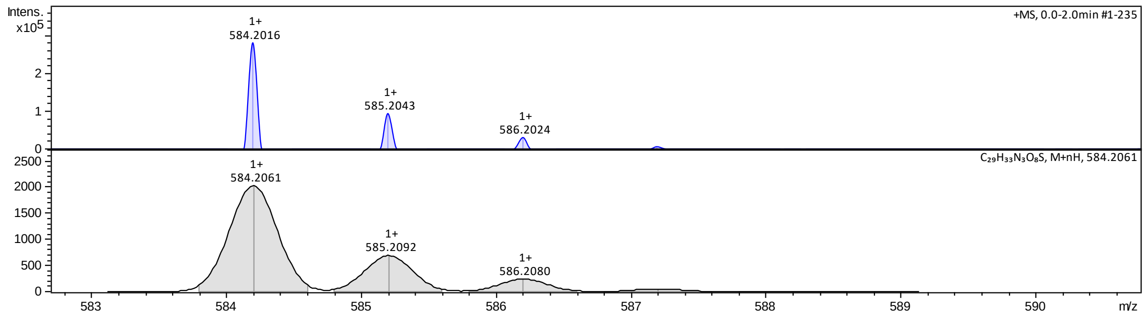

Supplementary Figure 61. ESI-MS (*m/z*) of ethyl 1-(((5-(dimethylamino)naphthalene)-1-sulfonamido)methyl)-3-((2 (methacryloyloxy)ethyl)carbamoyl)-7-oxabicyclo[2.2.1]hepta-2,5-diene-2-carboxylate (**N1-DA2**).

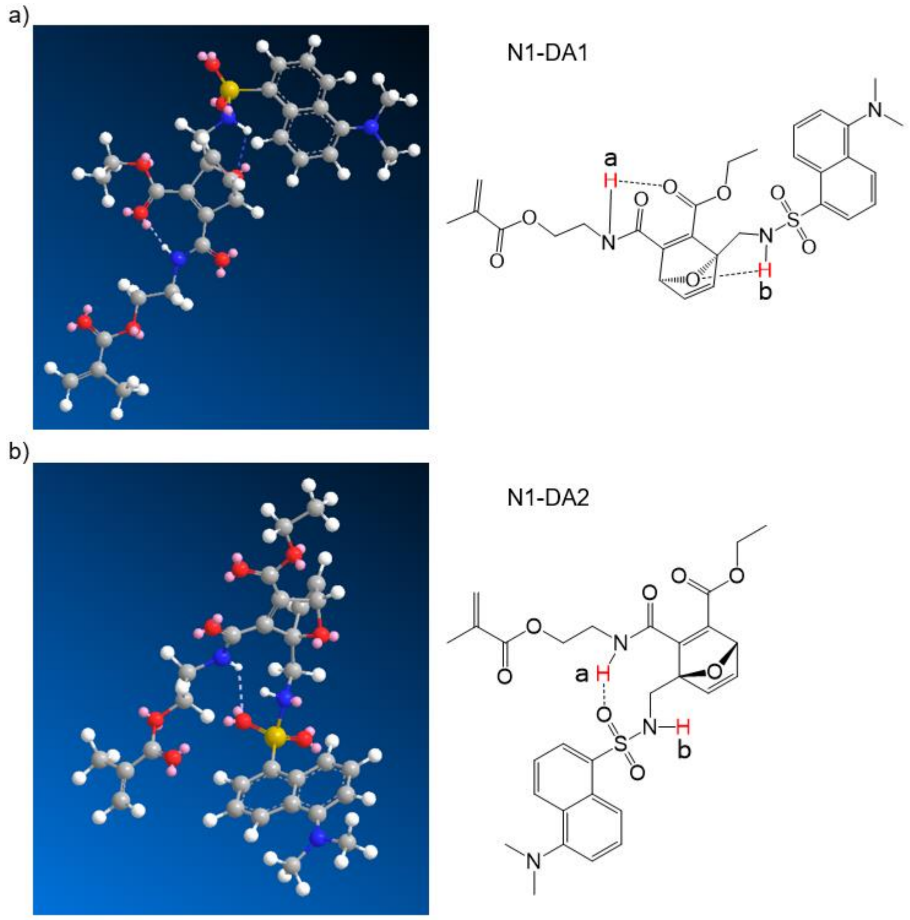

Supplementary Figure 62. 3D structure of (**a**) **N1-DA1** and (**b**) **N1-DA2** (The hydrogen bonds in **N1-DA1** and **N1-DA2** were calculated by Chem3D®, version: 19.0.1.28).

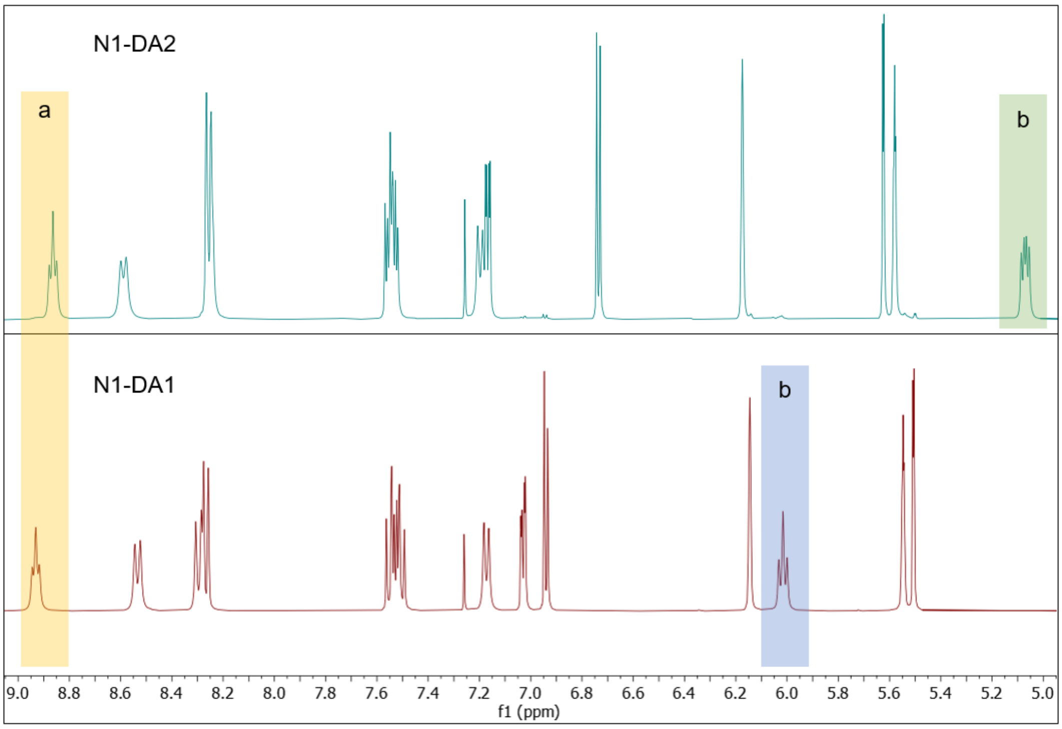

Supplementary Figure 63. ^1H NMR (400 MHz, $CDCl_3$) stack of **N1-DA1** and **N1-DA2**.

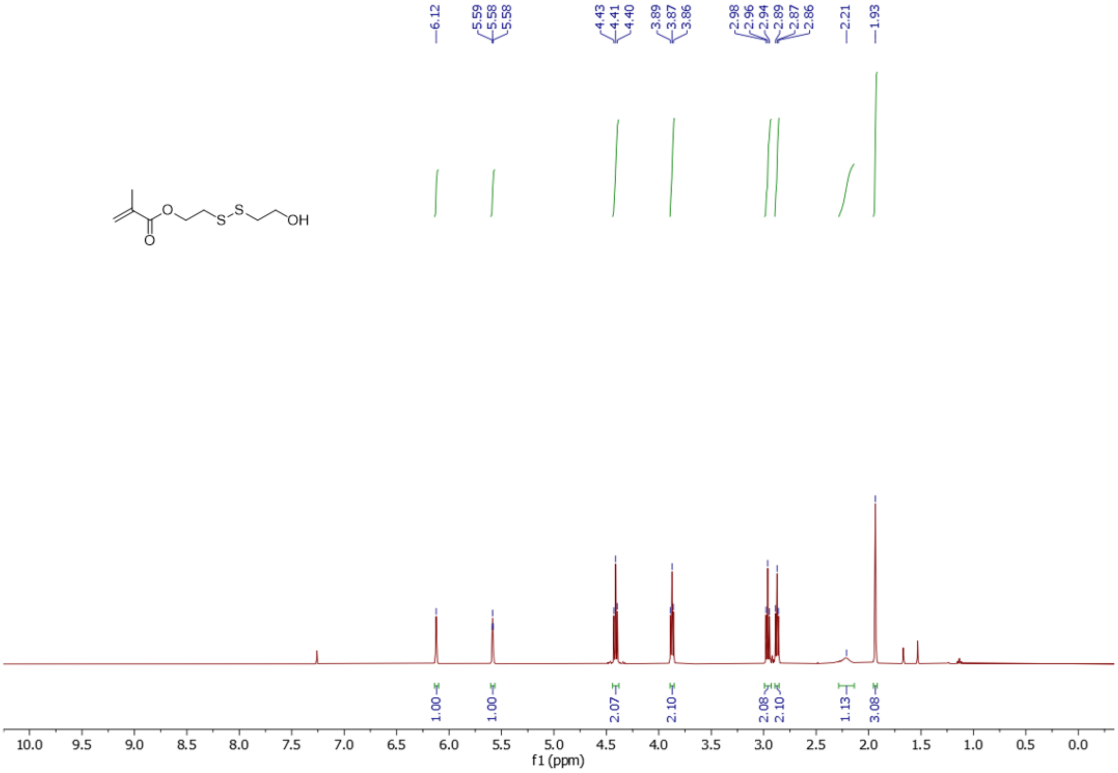

Supplementary Figure 64. ^1H NMR spectrum (400 MHz, $CDCl_3$) of 2-((2-hydroxyethyl)disulfaneyl)ethyl methacrylate (**N2-A2**).

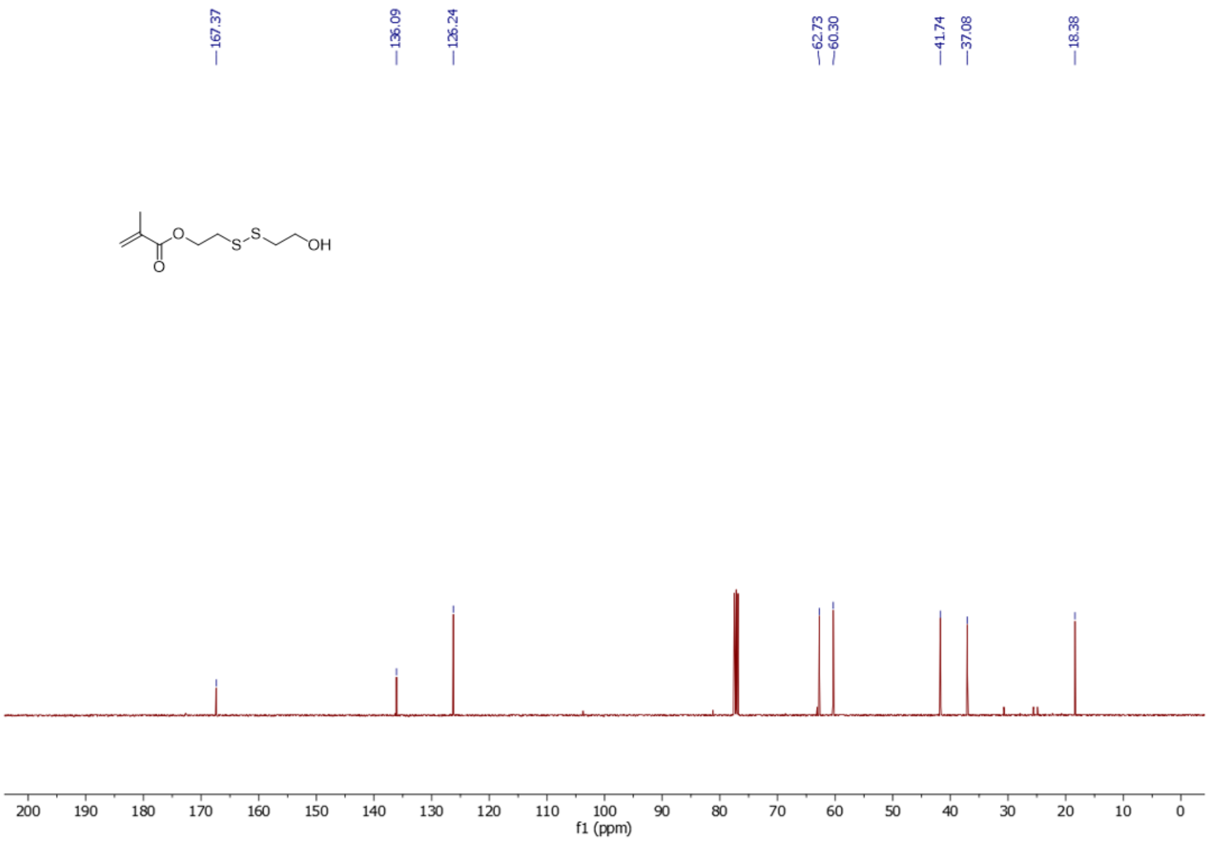

Supplementary Figure 65. ^{13}C NMR spectrum (101 MHz, $CDCl_3$) of 2-((2-hydroxyethyl)disulfaneyl)ethyl methacrylate (**N2-A2**).

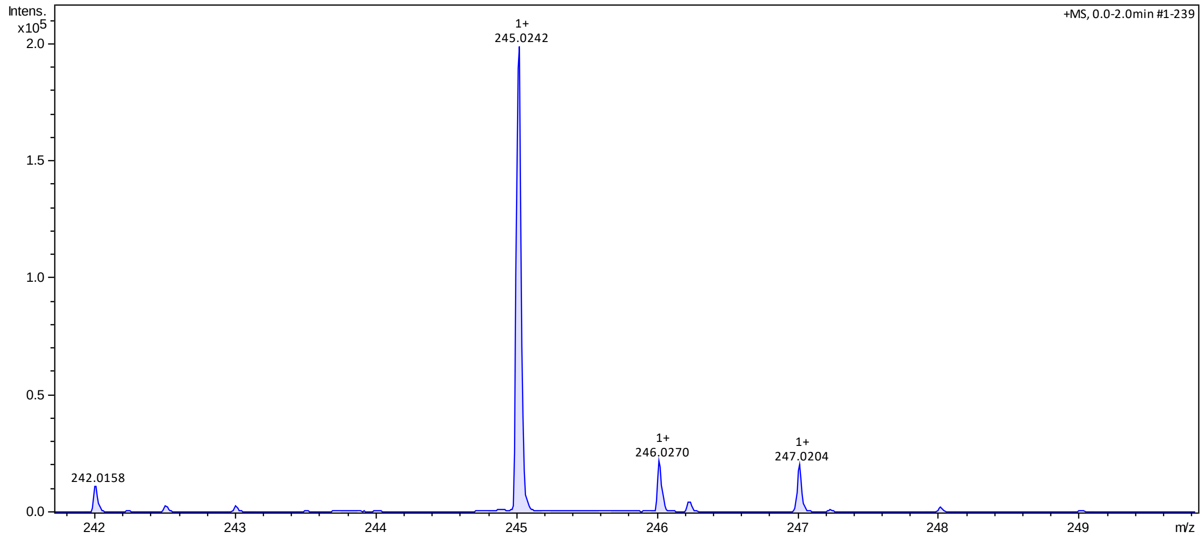

Supplementary Figure 66. ESI-MS (*m*/*z*) of 2-((2-hydroxyethyl)disulfaneyl)ethyl methacrylate (**N2-A2**).

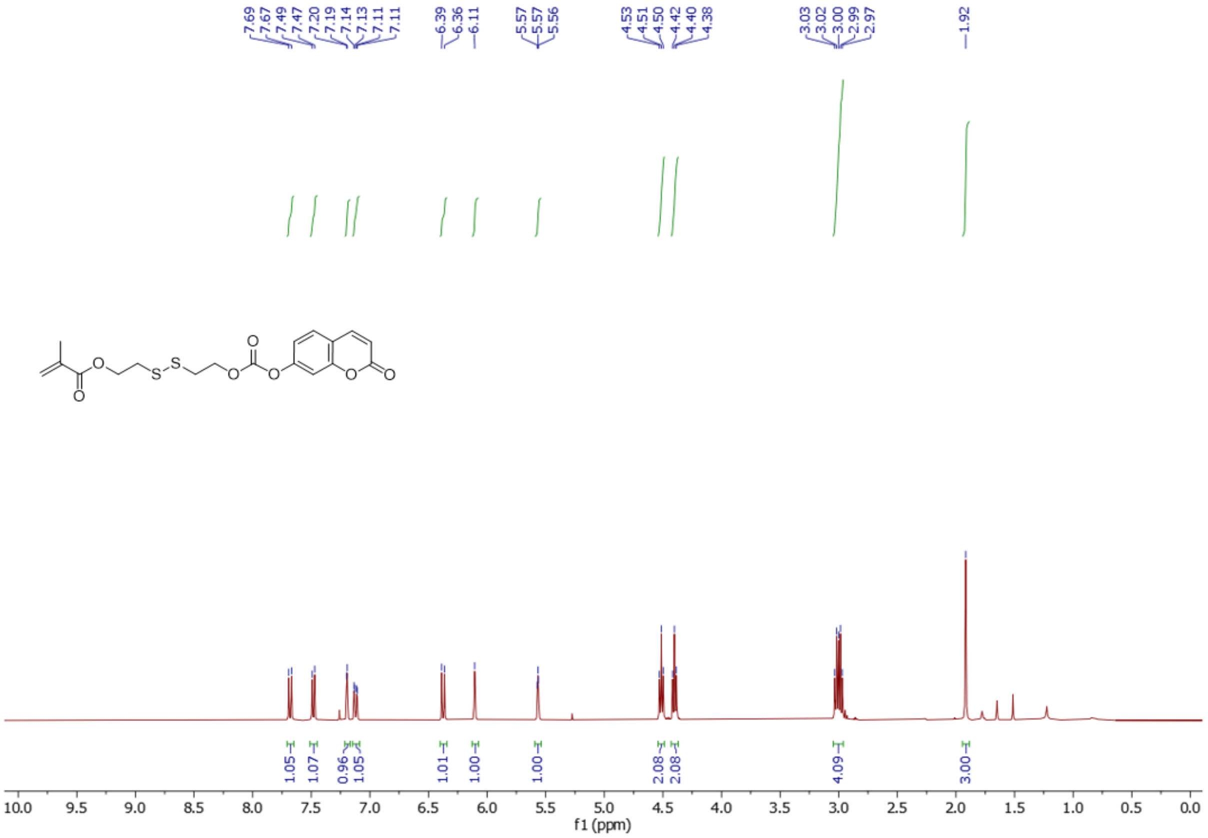

Supplementary Figure 67. ^{1}H NMR spectrum (400 MHz, $CDCl_3$) of 2-((2-((((2-oxo-2H-chromen-7-yl)oxy)carbonyl)oxy)ethyl) disulfaneyl)ethyl methacrylate (**N2-A3**).

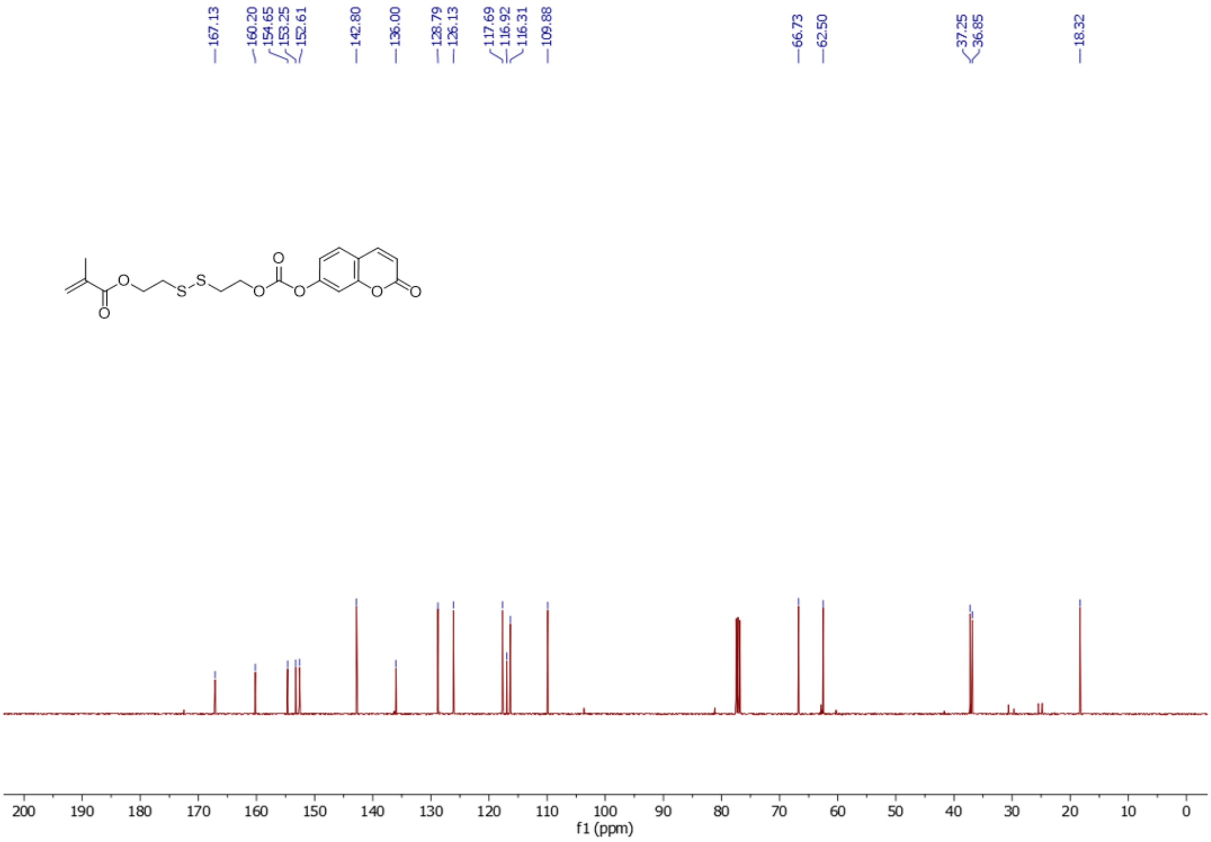

Supplementary Figure 68. ^{13}C NMR spectrum (101 MHz, $CDCl_3$) of 2-((2-((((2-oxo-2H-chromen-7-yl)oxy)carbonyl)oxy)ethyl) disulfaneyl)ethyl methacrylate (**N2-A3**).

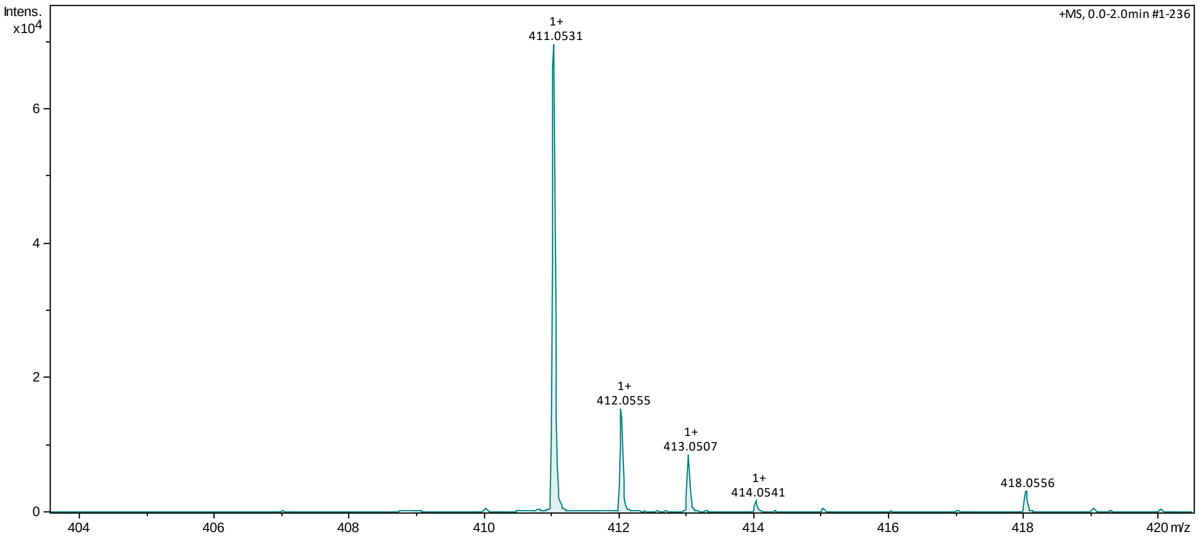

Supplementary Figure 69. ESI-MS (*m/z*) of 2-((2-((((2-oxo-2H-chromen-7-yl)oxy)carbonyl)oxy)ethyl) disulfaneyl)ethyl methacrylate (**N2-A3**).

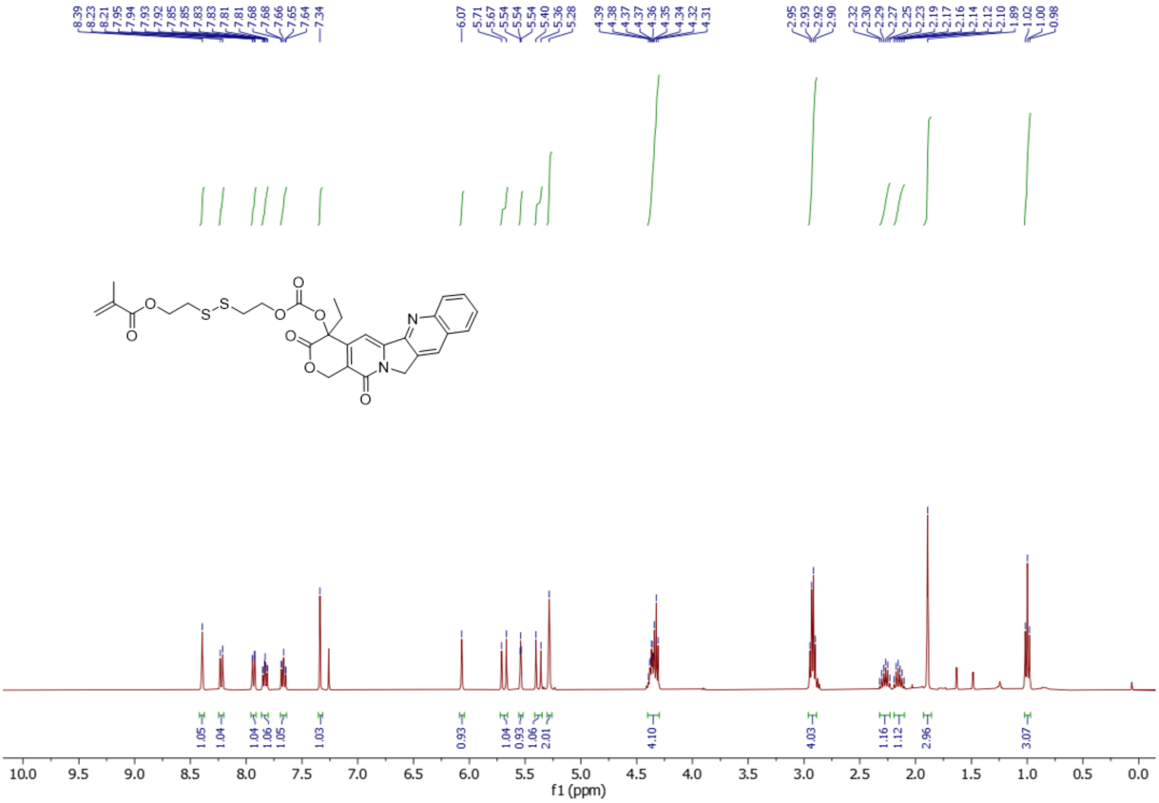

Supplementary Figure 70. ^{1}H NMR spectrum (400 MHz, $CDCl_3$) of 2-((2-((((4-ethyl-3,14-dioxo-3,4,12,14-tetrahydro-1H pyrano[3',4':6,7] indolizino[1,2-b]quinolin-4-yl)oxy)carbonyl)oxy)ethyl)disulfaneyl)ethyl methacrylate (**N2-B3**).

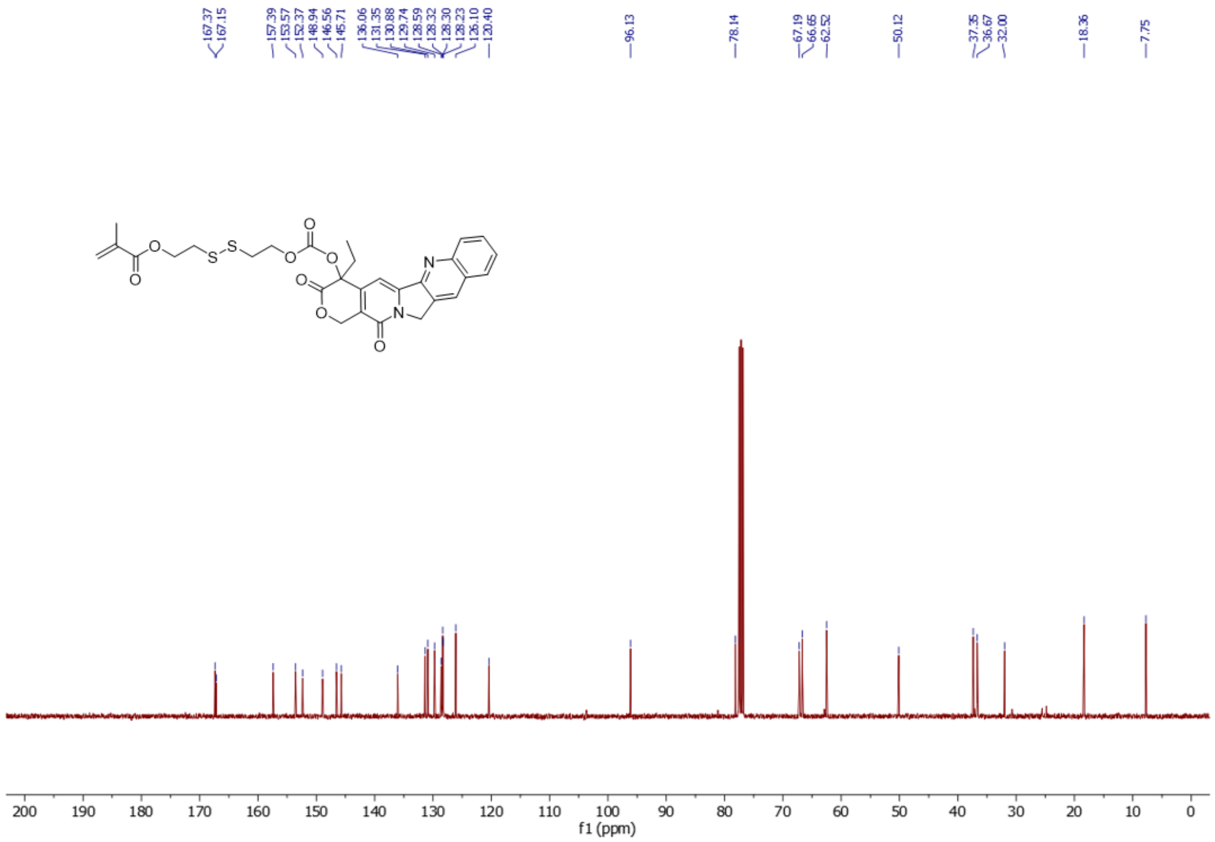

Supplementary Figure 71. ^{13}C NMR spectrum (101 MHz, $CDCl_3$) of 2-((2-((((4-ethyl-3,14-dioxo-3,4,12,14-tetrahydro-1H pyrano[3',4':6,7] indolizino[1,2-b]quinolin-4-yl)oxy)carbonyl)oxy)ethyl)disulfaneyl)ethyl methacrylate (**N2-B3**).

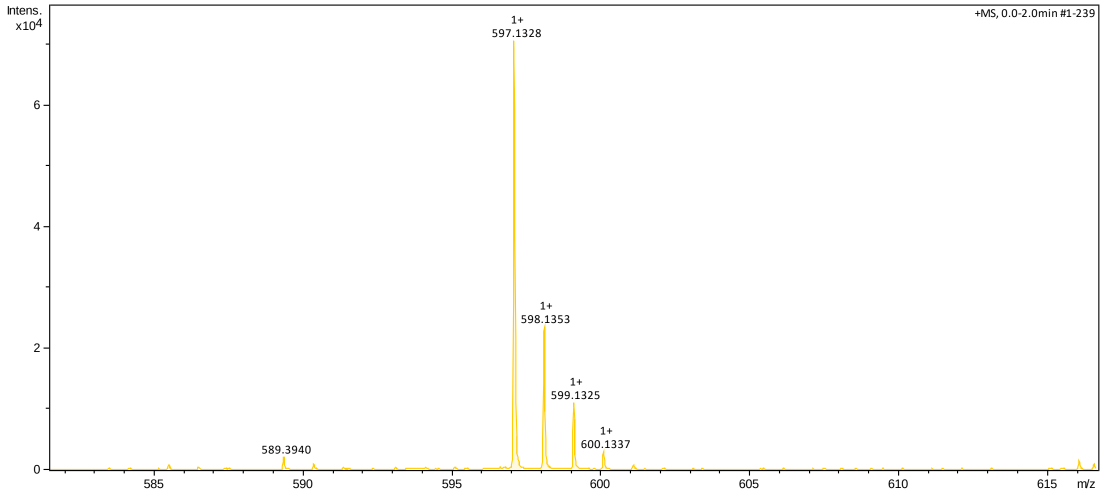

Supplementary Figure 72. ESI-MS (*m/z*) of 2-((2-((((4-ethyl-3,14-dioxo-3,4,12,14-tetrahydro-1H pyrano[3',4':6,7] indolizino[1,2-b]quinolin-4-yl)oxy)carbonyl)oxy)ethyl)disulfaneyl)ethyl methacrylate (**N2-B3**).

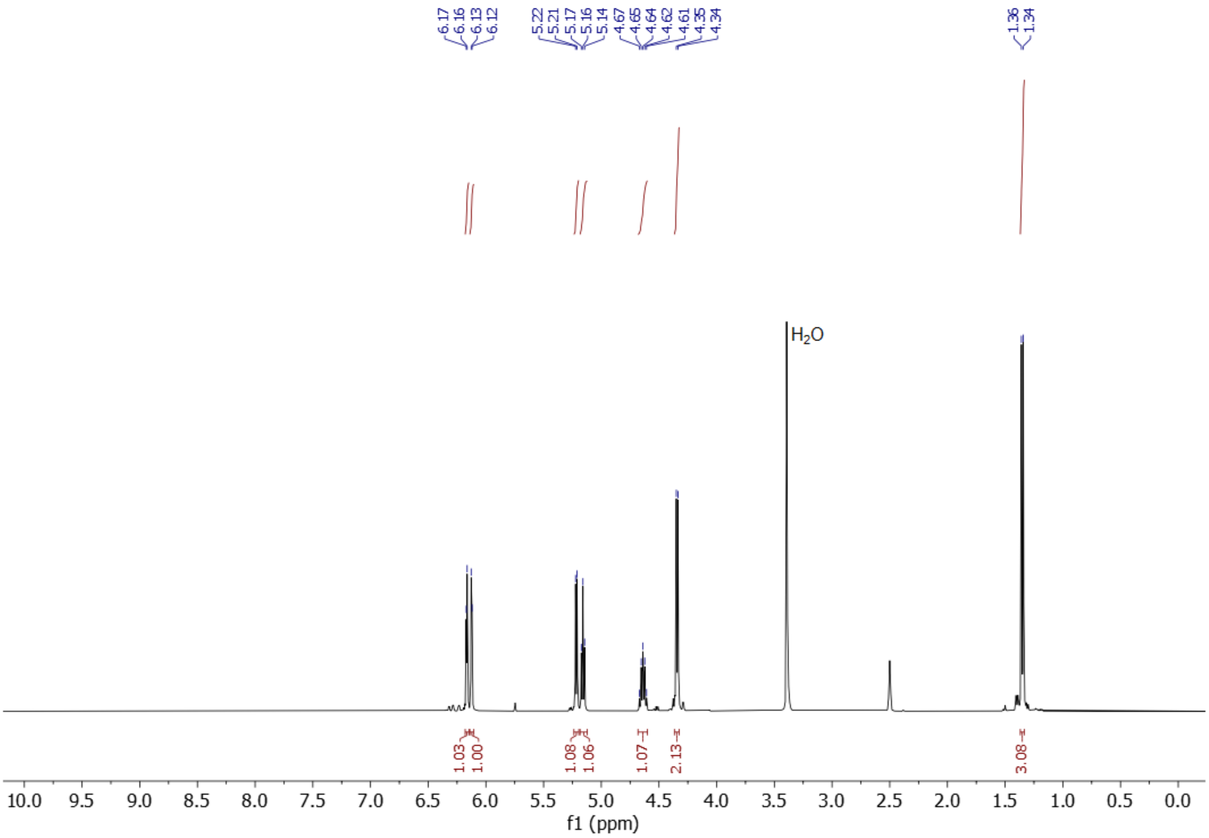

Supplementary Figure 73. ^{1}H NMR spectrum (400 MHz, $(CD_3)_2SO$) of 1-(5-(hydroxymethyl)furan-2-yl)ethan-1-ol (**N3-A2**).

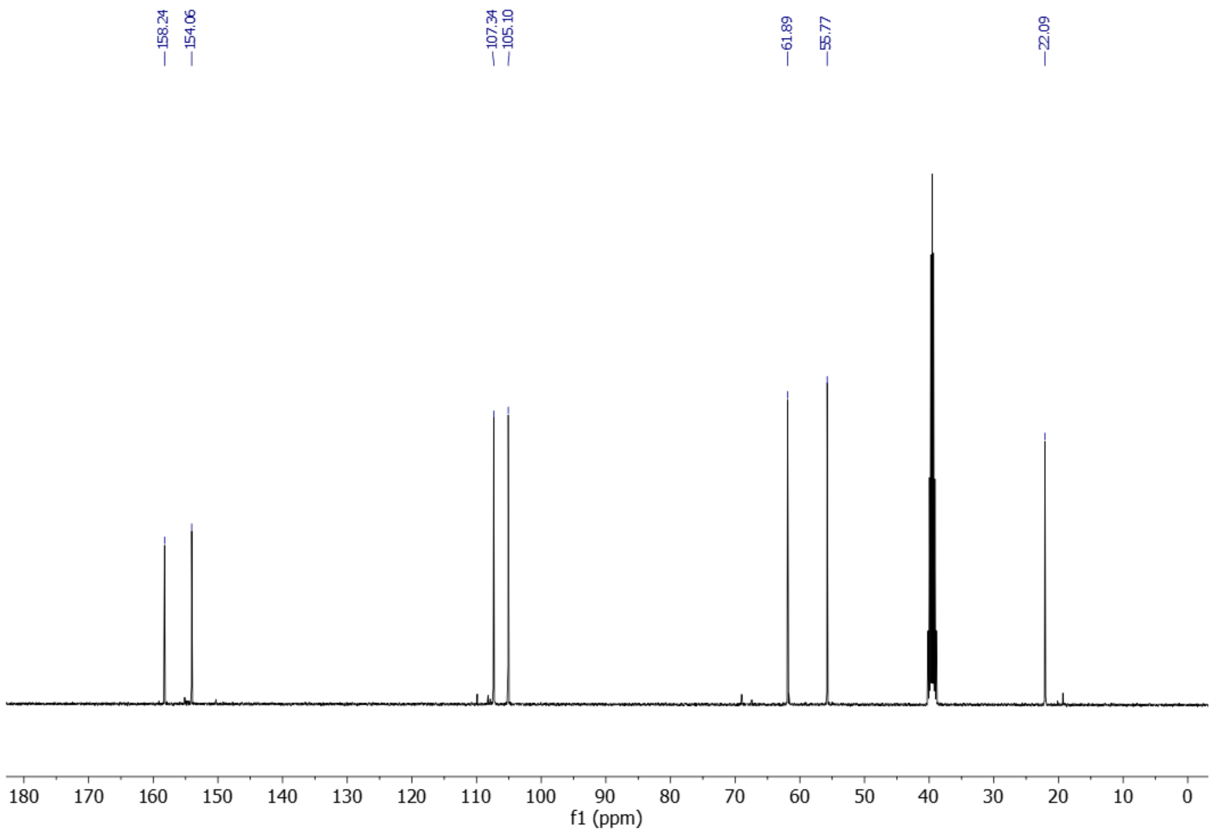

Supplementary Figure 74. ^{13}C NMR spectrum (101 MHz, $(CD_3)_2SO$) of 1-(5-(hydroxymethyl)furan-2-yl)ethan-1-ol (**N3-A2**).

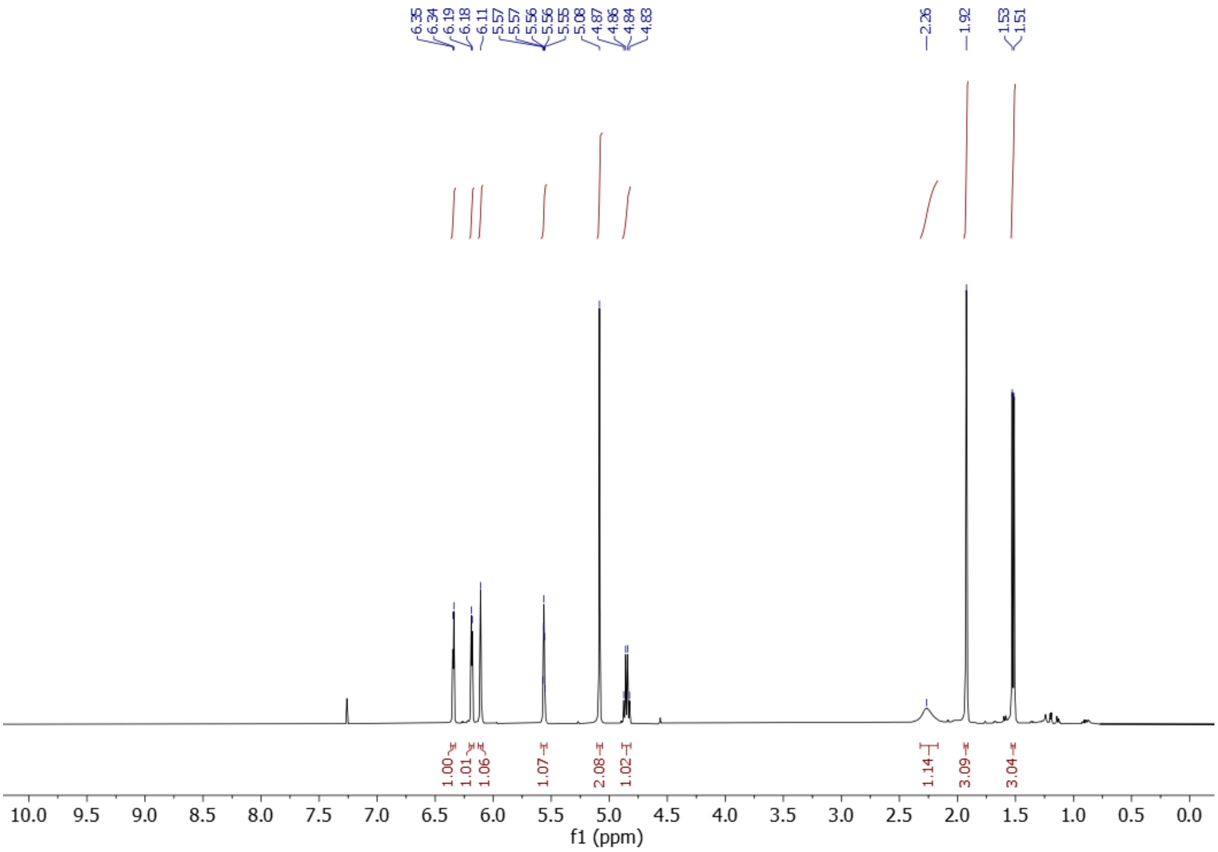

Supplementary Figure 75. ^{1}H NMR spectrum (400 MHz, $CDCl_3$) of (5-(1-hydroxyethyl)furan-2-yl)methyl methacrylate (**N3-A3**).

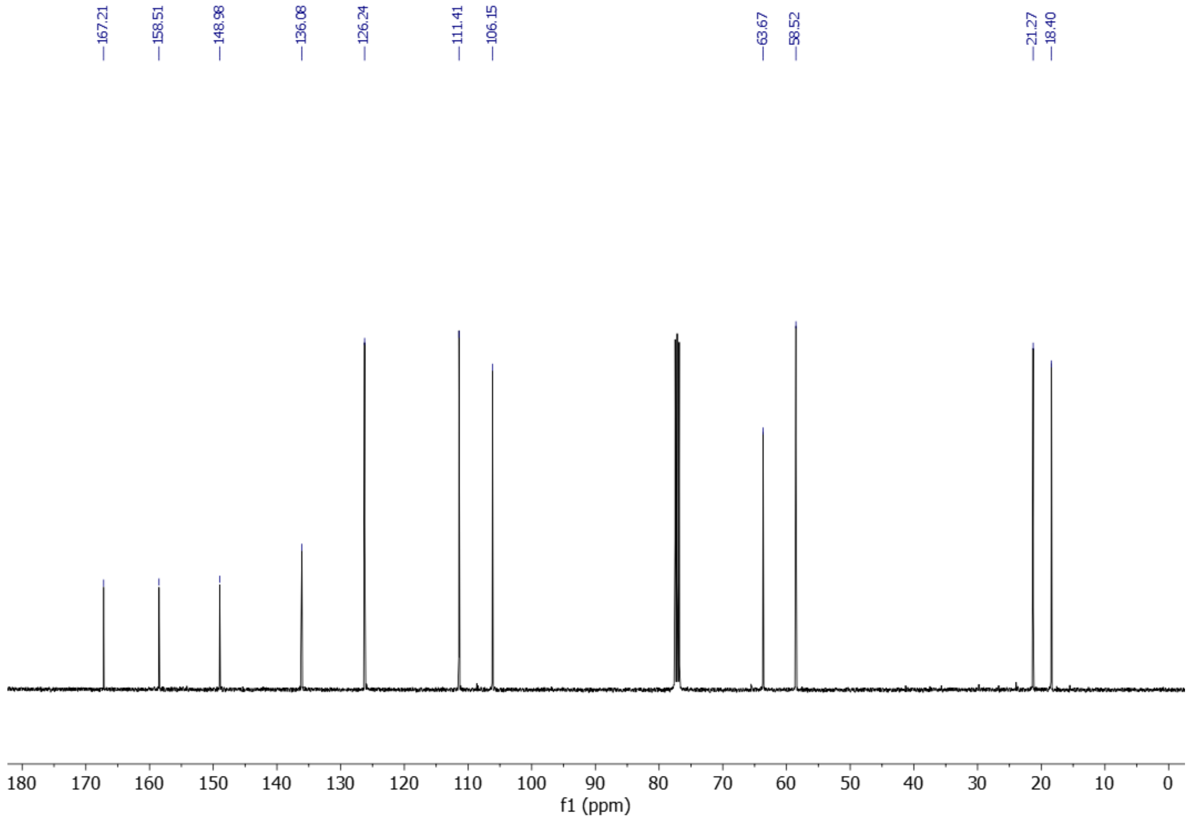

Supplementary Figure 76. ^{13}C NMR spectrum (101 MHz, $CDCl_3$) of (5-(1-hydroxyethyl)furan-2-yl)methyl methacrylate (**N3-A3**).

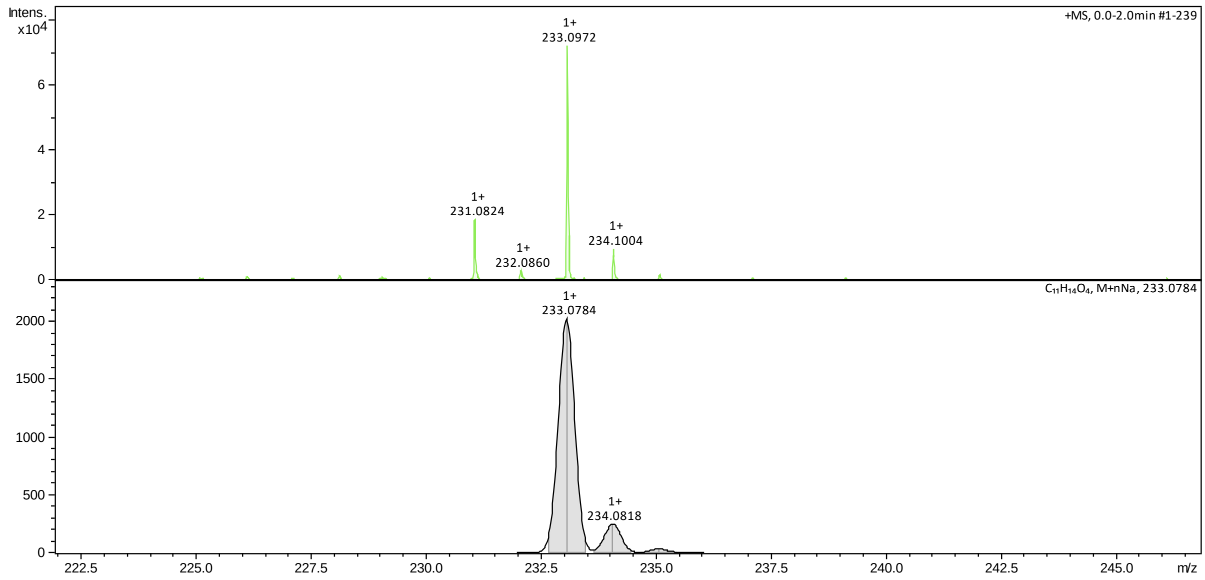

Supplementary Figure 77. ESI-MS (*m/z*) of (5-(1-hydroxyethyl)furan-2-yl)methyl methacrylate (**N3-A3**).

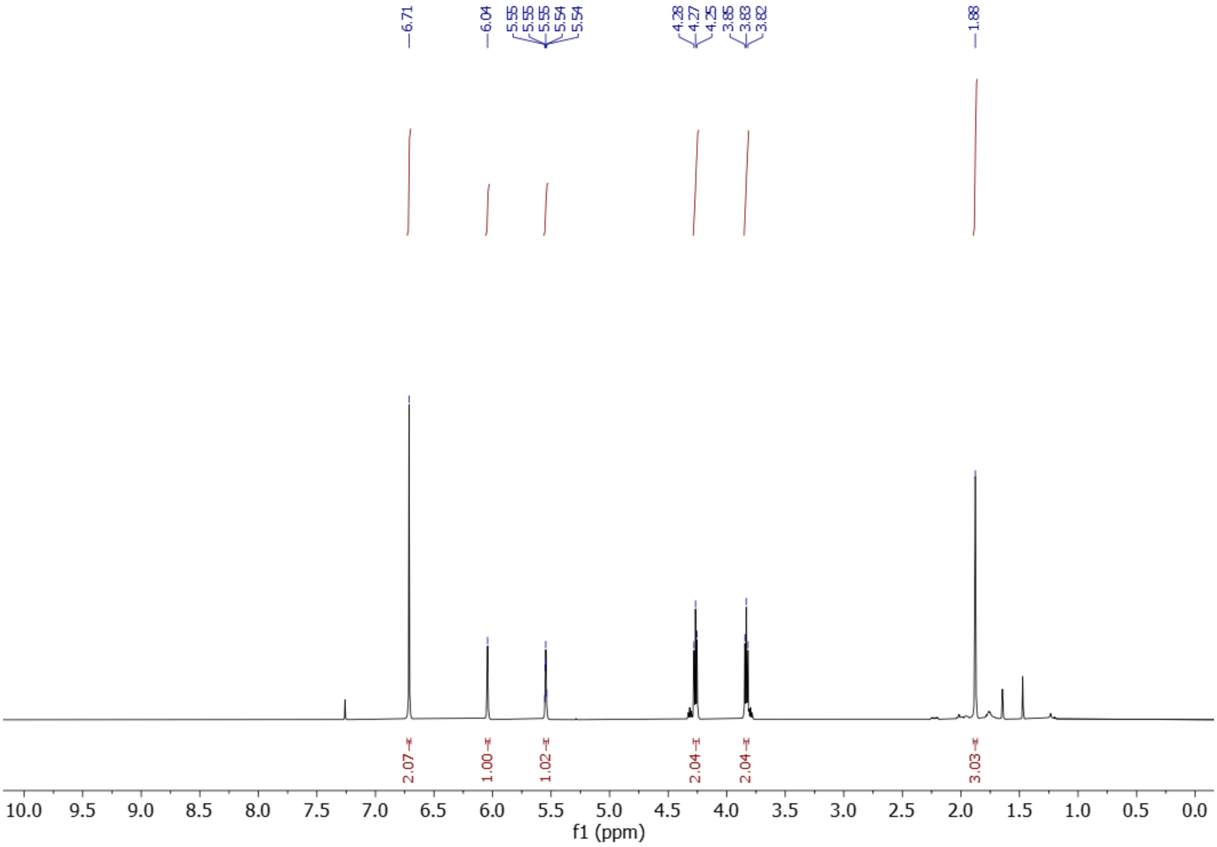

Supplementary Figure 78. ^{1}H NMR spectrum (400 MHz, $CDCl_3$) of 2-(2,5-dioxo-2,5-dihydro-1H-pyrrol-1-yl)ethyl methacrylate (**N3-B2**).

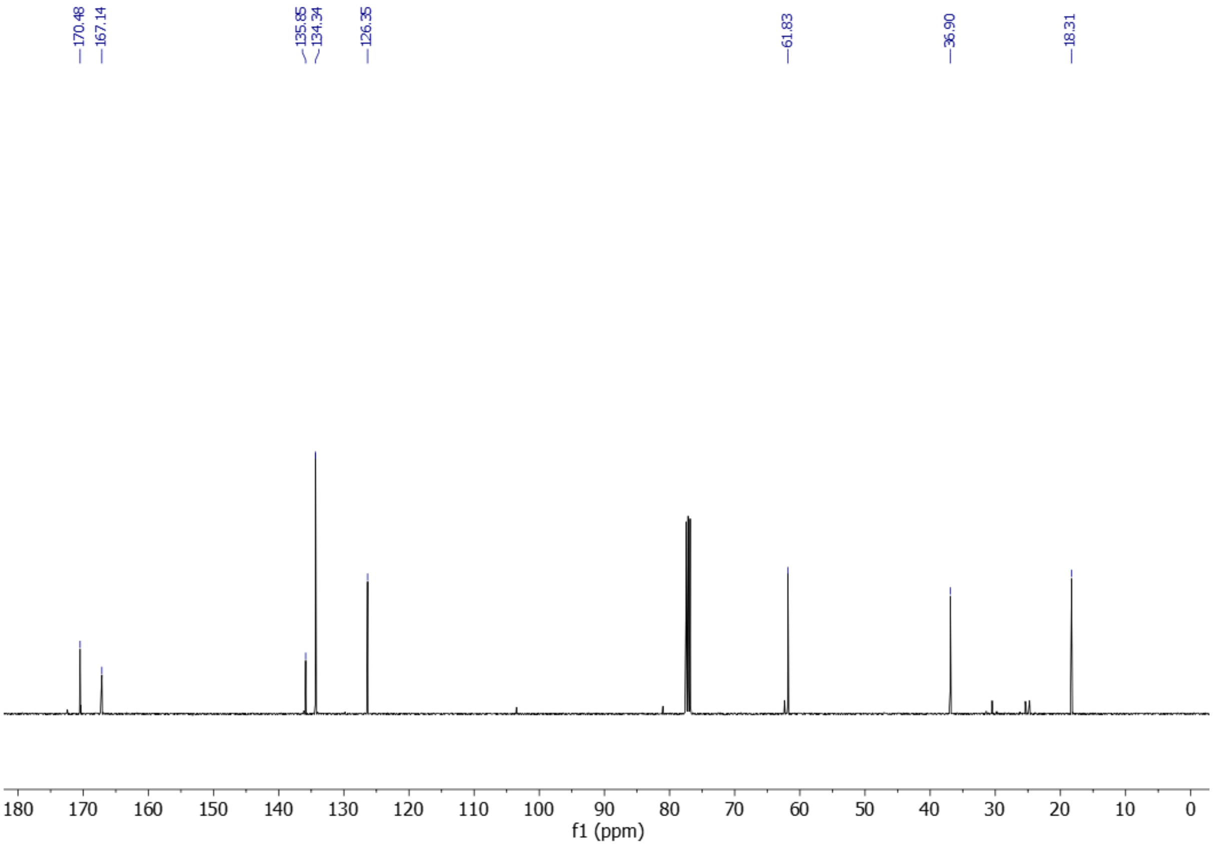

Supplementary Figure 79. ^{13}C NMR spectrum (101 MHz, $CDCl_3$) of 2-(2,5-dioxo-2,5-dihydro-1H-pyrrol-1-yl)ethyl methacrylate (**N3-B2**).

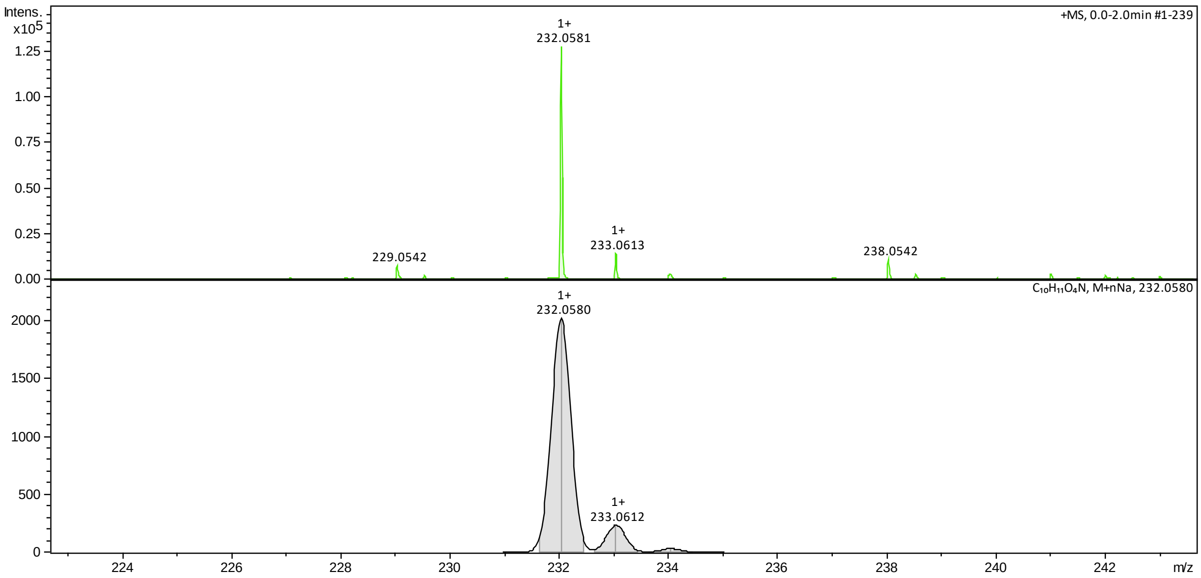

Supplementary Figure 80. ESI-MS (*m/z*) of 2-(2,5-dioxo-2,5-dihydro-1H-pyrrol-1-yl)ethyl methacrylate (**N3-B2**).

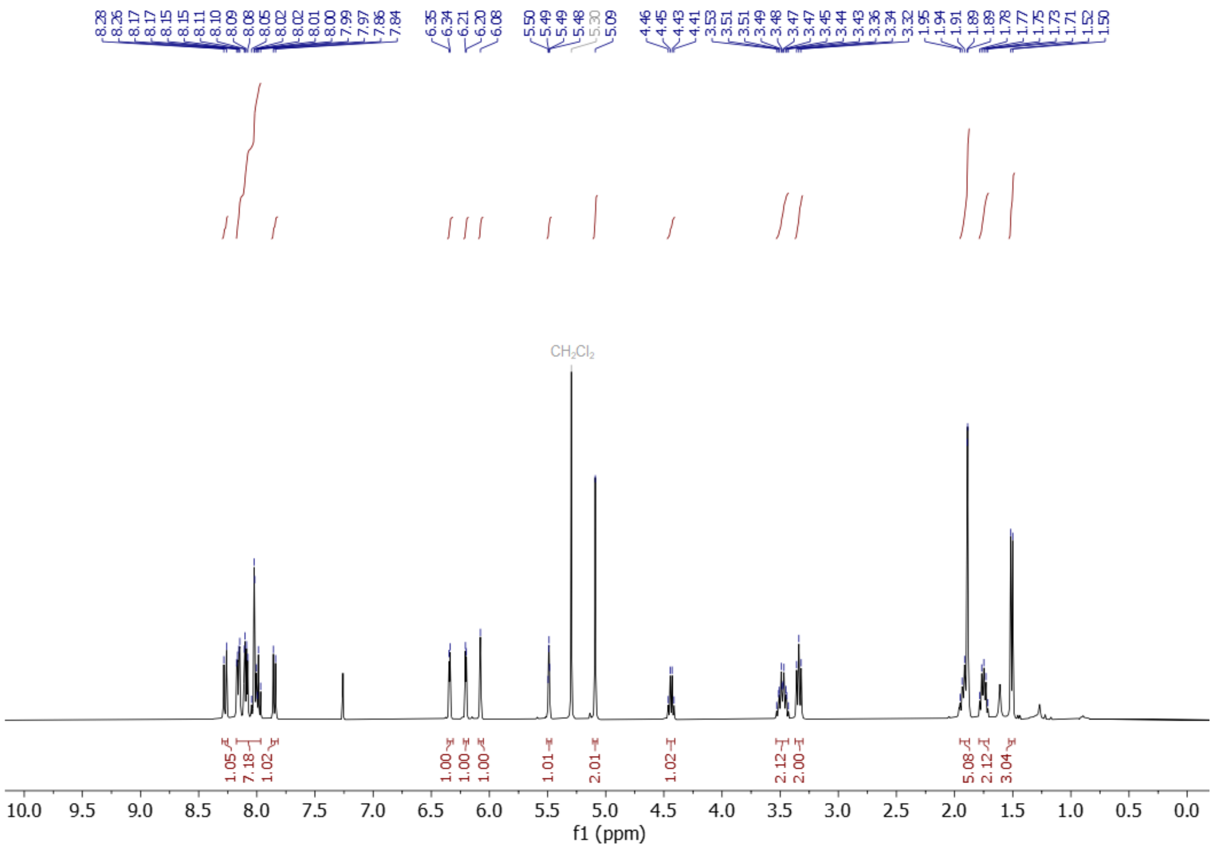

Supplementary Figure 81. ^{1}H NMR spectrum (400 MHz, $CDCl_3$) of (5-(1-(((4-(pyren-1-yl)butoxy)carbonyl)oxy)ethyl)furan-2-yl)methyl methacrylate (**N3-A4**).

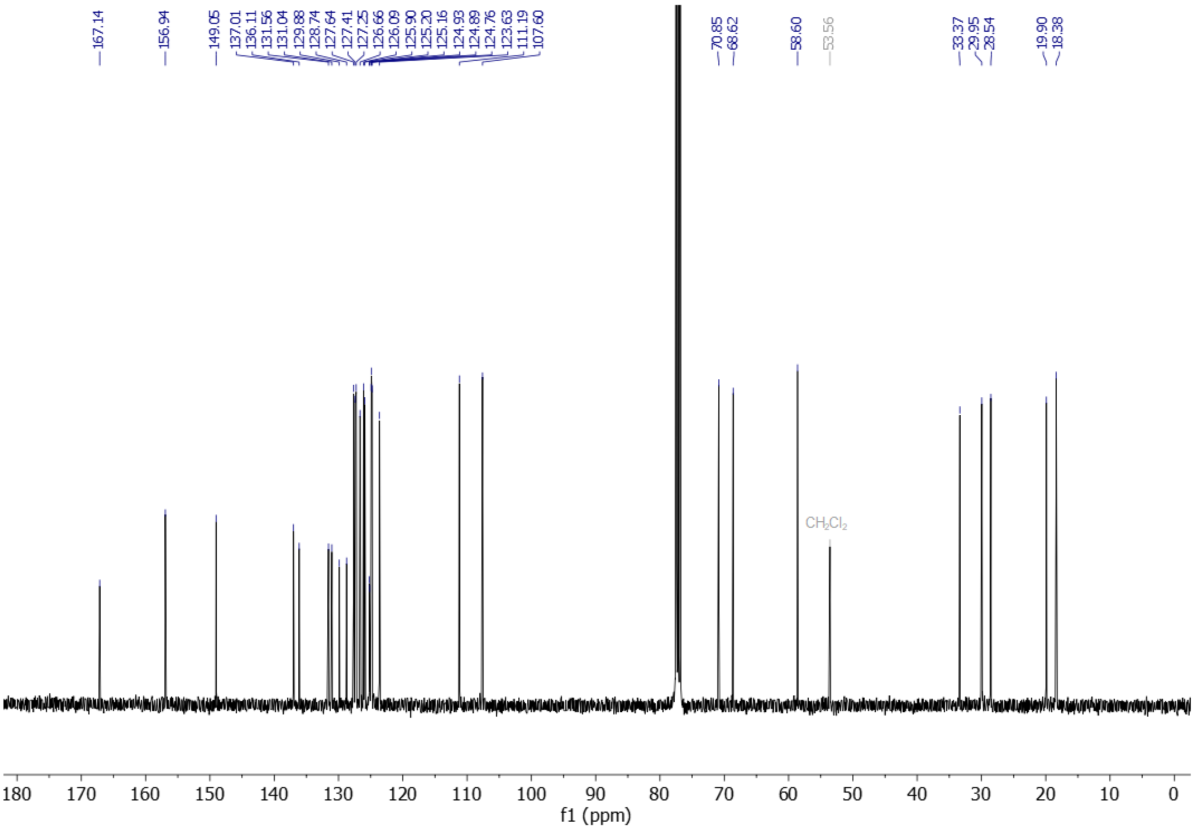

Supplementary Figure 82. ^{13}C NMR spectrum (101 MHz, $CDCl_3$) of (5-(1-(((4-(pyren-1-yl)butoxy)carbonyl)oxy)ethyl)furan-2-yl)methyl methacrylate (**N3-A4**).

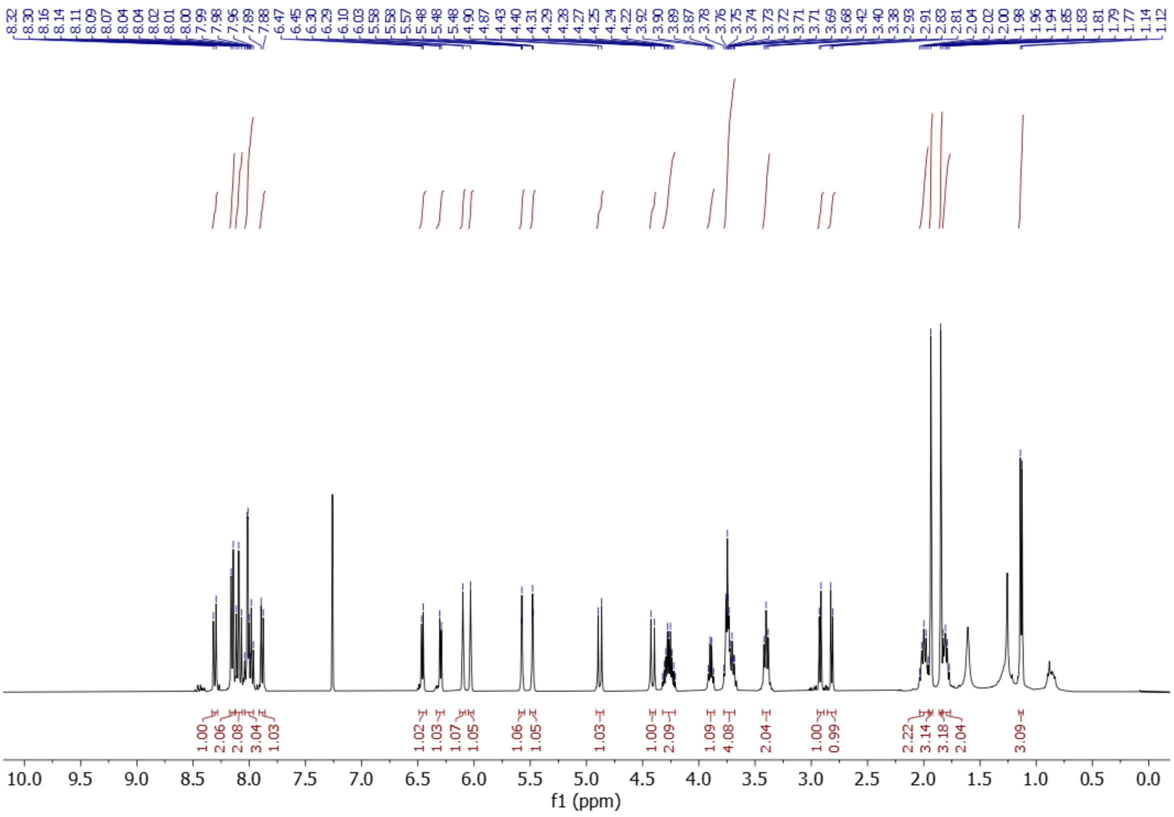

Supplementary Figure 83. ^{1}H NMR spectrum (400 MHz, $CDCl_3$) of (2-(2-(methacryloyloxy)ethyl)-1,3-dioxo-7-(1-(((4-(pyren-1-yl)butoxy)carbonyl)oxy)ethyl)-1,2,3,3a,7,7a-hexahydro-4H-4,7-epoxyisoindol-4-yl)methyl methacrylate (**N3-A5**).

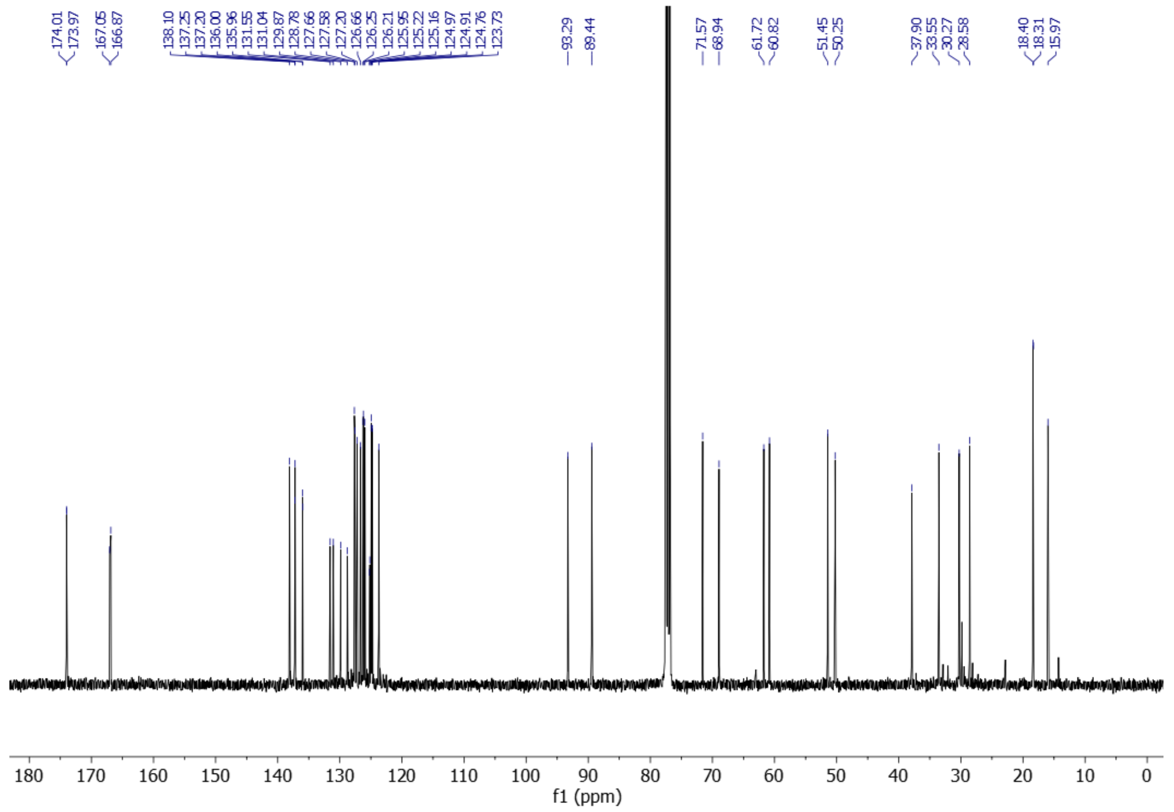

Supplementary Figure 84. ^{13}C NMR spectrum (101 MHz, $CDCl_3$) of (2-(2-(methacryloyloxy)ethyl)-1,3-dioxo-7-(1-(((4-(pyren-1-yl)butoxy)carbonyl)oxy)ethyl)-1,2,3,3a,7,7a-hexahydro-4H-4,7-epoxyisoindol-4-yl)methyl methacrylate (**N3-A5**).

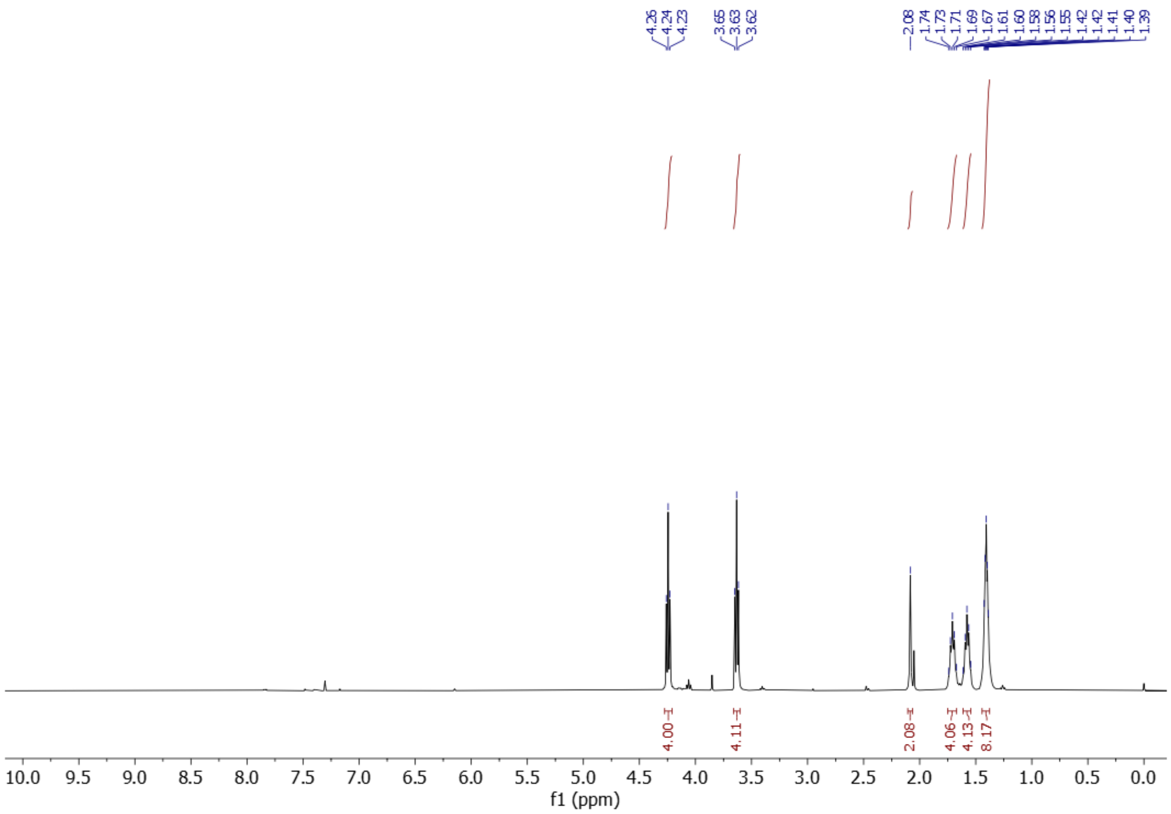

Supplementary Figure 85. ^{1}H NMR spectrum (400 MHz, $CDCl_3$) of bis(6-hydroxyhexyl) but-2-ynedioate (**N4-A2**).

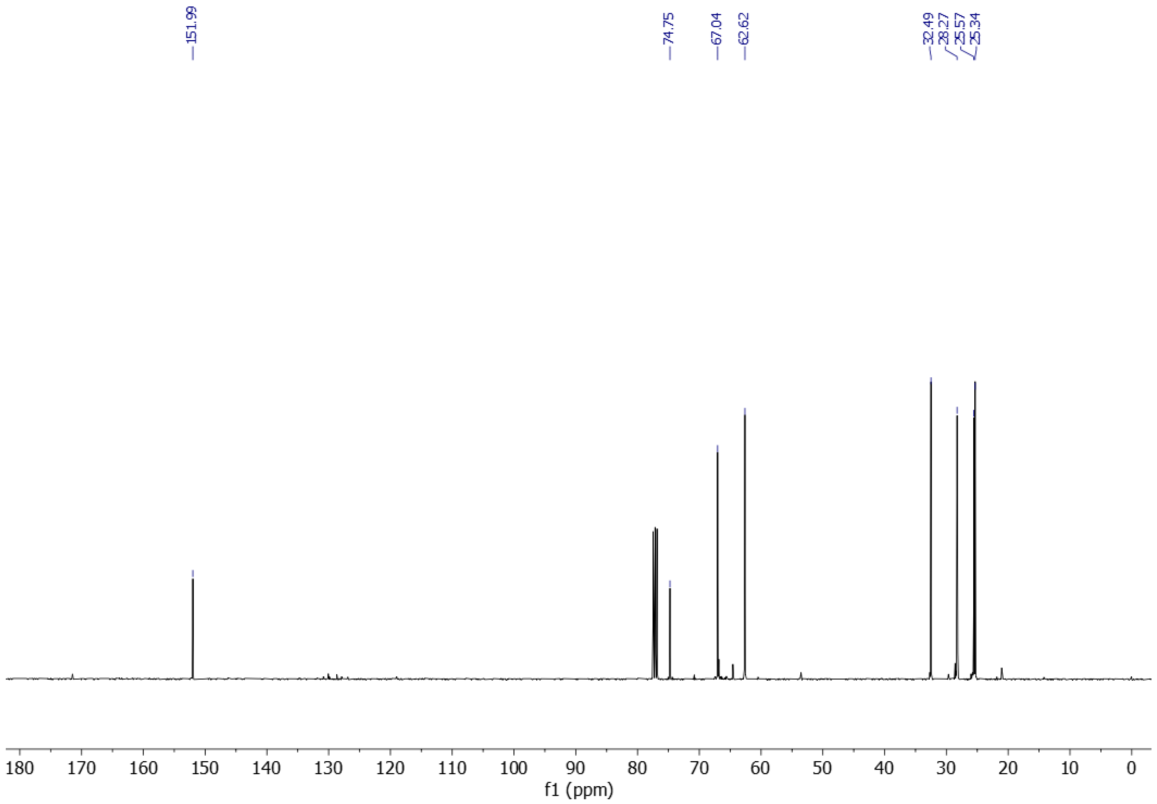

Supplementary Figure 86. ^{13}C NMR spectrum (101 MHz, $CDCl_3$) of bis(6-hydroxyhexyl) but-2-ynedioate (**N4-A2**).

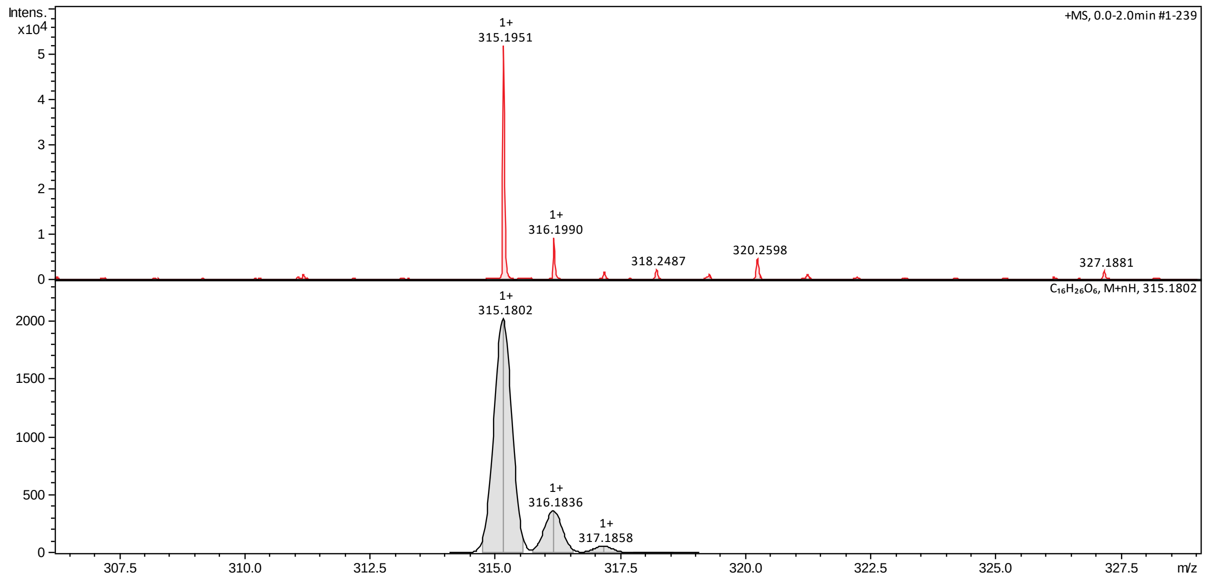

Supplementary Figure 87. ESI-MS (*m/z*) of bis(6-hydroxyhexyl) but-2-ynedioate (**N4-A2**).

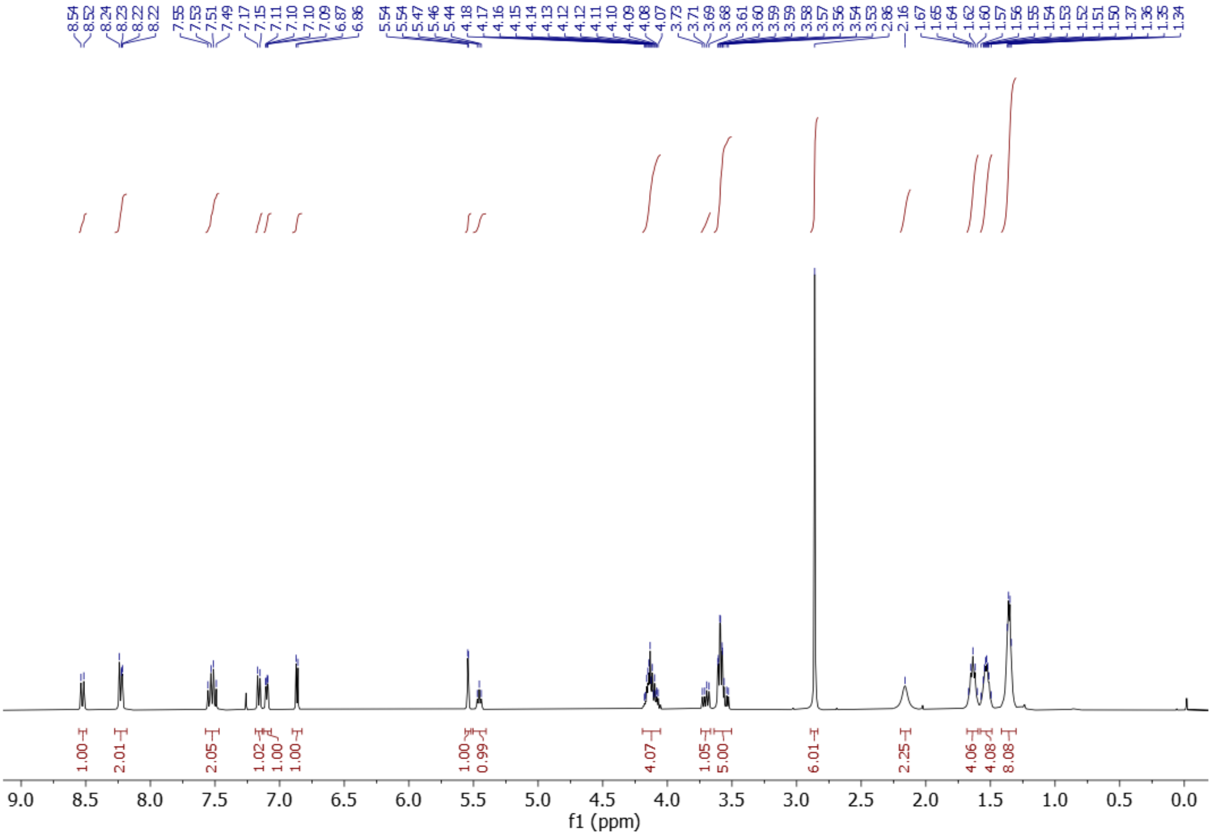

Supplementary Figure 88. ^{1}H NMR spectrum (400 MHz, $CDCl_3$) of bis(6-hydroxyhexyl) 1-(((5-(dimethylamino)naphthalene)-1-sulfonamido)methyl)-7-oxabicyclo[2.2.1]hepta-2,5-diene-2,3-dicarboxylate (**N4-A3**).

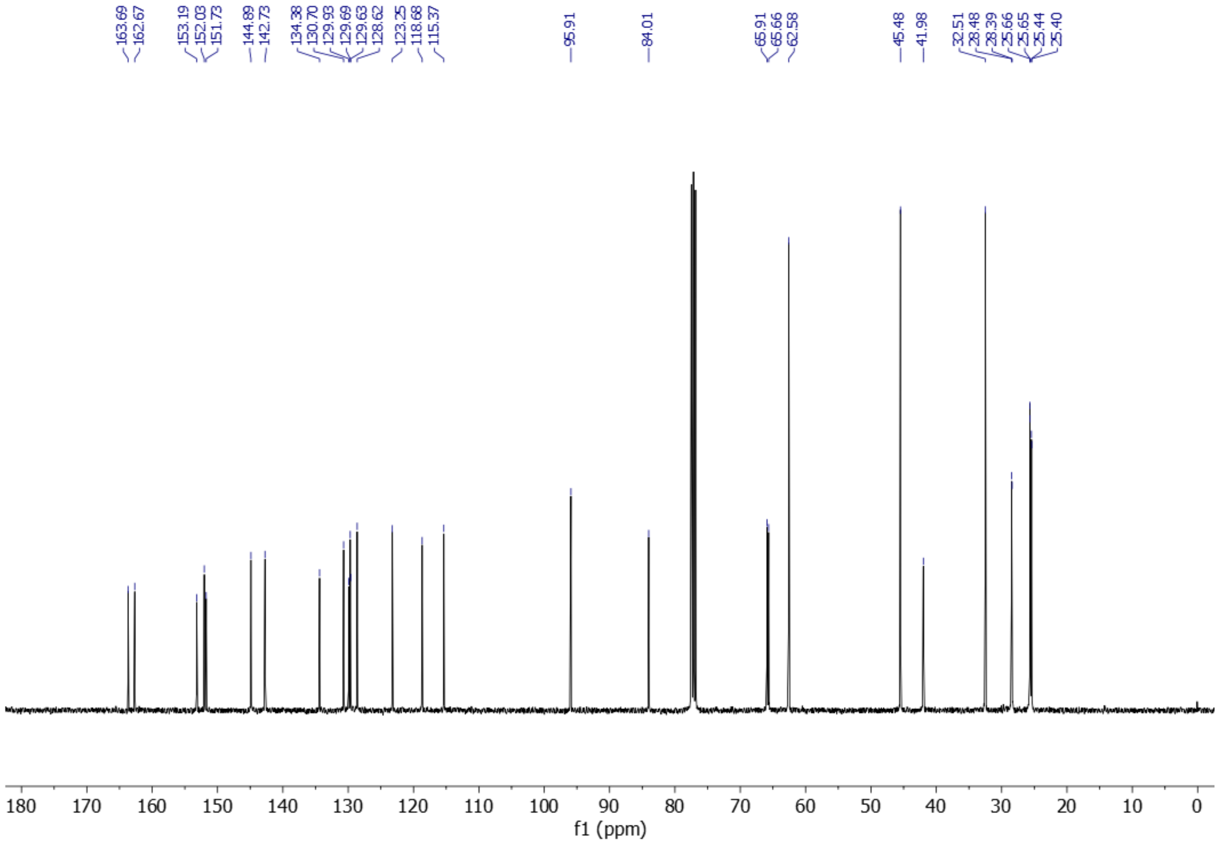

Supplementary Figure 89. ^{13}C NMR spectrum (101 MHz, $CDCl_3$) of bis(6-hydroxyhexyl) 1-(((5-(dimethylamino)naphthalene)-1-sulfonamido)methyl)-7-oxabicyclo[2.2.1]hepta-2,5-diene-2,3-dicarboxylate (**N4-A3**).

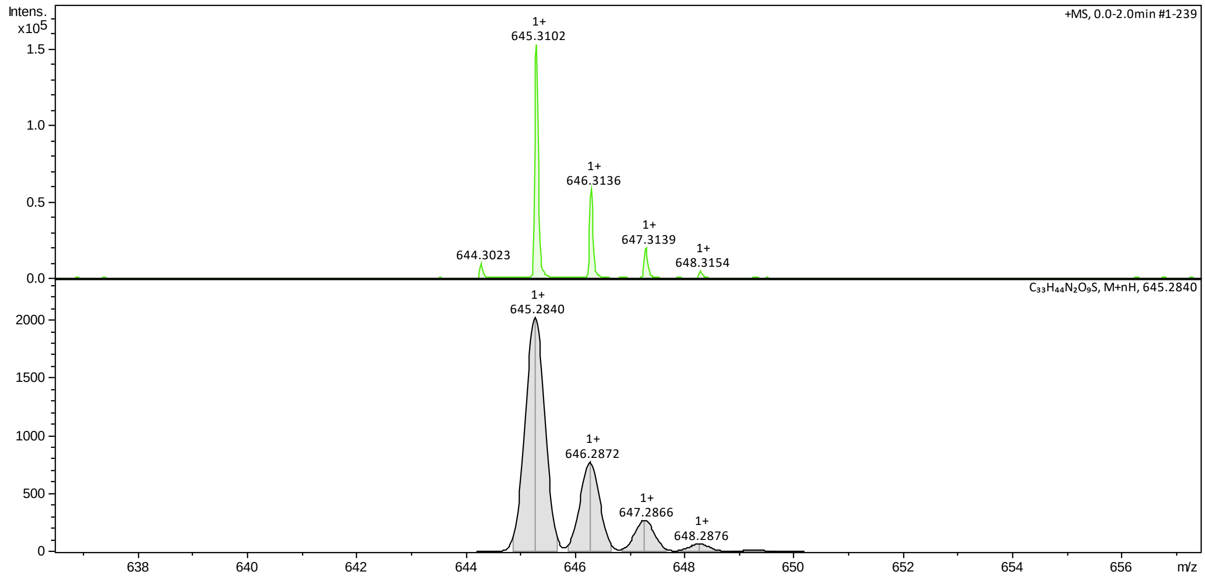

Supplementary Figure 90. ESI-MS (*m/z*) of bis(6-hydroxyhexyl) 1-(((5-(dimethylamino)naphthalene)-1-sulfonamido)methyl)-7-oxabicyclo[2.2.1]hepta-2,5-diene-2,3-dicarboxylate (**N4-A3**).

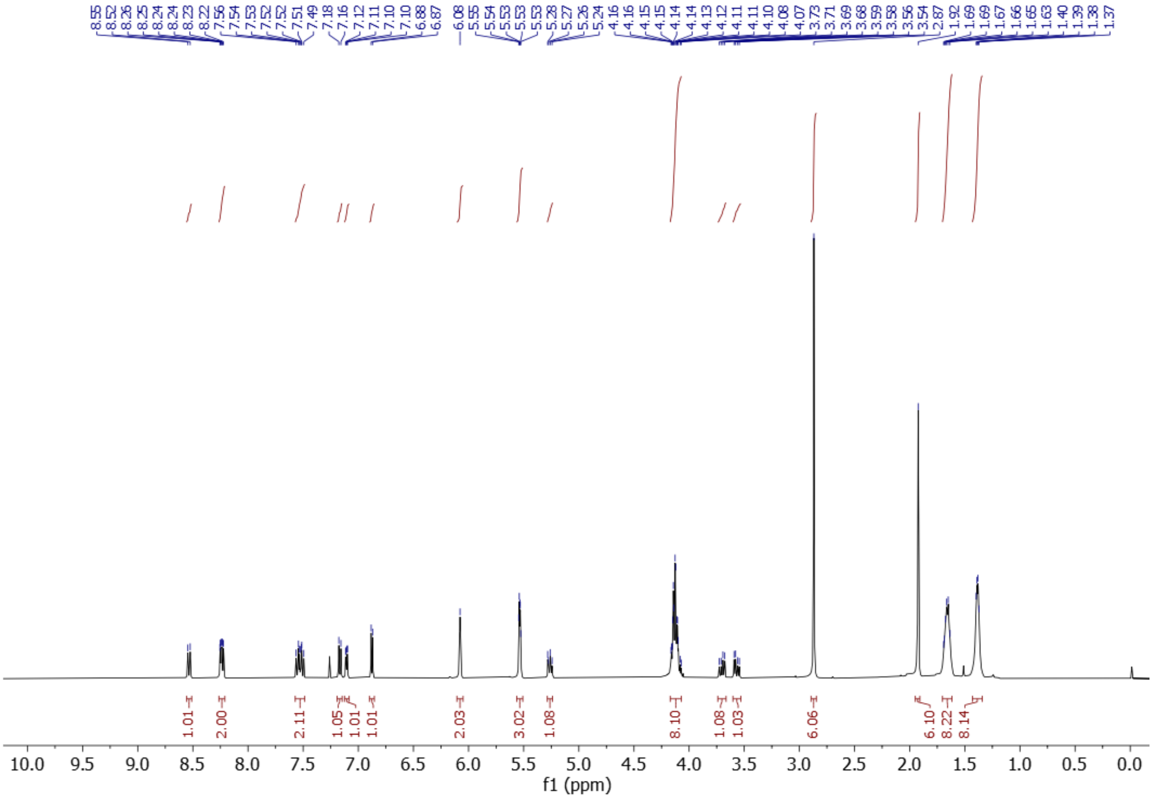

Supplementary Figure 91. ^{1}H NMR spectrum (400 MHz, $CDCl_3$) of bis(6-(methacryloyloxy)hexyl) 1-(((5-(dimethylamino)naphthalene)-1-sulfonamido)methyl)-7-oxabicyclo[2.2.1]hepta-2,5-diene-2,3-dicarboxylate (**N4-A4**).

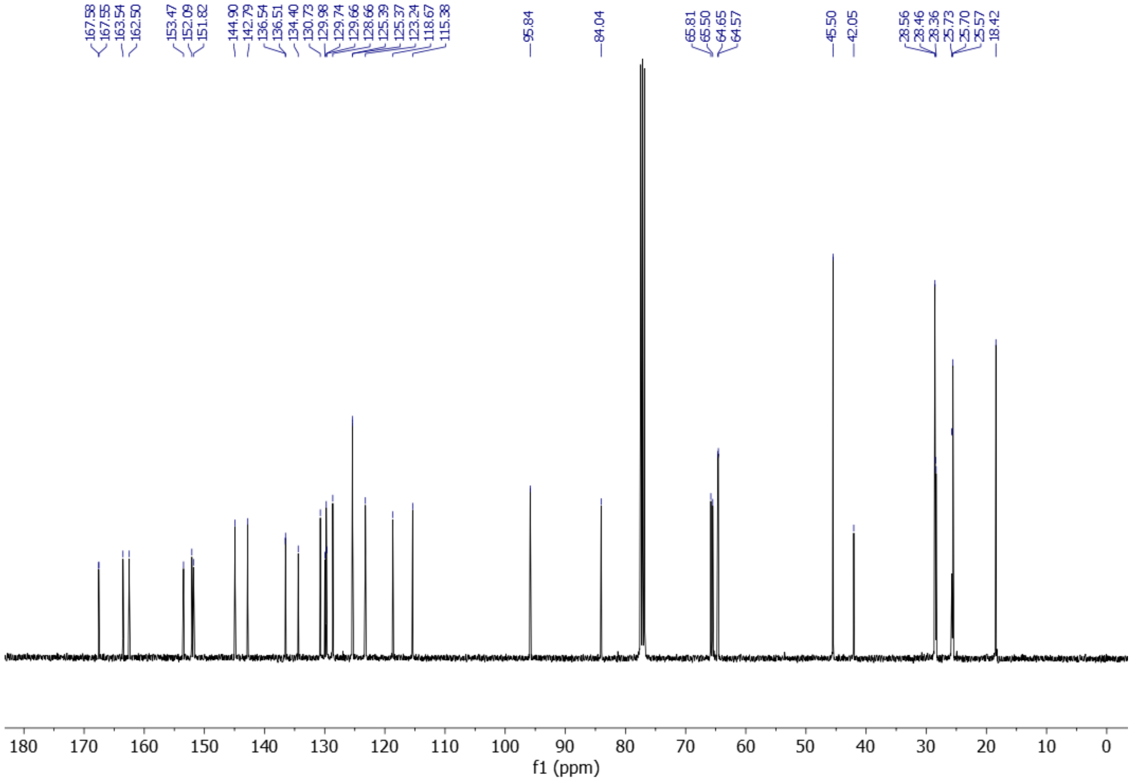

Supplementary Figure 92. ^{13}C NMR spectrum (101 MHz, $CDCl_3$) of bis(6-(methacryloyloxy)hexyl) 1-(((5-(dimethylamino)naphthalene)-1-sulfonamido)meth-yl)-7-oxabicyclo[2.2.1]hepta-2,5-diene-2,3-dicarboxylate (**N4-A4**).

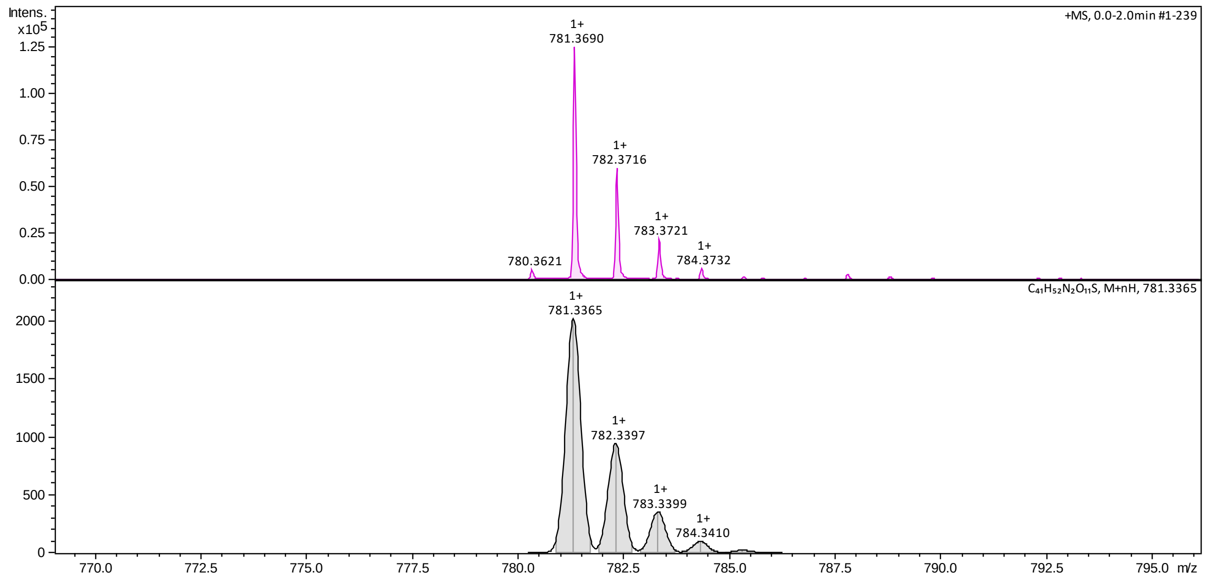

Supplementary Figure 93. ESI-MS (*m/z*) of bis(6-(methacryloyloxy)hexyl) 1-(((5-(dimethylamino)naphthalene)-1-sulfonamido)meth-yl)-7-oxabicyclo[2.2.1]hepta-2,5-diene-2,3-dicarboxylate (**N4-A4**).

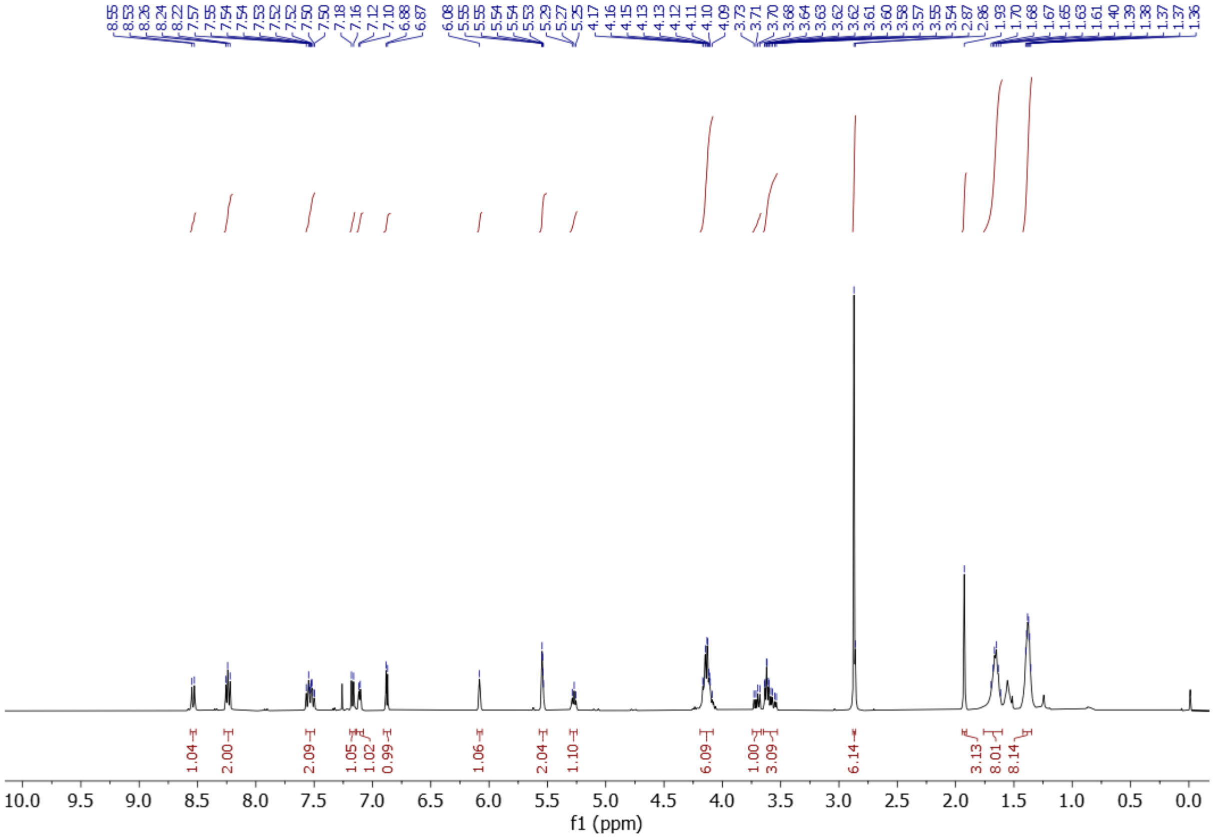

Supplementary Figure 94. ^{1}H NMR spectrum (400 MHz, $CDCl_3$) of 2-(6-hydroxyhexyl) 3-(6-(methacryloyloxy)hexyl) 1-(((5-(dimethylamino)naphthalene)-1-sulfonamido)methyl)-7-oxabicyclo[2.2.1]hepta-2,5-diene-2,3-dicarboxylate (**N4-B4**).

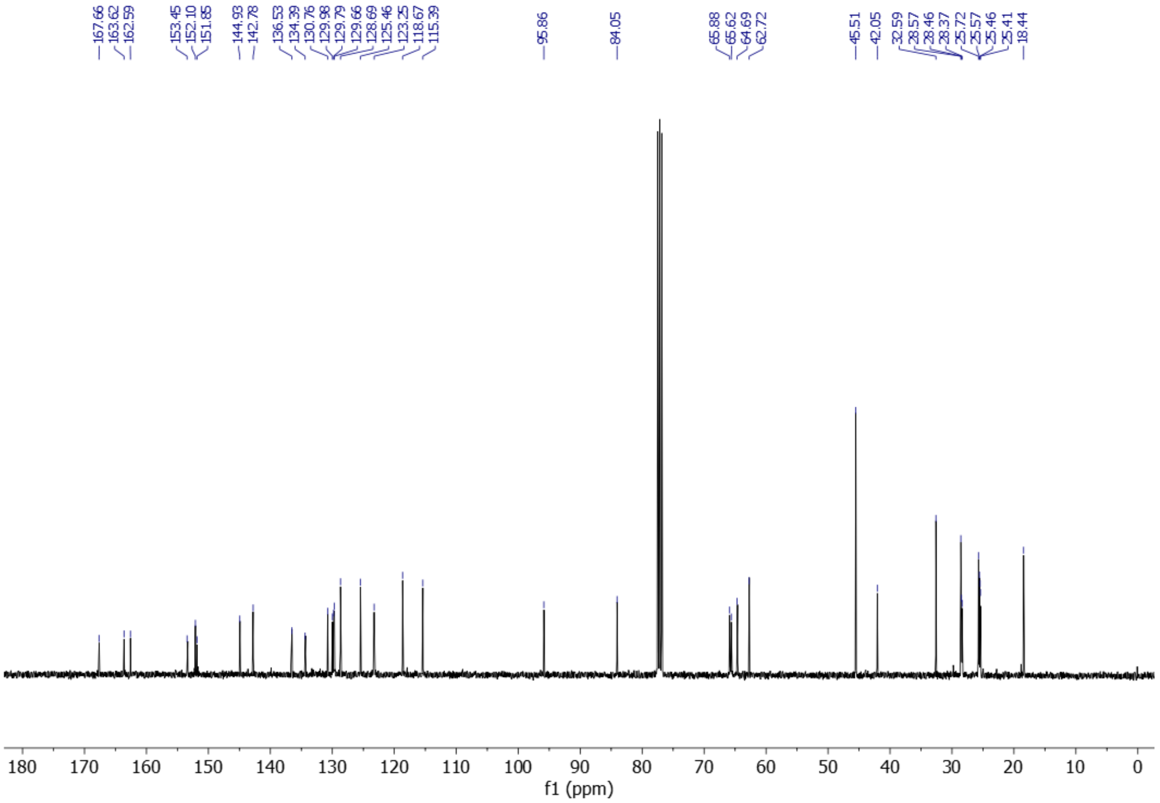

Supplementary Figure 95. ^{13}C NMR spectrum (101 MHz, $CDCl_3$) of 2-(6-hydroxyhexyl) 3-(6-(methacryloyloxy)hexyl) 1-(((5-(dimethylamino)naphthalene)-1-sulfonamido)methyl)-7-oxabicyclo[2.2.1]hepta-2,5-diene-2,3-dicarboxylate (**N4-B4**).

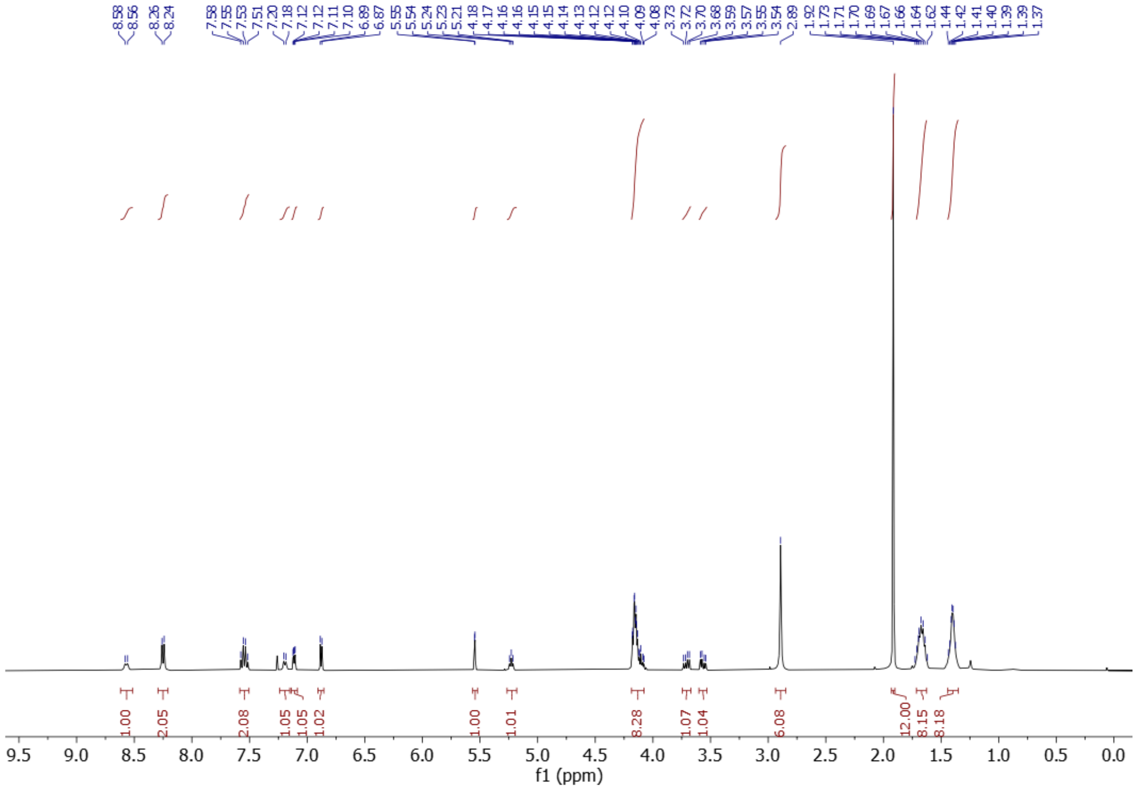

Supplementary Figure 96. ^{1}H NMR spectrum (400 MHz, $CDCl_3$) of bis(6-((2-bromo-2-methylpropanoyl)oxy)hexyl) 1-(((5-(dimethylamino)naphthalene)-1-sulfonamido)methyl)-7-oxabicyclo[2.2.1]hepta-2,5-diene-2,3-dicarboxylate (**N4-C4**).

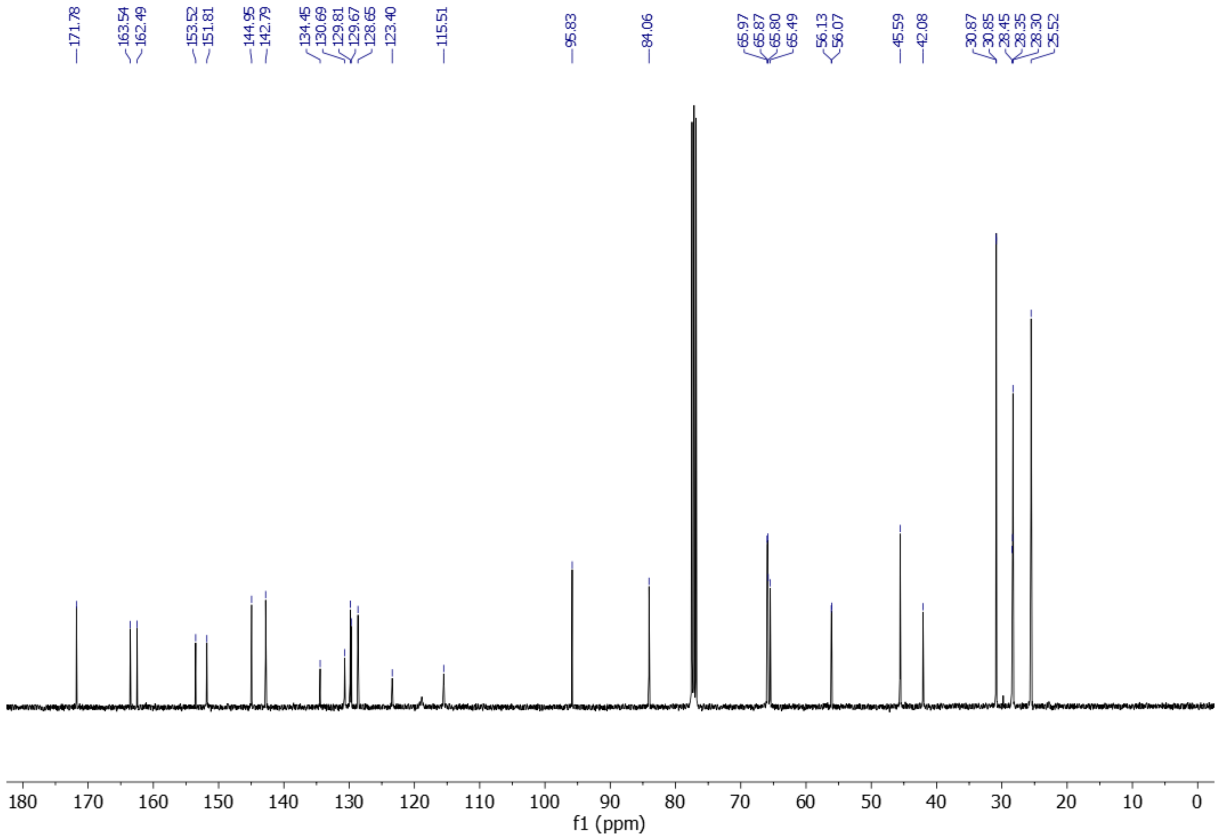

Supplementary Figure 97. ^{13}C NMR spectrum (101 MHz, $CDCl_3$) of bis(6-((2-bromo-2-methylpropanoyl)oxy)hexyl) 1-(((5-(dimethylamino)naphthalene)-1-sulfonamido)methyl)-7-oxabicyclo[2.2.1]hepta-2,5-diene-2,3-dicarboxylate (**N4-C4**).

6. Supplementary tables

Supplementary Table 1. The US intensity values corresponding to ultrasonic amplitude.

Ultrasonic amplitude	35%	53%	75%	99%
Sound Intensity (*I*)	1.0 W/cm^2	3.0 W/cm^2	6.0 W/cm^2	12.0 W/cm^2

Supplementary Table 2. HIFU transducers essential characteristics and mechanical index of 0.68 MHz, 1.52 MHz, and 2.6 MHz.

Frequency	Focal sound intensity (*I*)	Acoustic power	Mechanical index (MI)
0.68 MHz	8.4 W/cm^2	1.3 W	0.6
	33.6 W/cm^2	5.4 W	1.2
	75.5 W/cm^2	12.1 W	1.8
	134.2 W/cm^2	21.5 W	2.4
1.52 MHz	8.4 W/cm^2	0.1 W	0.4
	33.6 W/cm^2	0.5 W	0.8
	75.5W/cm^2	1.1 W	1.2
	134.2 W/cm^2	1.9 W	1.6
2.6 MHz	8.4 W/cm^2	0.04 W	0.3
	33.6 W/cm^2	0.2 W	0.6
	75.5W/cm^2	0.4 W	0.9
	134.2 W/cm^2	0.7 W	1.2

Supplementary Table 3. Data points measured to plot **Figure 3I** of the manuscript. All values mean ± SD from the mean.

Concentration (µM)	Control (PBS)	PMBs	PMBs fragments	CPT	CPT+US	PMBs+US (15 min)
32	100.1 ± 2.0	101.0± 0.4	68.0 ± 10.9	1.9 ± 0.7	0.6 ± 0.3	4.2 ± 0.3
16	99.3 ± 1.0	100.4± 0.7	86.9 ± 9.1	8.7 ± 0.4	6.8 ± 1.5	22.8 ± 1.9
8	100.7± 1.5	100.3± 0.8	95.8 ± 5.5	26.0 ± 2.7	25.3 ± 2.7	36.6 ± 2.9
4	99.8 ± 0.6	100.5± 1.0	94.4 ± 7.4	37.9 ± 3.8	41.4 ± 2.9	42.3 ± 4.7
2	100.4 ± 5.0	100.6± 1.1	94.7 ± 6.2	44.0 ± 1.0	47.5 ± 2.5	47.2 ± 4.6
1	97.8 ± 1.3	99.2± 1.4	95.1 ± 5.8	49.2 ± 1.5	53.3 ± 1.6	49.8 ± 7.5
0.5	98.7 ± 1.5	101.8± 0.7	93.7 ± 6.5	51.3 ± 2.5	57.9 ± 2.3	54.7 ± 7.9
0.25	100.0 ± 2.5	100.4± 1.5	94.8 ± 6.8	58.0 ± 2.6	64.4 ± 2.4	67.3 ± 10.7
0.125	99.7 ± 2.3	99.4± 2.3	97.8 ± 5.1	72.2 ± 1.0	76.3 ± 2.7	87.2 ± 6.1
0.0625	96.3 ± 0.8	102.6± 1.0	94.8 ± 7.8	91.4 ± 0.5	92.2 ± 2.3	99.1 ± 1.2

Supplementary Table 4. Force-modified reactant and transition state potential energies as well as potential activation energies for the flex-activated release of furan under the impact of a pulling force. The values for the reactant and the transition state energy are listed in atomic units (Hartree) while the activation energies are noted in kcal/mol.

Force [nN]	Reactant energy	Transition state energy	Activation energy
**0.0**	-2082.42790	-2082.38016	30.0
**0.5**	-2082.57700	-2082.52960	29.7
**1.0**	-2082.72933	-2082.68271	29.3
**1.5**	-2082.88419	-2082.83886	28.4
**2.0**	-2083.04106	-2082.99699	27.7
**2.5**	-2083.20031	-2083.15718	27.1
**3.0**	-2083.36118	-2083.31917	26.4
**3.5**	-2083.52413	-2083.48446	24.9
**4.0**	-2083.68933	-2083.65043	24.4

Supplementary Table 5. Force-modified reactant and transition state potential energies as well as potential activation energies for the flex-activated release of furan under the impact of uniaxial pressure. The values for the reactant and the transition state energy are listed in atomic units (Hartree) while the activation energies are noted in kcal/mol.

Force constant [Nm^{-1}]	Reactant energy	Transition state energy	Activation energy
**0.0**	-2082.42790	-2082.38016	30.0
**0.5**	-2082.39365	-2082.34286	31.9
**1.0**	-2082.37132	-2082.31964	32.4
**1.5**	-2082.35234	-2082.29767	34.3
**2.0**	-2082.33344	-2082.27462	36.9
**2.5**	-2082.31564	-2082.25622	37.3
**3.0**	-2082.29854	-2082.23869	37.6
**3.5**	-2082.28254	-2082.22247	37.7
**4.0**	-2082.26697	-2082.20678	37.8
**4.5**	-2082.25177	-2082.19147	37.8
**5.0**	-2082.23680	-2082.17645	37.9
**5.5**	-2082.22202	-2082.16171	37.8
**6.0**	-2082.20742	-2082.14724	37.8
**6.5**	-2082.19311	-2082.13301	37.7
**7.0**	-2082.17898	-2082.11902	37.6
**7.5**	-2082.16502	-2082.10526	37.5
**8.0**	-2082.15121	-2082.09172	37.3
**8.5**	-2082.13764	-2082.07840	37.2
**9.0**	-2082.12421	-2082.06529	37.0
**9.5**	-2082.11092	-2082.05237	36.7
**10.0**	-2082.09778	-2082.03963	36.5
**11.0**	-2082.07188	-2082.01464	35.9
**12.0**	-2082.04655	-2081.99025	35.3
**13.0**	-2082.02162	-2081.96638	34.7
**14.0**	-2081.99712	-2081.94296	34.0
**15.0**	-2081.97301	-2081.91995	33.3
**16.0**	-2081.94934	-2081.89735	32.6
**17.0**	-2081.92597	-2081.87508	31.9
**18.0**	-2081.90294	-2081.85315	31.2
**19.0**	-2081.88026	-2081.83151	30.6
**20.0**	-2081.85784	-2081.81016	29.9
**30.0**	-2081.64858	-2081.61044	23.9
**40.0**	-2081.48544	-2081.45456	19.4
**50.0**	-2081.33213	-2081.30602	16.4
**60.0**	-2081.19292	-2081.17102	13.7
**70.0**	-2081.06669	-2081.04893	11.1
**80.0**	-2080.95335	-2080.93914	8.9
**90.0**	-2080.85151	-2080.84035	7.0
**100.0**	-2080.75945	-2080.75093	5.3
**110.0**	-2080.67564	-2080.66943	3.9
**120.0**	-2080.59895	-2080.59465	2.7
**130.0**	-2080.52853	-2080.52576	1.7
**140.0**	-2080.46378	-2080.46230	0.9
**150.0**	-2080.40432	-2080.40394	0.2

7. Supplementary references

1. Dutta, K., Kanjilal, P., Das, R., & Thayumanavan, S. Synergistic Interplay of Covalent and Non-Covalent Interactions in Reactive Polymer Nanoassembly Facilitates Intracellular Delivery of Antibodies. *Angew. Chem. Int. Ed.* **60**, 1821-1830 (2021).

2. Liu, B., Wu, R., Gong, S., Xiao, H., & Thayumanavan, S. In situ formation of polymeric nanoassemblies using an efficient reversible click reaction. *Angew. Chem. Int. Ed.* **59**, 15135-15140 (2020).
