# Supplementary material for: Polymer microbubbles as universal platform to accelerate polymer mechanochemistry
Source: Nat Commun. 2025 Jun 25;16:5380. doi: 10.1038/s41467-025-60667-8 (PMC12198389; doi:10.1038/s41467-025-60667-8)
Supplement: Supplementary file 2 — Description of Additional Supplementary Files [file 41467_2025_60667_MOESM2_ESM.pdf]

**Description of Additional Supplementary Files**

**File name:** Supplementary Data 1
**Description:** The atomic coordinates of electronic structure calculations.

**File name:** Supplementary Movie 1
**Description:** The preparation of polymeric microbubbles (PMB-MDF).

**File name:** Supplementary Movie 2
**Description:** The preparation of microgel (control sample for PMB-MDF).
